# Conductive Gels for Wearable and Attachable Healthcare Devices: Materials, Sensing Mechanisms, Interfaces and Applications

**DOI:** 10.3390/gels12100883

**Published:** 2026-09-30

**Authors:** Jinmo Jeong, Dongwuk Jung

**Affiliations:** 1 Department of Artificial Intelligence and Biomedical Engineering, The Catholic University of Korea, Bucheon-si 14662, Gyeonggi-do, Republic of Korea; jeongjm@catholic.ac.kr; 2 Department of Mechatronics Engineering, Chonnam National University, 50, Daehak-ro, Yeosu-si 59626, Jeonnam-Gwangju, Republic of Korea

**Keywords:** conductive gels, skin–gel interfaces, gel–device interfaces, wearable healthcare devices, attachable healthcare devices

## Abstract

Wearable and attachable healthcare devices provide valuable insights across a broad range of applications, from health monitoring and disease diagnosis to the assessment of daily human activities. For these devices, both the formation of high-performance electrodes and mechanical, electrical, and physiological stability at human-device interfaces are critically important. Conductive gels, which combine deformable polymer networks with electrical conductivity, show considerable potential as functional materials for such applications. However, existing reviews have often considered material properties, interfacial behavior, and device reliability separately. This review examines hydrogels, ionogels, and organogels by linking material design, stimulus- and analyte-responsive sensing, biological and device interfaces, and healthcare applications. This integrated perspective emphasizes evaluating conductive gels within complete healthcare devices, where material properties, biological interfaces, and device integration jointly determine practical performance. Such evaluation needs to address signal quality and the stability of the assembled system during deformation, environmental exposure, and prolonged use. Future progress will require reproducible device fabrication and standardized validation tailored to the intended application, with systematic assessment of long-term biosafety and alignment with relevant regulatory requirements. Integration with flexible electronics, intelligent data processing, and feedback control may further enable reliable and responsive healthcare platforms.

## 1. Introduction

Wearable and attachable healthcare devices provide opportunities to acquire various forms of health-related information, including physiological, biomechanical, and biochemical data, during daily activities and over extended periods [1,2]. Depending on their configurations, these systems can monitor electrophysiological signals such as electrocardiogram (ECG), electromyogram (EMG), electroencephalogram (EEG), and electrooculogram (EOG); mechanical and physiological variables such as arterial pulse, respiration, joint motion, and plantar loading; and cutaneous or biochemical variables such as temperature, skin-surface humidity, sweat constituents, and wound-related conditions [3,4,5]. Reliable acquisition of such signals requires more than the miniaturization of conventional electronics. Body-facing components need to establish stable contact with soft, curved, and continuously moving biological surfaces while limiting discomfort and motion-induced disturbance [1,6]. Conventional rigid electrodes and sensing materials may resist natural deformation or form intermittent contact gaps, producing effects such as increased interfacial impedance, unstable signal transmission, or detachment during movement [6,7]. These limitations have motivated the development of soft and deformable materials that can maintain functional contact with the body under realistic wearing conditions [6,8].

Conductive gels provide a versatile material platform for this purpose. Within the scope of this review, conductive gels generally consist of a three-dimensional polymer network containing a liquid phase and support ionic, electronic, or mixed charge transport [8,9,10]. According to the liquid phase and solvent composition, the platforms considered here encompass water-containing hydrogels; ionic-liquid- or deep-eutectic-solvent-containing ionogels and eutectogels; and organic-solvent- or mixed-solvent-containing organogels and organohydrogels [11,12,13]. Electrical conductivity can be introduced or enhanced using components such as mobile ions, polyelectrolytes, conductive polymers, metals, carbon-based materials, MXenes, and combinations thereof [9,14]. The coexistence of a deformable polymer network and a mobile liquid phase allows these materials to combine solid-like structural integrity with mechanically compliant charge transport. In hydrated systems, the liquid-filled network can additionally facilitate the transport of species such as ions, small molecules, and biochemical analytes [8,11]. By varying material and structural parameters, including the polymer composition, crosslinking architecture, solvent, conductive phase, and microstructure, properties such as softness, stretchability, toughness, adhesion, conductivity, permeability, self-healing capability, and environmental stability can be adjusted for different device requirements [10,14,15].

However, conductive gels are not the only soft conductive materials available for body-facing electronics. Recent liquid metal/elastomer systems have achieved high electrical conductivity while remaining functional at strains exceeding 1000% [16,17]. Related liquid metal-based platforms have also shown good biocompatibility for bio-integrated use [18]. These advances demonstrate that high conductivity, extreme stretchability, and suitability for bio-integrated applications are not exclusive to conductive gels. Therefore, the distinctive value of conductive gels lies not in universally higher conductivity or stretchability, but in the additional transport and interfacial functions enabled by their liquid-filled polymer networks. Depending on their composition, conductive gels can support ionic or mixed charge transport and, particularly in hydrated formulations, facilitate the movement of ions and biochemical species through soft, conformal interfaces with biological surfaces [19].

These characteristics allow conductive gels to perform diverse functions within wearable and attachable devices rather than serving only as conventional sensing materials. They may serve, for example, as compliant bioelectrodes that couple ionic bioelectrical activity at biological surfaces to electronic recording systems, deformable sensing elements that change their electrical characteristics under mechanical or environmental stimuli, matrices that collect analytes and support chemical or biochemical transduction, or stretchable electrodes and ionically active layers in device configurations such as capacitive, ionotronic, and triboelectric systems [8,9,20,21]. Accordingly, changes in factors such as gel geometry, conductive network connectivity, ion distribution, interfacial capacitance, temperature- or moisture-dependent transport, and chemical or biochemical reactions can be converted into outputs such as resistance, capacitance, potential, current, and impedance [9,20,22]. This functional diversity enables conductive gels to support measurements including physical, electrophysiological, and biochemical signals, as well as multimodal devices in which complementary signals are acquired from the same body region or integrated platform [3,9,19,22].

Nevertheless, attractive bulk properties do not necessarily ensure stable performance after a conductive gel is incorporated into a healthcare device. The effective performance of the device depends on the coupled material-interface-device system. At the skin–gel interface, factors such as conformability, adhesion, contact area, water content, and gas or moisture transport can affect mechanical stability, interfacial impedance, wearing comfort, signal fidelity, and other performance characteristics [4,7,23]. The biological response may also depend on variables including the complete gel formulation and the duration and conditions of contact [4,6]. At the gel–electrode or gel–device interface, limitations such as insufficient bonding, mechanical mismatch, relative sliding, and localized stress can lead to failure modes including delamination, unstable electrical connection, and damage to adjacent components [24,25]. Encapsulation introduces another interface because it must protect the gel and regulate solvent exchange without excessively restricting deformation or impairing contact with the target surface [26,27]. These requirements vary across applications such as epidermal bioelectrodes, joint-motion or plantar-pressure sensors, sweat-monitoring patches, wound-contacting systems, and tissue-contacting devices. Consequently, the interpretation of material and interfacial performance depends on considerations such as the intended attachment site, measured signal, deformation mode, and operating environment [2,6,19].

Long-term use further reveals limitations that may not be apparent from initial metrics or short-duration tests, such as initial conductivity, maximum stretchability, or brief sensing measurements. Repeated deformation modes, including stretching, compression, bending, and shear, can produce degradation phenomena such as mechanical hysteresis, fatigue, crack growth, conductive network disruption, resistance drift, and loss of sensitivity or signal reproducibility [10,28,29,30]. Environmental exposure may also alter the liquid phase and polymer network. For example, water evaporation can dehydrate and shrink hydrogels, subzero temperatures can induce freezing and embrittlement, and exposure to media such as sweat, wound exudate, and surrounding water can cause swelling, ion exchange, or leakage of conductive components [31,32]. Organogels, ionogels, and related nonaqueous or mixed-solvent systems can mitigate some limitations associated with aqueous gels through properties such as reduced volatility or improved temperature and moisture tolerance. Nevertheless, application-specific issues including solvent compatibility, leakage, interfacial behavior, and biological safety remain relevant [11,12,13]. Strategies such as dynamic or energy-dissipating networks, self-healing structures, solvent-retaining formulations, anti-freezing or anti-swelling designs, interfacial bonding, and compliant encapsulation can improve durability [10,26,28,32]. The practical reliability of a device therefore depends on evaluating factors such as mechanical and electrical stability, signal specificity, calibration validity, environmental resistance, interfacial integrity, biological compatibility, and related system-level performance under combinations of use conditions, including repeated deformation, prolonged attachment, and environmental exposure [2,4,19,22].

Previous reviews have established important foundations for this field. Broad reviews have examined conductive gels and their use in devices, including wearable and attachable systems, from perspectives such as conductive components, material properties, sensing mechanisms, electronic-skin functions, and wearable healthcare applications [9,14,15,33,34]. Other reviews have focused on topics such as mechanically robust networks and mechanical-design parameters, water-resistant gels for underwater sensing, and conductive hydrogel epidermal electrodes for electrophysiological monitoring [4,10,28,31]. Specialized sensing and fabrication areas, including pressure sensing [35], resistive strain sensing [29], piezoelectric and triboelectric devices [21,36], three-dimensional printing [37], and machine-learning-enabled hydrogel biosensors [22], have also been examined. Collectively, these studies demonstrate the breadth of conductive gel research, although their organizing frameworks commonly emphasize one or more aspects, such as a particular gel family, material property, transduction route, fabrication method, operating environment, or application subset. A complementary need therefore remains for a framework that follows conductive gels from material composition and sensing behavior through body and device integration to application-specific performance and long-term reliability.

**Figure 1:**
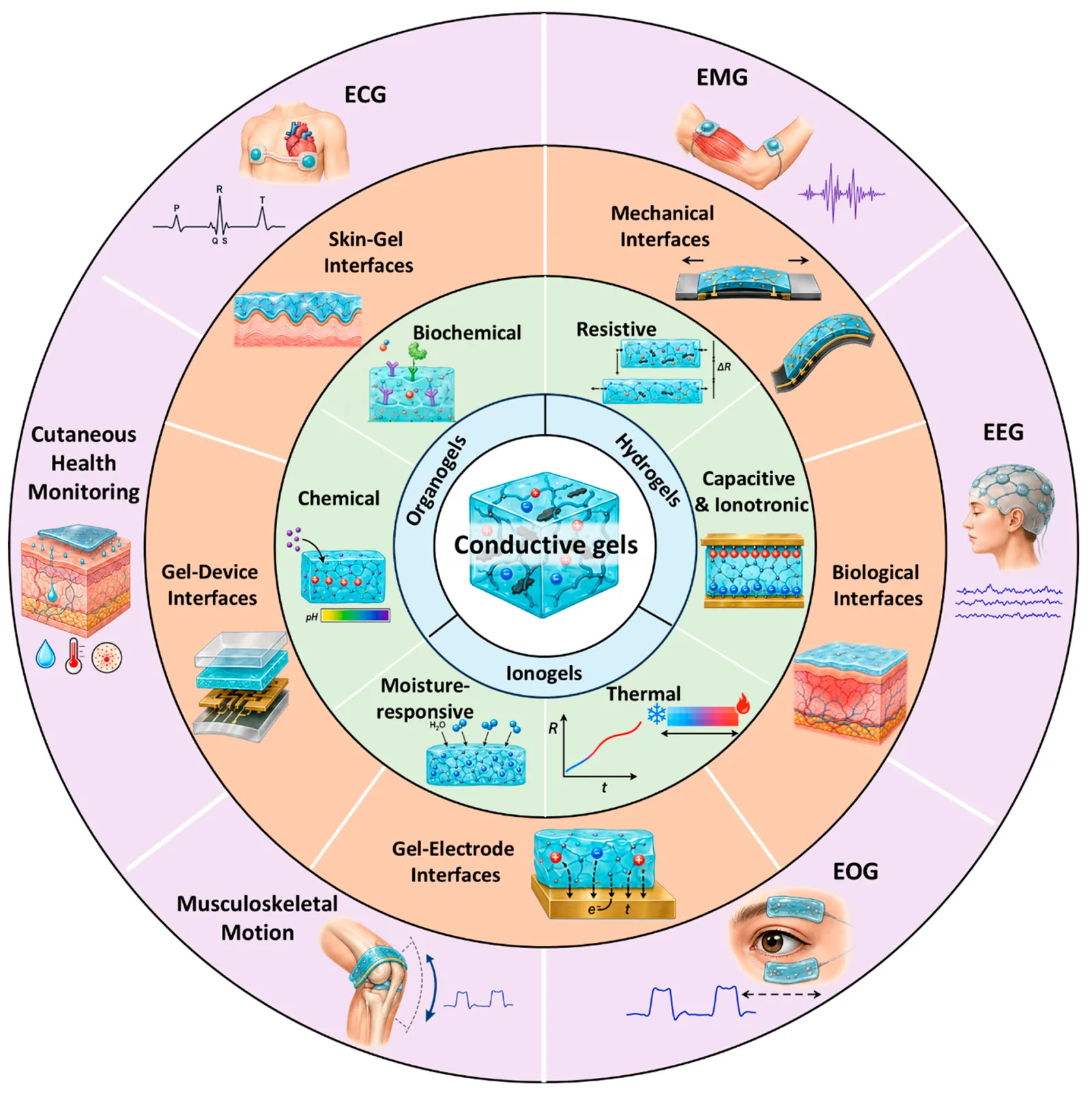
**Schematic overview of conductive gels for wearable and attachable healthcare devices, illustrating the material platforms, sensing mechanisms, interfaces, and representative applications.** The graphical icons in the schematics were generated using ChatGPT (GPT-5.6, OpenAI), while the overall organization and annotations were developed by the authors.

This review addresses this need through a material-mechanism-interface-device framework, as shown in the schematic overview in Figure 1. It relates the compositions and characteristic properties of the conductive gel platforms to sensing mechanisms such as resistive, capacitive and ionotronic, thermal and moisture-responsive, chemical and biochemical, and multimodal transduction. Their integration is then considered through interfaces with biological surfaces and adjacent device layers, including skin–gel, gel–electrode, gel–device, and encapsulation-related interactions. Representative applications are examined in areas such as cardiovascular and respiratory monitoring, musculoskeletal motion and rehabilitation, neurological and ocular monitoring and human–machine interfaces, and biochemical, cutaneous, and integrated monitoring. In this review, wearable devices refer to externally worn systems, whereas attachable devices refer to systems directly attached to skin or other biological tissues, with skin-adhered wearable patches belonging to both categories. Although the primary focus is on noninvasive wearable and attachable systems, this review also includes internal tissue-attachable implantable devices and minimally invasive systems, such as skin-attachable microneedles, to illustrate the functionality of conductive gels across diverse biological tissue-contact environments.

This article is organized as a narrative review rather than a systematic or bibliometric review. Literature was selected for direct relevance to the material-mechanism-interface-device framework, including recent peer-reviewed studies together with representative earlier studies that established specific material, sensing, interface, or device concepts. We did not perform a systematic literature survey or bibliometric frequency analysis to quantify the prevalence of particular materials or components across the full literature. Accordingly, statements regarding material-use trends are interpreted within the scope of the cited studies or review datasets.

Across these topics, particular attention is given to issues such as mechanical and electrical durability, environmental and interfacial stability, biological safety, system-level integration, and validation under realistic operating conditions. By linking these aspects, this review aims to clarify how conductive gels can progress from promising soft materials and proof-of-concept sensing elements toward dependable components of healthcare devices across these different configurations.

## 2. Conductive Gel Materials for Wearable and Attachable Healthcare Sensors

A conductive gel consists of a polymer network, a liquid phase, and charge carriers. The network holds the shape while the liquid phase allows ions or conductive components to move, so the gel is soft and deformable yet still transmits electricity [8]. This is why such gels suit wearable sensors, which must adhere closely to skin that is curved and constantly in motion.

Conductive gels can be described using two independent classification criteria. The first is the liquid phase: water-based hydrogels; ionogels and eutectogels containing ionic liquids or deep eutectic solvents; and organogels and organohydrogels containing organic solvents or mixed water-organic-solvent phases. The second is the dominant charge-transport mechanism: ionic, electronic, or mixed ionic–electronic conduction. In ionic conduction, mobile ions migrate through the liquid phase; in electronic conduction, electrons move through continuous pathways formed by conducting polymers or conductive fillers. When both pathways contribute appreciably, mixed ionic–electronic transport can occur.

These two classifications are independent; a particular gel family is not intrinsically restricted to a single transport mechanism. For example, a hydrogel may be predominantly ionically conductive when it contains dissolved salts, electronically conductive when a continuous conducting-polymer or filler network dominates, or mixed ionic–electronic when both pathways contribute. In this review, Section 2.1, Section 2.2 and Section 2.3 are organized by liquid phase because it strongly affects solvent retention, mechanical behavior, environmental stability, and biological compatibility. The conductive component is treated as a separate design variable, including dissolved salts, conducting polymers, and conductive particles. In the literature dataset compiled by Wu et al. [38], dissolved salts were represented more frequently than conducting polymers. This observation describes that dataset and is not intended as a quantitative prevalence estimate for the broader conductive gel literature.

**Figure 2:**
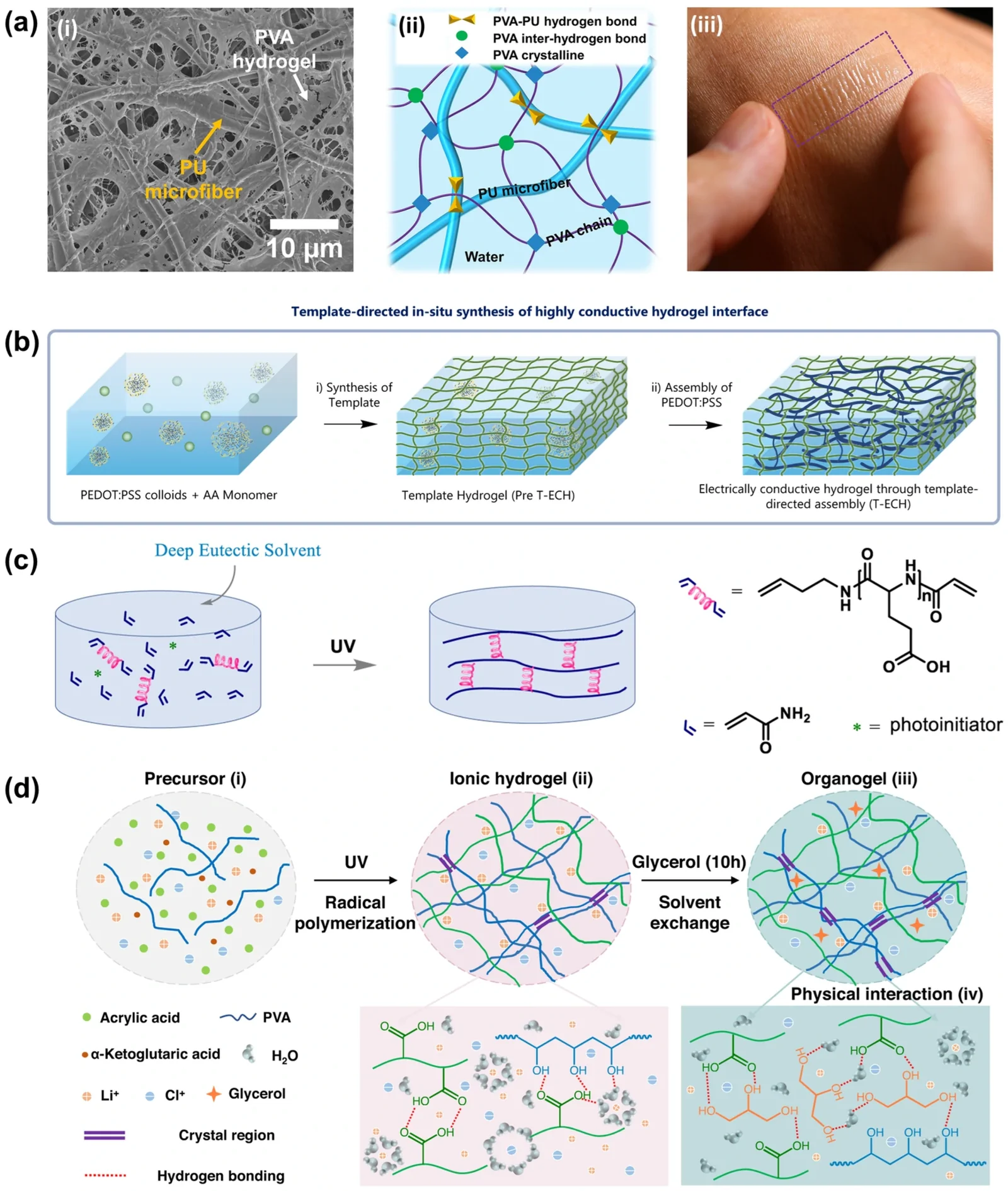
**Material design of the three conductive gel platforms.** (**a**) Network structure of a conductive hydrogel, (i) showing a scanning electron micrograph of the freeze-dried surface, (ii) the bonding mechanism between the polymer chains and the embedded microfibers, (iii) and the gel attached to skin. Adapted from Gao et al. [39] Nano-Micro Lett. 2023, 15, 139, under a CC BY 4.0 license. (**b**) Template-directed assembly of poly(3,4-ethylenedioxythiophene):poly(styrene sulfonate) (PEDOT:PSS), in which a colloidal dispersion is converted into a continuous conducting network by drying and annealing with dimethyl sulfoxide (DMSO). Adapted from Chong et al. [40] Nat. Commun. 2023, 14, 2206, under a CC BY 4.0 license. (**c**) Preparation of a eutectogel, in which monomer, peptide crosslinker and photoinitiator are dissolved in a deep eutectic solvent and photopolymerized so that the network forms within the liquid phase. Adapted from Zhang et al. [41] Nat. Commun. 2022, 13, 6671, under a CC BY 4.0 license. (**d**) Preparation of an organogel by free-radical polymerization followed by exchange of the liquid phase into glycerol, together with the physical interactions within the resulting gel. Adapted from Tang et al. [42] npj Flex. Electron. 28 July 2023, under a CC BY 4.0 license.

Representative material designs of the three platforms are summarized in Figure 2. The following subsections compare the three platforms, as summarized in Table 1. Conversion into sensor signals is treated in Section 3, and skin and device interface issues in Section 4.

**Table 1:** Comparison of conductive gel platforms.

Gel Platform	Main Liquid Phase	Representative Polymers	Representative Conductive Components	Representative Conductivity	Measurement Conditions	Refs
Hydrogel (2.1)	Water	PVA, PAAm; alginate, gelatin, chitosan, cellulose derivatives, silk fibroin, casein/caseinate, gellan gum	Salts (NaCl, LiCl, CaCl_2_); PEDOT:PSS, PANI, PPy; CNT and graphene, AgNW, MXene	Ionic: (a) 0.64 S∙m^−1^ (b) 2–8 S∙m^−1^ Conducting polymer: (c) 2.0–4.0 × 10^3^ S∙m^−1^ (d) 2.47 × 10^4^ S∙m^−1^	(a) Hydrated; two Pt electrodes, 40 mm separation; Keithley 6514; temperature not reported. (b) 25 °C and 60% RH; four-probe resistivity measurement. (c) Rehydrated in DI water or PBS; four-point probe; temperature not reported. (d) Swollen in DI water or PBS for 24 h; four-point probe with stainless-steel surface leads; temperature not reported.	(a) [43] (b) [44] (c) [45] (d) [40]
Ionogel/eutectogel (2.2)	Ionic liquid ([EMIM]^+^ family) or deep eutectic solvent (choline chloride based)	Polyacrylates, polyurethanes, PAAm	Intrinsic ions of the ionic liquid	Ionogel: (a) ~1 × 10^−2^ S∙m^−1^ (b) 0.009–0.264 S∙m^−1^ Eutectogel: (c) 0.382 S∙m^−1^	(a) [EMIM][OAc] ionogel film; two-electrode sandwich configuration; Keithley 2450; temperature not reported. (b) [EMIM]Cl ionogel; electrochemical impedance spectroscopy between two stainless-steel electrodes; temperature not reported. (c) ChCl/ethylene glycol eutectogel; room temperature; measurement method and electrode configuration not reported.	(a) [46] (b) [47] (c) [41]
Organogel/organohydrogel (2.3)	Glycerol (representative), ethylene glycol, or their mixtures with water	PVA, PAAm, gelatin	Salts (LiCl, NaCl, AlCl_3_); MXene	Ionic: (a) 0.124–0.454 S∙m^−1^ Ionic + electronic: (b) 14.82 S∙m^−1^	(a) Water/glycerol/LiCl organohydrogel; linear-sweep voltammetry; temperature and electrode configuration not reported. (b) Glycerol/water organohydrogel containing NaCl and PMMA@MXene; measurement method, electrode configuration, and temperature not reported.	(a) [48] (b) [49]

For consistency, all conductivity values in Table 1 are expressed in S∙m^−1^. These values are representative results reported in different source studies and under different measurement conditions, so quantitative comparisons should be interpreted cautiously. Conductivity can depend on temperature, hydration or solvent state, charge-carrier concentration, sample geometry, electrode configuration, and measurement method; these experimental conditions should therefore be considered when comparing values across studies.

### 2.1. Hydrogels

A hydrogel is a hydrophilic polymer network swollen with water. Hydrogels are widely used conductive gel platforms for wearable and attachable healthcare devices. Their water-swollen structure supports ion mobility, while their mechanical properties can be tuned through polymer composition and network architecture. This tunability can support mechanically compliant interfaces with biological tissues. However, the mechanical properties of tissues and cells vary widely with tissue type, structural hierarchy, deformation mode, strain rate, and measurement method, so a single universal modulus range is not appropriate. Because hydrogels provide a wet, ion-rich environment, they are used not only in skin-attachable devices but also as interface layers on devices in direct contact with biological tissue [8]. Their water-rich nature provides these advantages but also introduces challenges associated with dehydration and swelling.

Poly(vinyl alcohol) (PVA) and polyacrylamide (PAAm) are widely used representative synthetic matrix polymers for conductive hydrogels and are treated as such in relevant reviews [50,51]. A recount in the present review of the 28-entry literature table compiled by Wu et al. [38] gives 11 entries for the acrylamide family and 8 for PVA, with composite formulations counted under each constituent. These counts describe the dataset compiled by Wu et al. [38] and should not be interpreted as a quantitative prevalence estimate for the entire conductive hydrogel field. PVA can be physically crosslinked by freezing and thawing, whereas PAAm can be polymerized directly in water, making both matrices straightforward to process. Natural polymers such as alginate, gelatin, and chitosan are also incorporated [50], and their broader roles are discussed separately below.

Tying polymer chains together into a network is called crosslinking, and it falls into three types according to the nature of the bond. Physical crosslinking relies on weak interactions such as hydrogen bonds or polymer crystallites; the representative example is the freeze–thaw method for PVA, in which freezing and thawing an aqueous solution causes part of the chains to crystallize and act as junctions, requiring neither a crosslinker nor an initiator. Covalent crosslinking ties the chains chemically using a crosslinker such as N,N′-methylenebisacrylamide (MBAA); it is strong but does not recover once broken. Dynamic crosslinking uses bonds that break and reform, such as metal-ion coordination, and contributes to self-healing and energy dissipation. Real materials frequently combine all three. Pan et al. added tannic acid and LiCl to an aqueous PVA solution and obtained a conductivity of 0.64 S∙m^−1^ for the optimized composition after a single freeze–thaw cycle (−20 °C for 12 h, then thawing at room temperature), showing that physical crosslinking alone can give a practical gel [43]. The network can also be built from more than one component: Gao et al. mimicked the extracellular matrix of biological tissue with an interpenetrating network of polyurethane microfibers and PVA chains, which yields a gel that is ultrasoft yet mechanically reinforced [39]. The microstructure of such a network, the bonding between the polymer chains within it, and the gel attached to skin are illustrated in Figure 2a.

Strength and stretchability usually move in opposite directions: tying the chains tightly makes the gel stiff but reluctant to stretch, whereas tying them loosely makes it stretchable but easy to tear. The double network relaxes this conflict by layering two networks of different character, and the combination of calcium alginate with PAAm is the representative case. Sun et al. attributed the toughness of this system to a synergy of two mechanisms: when the gel is stretched, the weak ionic bonds of the alginate unzip first and dissipate energy, while the covalently crosslinked PAAm bridges cracks and preserves the memory of the initial state [52]. The unzipped ionic bonds re-zip once the load is removed, allowing repeated use. Bonds that break first in this way to absorb energy are commonly called sacrificial bonds. Later studies have applied this principle while tuning the composition, including the Ca^2+^ concentration [53].

Dissolving a salt. This is the representative route for introducing conductivity. A salt dissolved in water dissociates into cations and anions, which migrate through the water-filled space and carry the charge. What matters here is the size relationship between the mesh and the ions. The mesh of a hydrogel is roughly a few to a few tens of nanometers depending on the crosslinking conditions, whereas the diameter of a hydrated Na^+^ or Cl^−^ is under one nanometer. This is why the ions are not caught by the network and can move relatively freely.

Inorganic salts are widely used because they are readily available and come in many varieties, halogenated salts of metal ions in particular [50]; those reported include NaCl, LiCl, KCl, and CaCl_2_. Hong et al. compared NaCl, LiCl, and CaCl_2_ within the same PAAm matrix. At steady state under 25 °C and 60% relative humidity, conductivity was approximately 8 S∙m^−1^ for LiCl at 6 mol∙L^−1^, 4.5 S∙m^−1^ for CaCl_2_ at 4 mol∙L^−1^, and 2 S∙m^−1^ for NaCl at 6 mol∙L^−1^, whereas water loss followed a different order: approximately 0% for CaCl_2_, 10% for LiCl, and 50% for NaCl, against 85% without salt [44]. Because the three salts were evaluated at different molar concentrations, the observed differences reflect both salt identity and formulation conditions and should therefore be interpreted cautiously. These data are best viewed as formulation-specific results obtained within the same PAAm matrix and environmental conditions, rather than as a general indication of the relative conductivity or water-retention performance of the salts.

Salt concentration alone does not settle the outcome. Adding ions increases the number of carriers available, while tightening the crosslinking narrows the passage through which those carriers travel. Lai et al. showed that raising the LiCl concentration from 0 to 2 mol∙L^−1^ lowered the interfacial impedance by two orders of magnitude, whereas raising the MBAA crosslinker density from 0 to 5% raised it by two orders of magnitude [54]. Conductivity and mechanical strength must therefore be tuned together.

Conducting polymers. These are selected when higher conductivity is required. Polyaniline (PANI), polypyrrole (PPy), and poly(3,4-ethylenedioxythiophene): poly(styrene sulfonate) (PEDOT:PSS) are representative. All possess a conjugated π-electron system and pass from an insulating or semiconducting state to a conducting state on doping [51]. A conjugated backbone is one in which single and double bonds alternate along the chain. In such a structure the π-electrons are delocalized over the chain rather than bound to particular atoms, so they can travel along it. Conjugation alone gives only low conductivity; however, charge must be injected by doping before practical values are reached. In PEDOT:PSS, the PSS acts both as the dopant and as the agent that disperses the water-insoluble PEDOT.

What governs conductivity in this route is not how much is added but whether the conducting phase forms an unbroken, continuous path. Lu et al. prepared a hydrogel from PEDOT:PSS alone, with no other polymer; adding dimethyl sulfoxide (DMSO), drying and annealing the material, and then rehydrating it connects the PEDOT in a fibrous form, giving approximately 40 S∙cm^−1^ in deionized water and approximately 20 S∙cm^−1^ in phosphate-buffered saline [45]. Chong et al. assembled PEDOT:PSS along a poly(acrylic acid) (PAA) template so that it formed thin connected fibers instead of bulk aggregates, reaching 247 S∙cm^−1^ at a content of only about 1 wt% (Figure 2b) [40]. Both studies show that the formation of a continuous conductive network matters more than the loading itself.

Conductive particles. In this route, conductive particles are mixed into an insulating hydrogel. Adding a small quantity does not make the gel conduct. Only once the particles touch one another and form a path running from one side to the other does the gel as a whole become conductive, and this point is called the percolation threshold. It can be pictured as stepping stones across a stream: while the stones are sparse the stream cannot be crossed, but as stones are added a continuous path appears at some point, and conductivity rises sharply just after it. The more elongated the stones and the more evenly they are placed, the fewer are needed.

The fillers commonly used are carbon nanotubes (CNTs) and graphene, valued for their high specific surface area, conductivity, and stability; metal nanomaterials such as silver nanowires (AgNW) and MXene are also used [51]. These aggregate readily, so dispersion is the key issue. Zheng et al. used cellulose nanofibers as a dispersant to distribute graphene uniformly through a PVA hydrogel, and conductivity increased non-linearly with graphene content to a maximum of approximately 3.55 S∙m^−1^ [55]. Filler-based routes share a common weakness: where particles aggregate the conductive pathway is interrupted, and particles can be pulled out of the network during repeated deformation. Uniform dispersion and firm particle-polymer bonding are therefore the key issues in practical implementation.

Percolation is determined not only by filler loading but also by filler geometry and the resulting network architecture. High-aspect-ratio fillers such as CNTs can bridge larger distances at lower filler fractions, whereas aggregation can reduce effective connectivity even when the total filler content is high. Tringides et al. illustrated these effects in an alginate hydrogel containing graphene flakes and CNTs [56]. Freezing and lyophilization created a microporous matrix that concentrated the carbon additives within the pore walls, and graphene flakes connected with CNT bundles, increasing the probability of a percolating path. The microporous gel reached an electrical percolation threshold at approximately 0.9 wt% total carbon and achieved conductivities above 10 S∙m^−1^, with a maximum of approximately 35 S∙m^−1^, at less than 2 wt% carbon [56]. This example quantitatively shows that filler aspect ratio, dispersion, and spatial network formation can shift the loading required for continuous electronic transport.

The disadvantages of hydrogels arise from the same source as their advantages. Water evaporates over time. As the gel dries it loses elasticity and conductivity, and conversely, in humid surroundings it takes up water and swells substantially, which again degrades both its mechanical properties and its conductivity [32]. In a skin-mounted electrode these changes alter the contact and therefore shift the signal baseline. For prolonged wear, the problem comes down to how the water is managed. The most widely used remedy is to add a humectant such as glycerol, which has a low vapor pressure and forms hydrogen bonds with water. Wu et al. combined a glycerol humectant with a Na_2_SO_4_ salting-out treatment in a hydrogel fiber, which retained 79.91% of its initial weight at 43% relative humidity, against 32.78% for an untreated PVA/PAA fiber [57]. The extension of this strategy is the organohydrogel treated in Section 2.3.

#### Natural-Polymer-Based Conductive Gels

Natural and bio-derived polymers, including gelatin, alginate, chitosan, cellulose derivatives, silk fibroin, casein/caseinate, and gellan gum, can serve as primary matrices or functional components in conductive gels. Their hydroxyl, carboxyl, amino, and other functional groups enable physical association, ionic coordination, and covalent crosslinking, while also providing sites for adhesion and interaction with other polymer or conductive phases. For example, alginate can form reversible ionic junctions with multivalent cations, whereas gelatin can form physical networks and can be further stabilized through covalent crosslinking. These features can support soft, biologically compatible interfaces, but the presence of a natural polymer does not itself imply high electrical conductivity.

The structural or interfacial role of the biopolymer should therefore be distinguished from the origin of charge transport. In some systems, fixed ionic groups and coordinated or mobile ions within the biopolymer network contribute directly to ionic transport; in others, conductivity is mainly supplied by dissolved salts, conducting polymers, or conductive fillers. In the cellulose-nanofiber/graphene system discussed above, for example, cellulose nanofibers primarily assist graphene dispersion, whereas the electronic pathway is provided by the graphene network [55]. Tordi et al. reported alginate/gelatin organohydrogels in which multivalent-cation crosslinking of alginate enabled tunable ionic conductivity and multiresponsive sensing, including strain sensing with a gauge factor above 1.6 [58]. In a PAAm/casein hydrogel, casein contributed toughness and adhesion, while LiCl provided mobile ionic carriers; the resulting conductivity was 7.53 S∙m^−1^ [59].

Natural polymers can also function as the principal structural matrix in printable sensing gels. Bian et al. developed a 3D-printable fish-gelatin/high-acyl-gellan-gum biogel using transglutaminase crosslinking and ethanol modification. The reported material exhibited a Young’s modulus of 20.7 ± 1.38 kPa, tensile strength of 0.14 ± 0.01 MPa, ultimate strain of 270.54 ± 16.23%, a strain gauge factor of 2.37 ± 0.14, a response latency of 6.1 ± 1.47 ms, and durability over 1000 stretching cycles [60].

Compared with conventional synthetic matrices such as PVA and PAAm, natural-polymer systems can offer biodegradability, abundant chemical functionality, and favorable biological-interface properties, but they also present formulation-specific limitations. Variations in molecular weight and composition can increase batch-to-batch variability, stand-alone conductivity or mechanical robustness may be limited, and hydration, degradation, and processing history can influence long-term stability and reproducibility. Hybrid designs that combine natural polymers with synthetic networks or controlled conductive phases can therefore balance biological and interfacial advantages with mechanical and electrical stability.

### 2.2. Ionogels and Eutectogels

An ionogel is a gel filled with an ionic liquid instead of water. Changing the liquid phase changes both the identity of the charge-carrying ions and the environment in which they move, so conductivity, mechanical properties, and environmental stability all differ from those of a hydrogel.

An ionic liquid is a salt that exists in the liquid state at room temperature. Ordinary table salt is a solid because its small, symmetric ions pack tightly into a crystal lattice; an ionic liquid instead uses large, asymmetric organic ions so that no such lattice can form. The result is a medium composed entirely of ions that nonetheless remains liquid at room temperature. The cations frequently used in wearable gels are of the imidazolium family, and the cases treated in this section likewise use 1-ethyl-3-methylimidazolium ([EMIM]^+^) salts [46]. Because the liquid phase is itself the electrolyte, no water is required, and its negligible vapor pressure means the gel does not dry out.

Fabrication is generally carried out by polymerizing directly within the ionic liquid. Monomer and initiator are dissolved in the ionic liquid and polymerized thermally or by ultraviolet light, so the ionic liquid is trapped within the network as it forms. Unlike a hydrogel, where the gel is made first and the liquid phase introduced afterwards, here the network grows inside the liquid phase. Zhu et al. produced a double network in a single ultraviolet step, obtaining a conductivity on the order of 10^−4^ S∙cm^−1^ and a tensile strength of 2.04 MPa at 60 wt% of the ionic liquid [EMIM][OAc] [46].

This reveals the central constraint of ionogels. The ionic liquid acts as a plasticizer as well as an electrolyte: it inserts itself between the polymer chains and weakens the interactions among them, so increasing its content raises conductivity and stretchability but lowers strength and modulus. Jiang et al. showed that raising the ionic-liquid content from 50 to 70 wt% increased conductivity while decreasing mechanical strength, and varying the composition broadly gave conductivities of 0.09–2.64 mS∙cm^−1^ and tensile strengths of 0.06–5.30 MPa [47]. Conductivity and strength are thus set together by the ionic-liquid content and the polymer network composition.

A gel using a deep eutectic solvent (DES) in place of an ionic liquid is called a eutectogel. A deep eutectic solvent exploits the fact that mixing a hydrogen-bond acceptor (HBA) and a hydrogen-bond donor (HBD) at a particular molar ratio gives a liquid far below the melting point of either component. When an acceptor such as choline chloride is mixed with a donor such as ethylene glycol, glycerol, or urea, the two components hydrogen-bond to each other and cannot form their original crystal structures, so the mixture remains liquid [61]. Whereas the synthesis of an ionic liquid is complex and costly, a deep eutectic solvent can be prepared simply by mixing the two components at a given molar ratio and heating them [61]. Zhang et al. polymerized acrylamide together with a peptide crosslinker in a choline chloride/ethylene glycol eutectic solvent, obtaining a conductivity of 3.82 mS∙cm^−1^; the gel lost only 1.4% of its mass after 30 days under ambient conditions [41] (Figure 2c).

In summary, the advantages of ionogels and eutectogels are concentrated in their resistance to drying, which favors devices requiring prolonged wear or long-term storage. The ionic conductivities of the representative ionogels treated in this section were generally lower than those of the hydrogel examples. This reflects not only the relatively high viscosity of ionic liquids but also ion association and polymer–ion interactions, which can restrict carrier mobility. Conductivity depends strongly on which ionic liquid is used, how much of it is present, and how the polymer network is built. Ionogels are therefore not inherently less conductive than hydrogels. To illustrate the liquid-phase effect quantitatively, representative neat DESs at 298 K show large composition-dependent differences: choline chloride/ethylene glycol (1:2) has a viscosity of 36 cP and a conductivity of 7.61 mS∙cm^−1^, compared with 376 cP and 1.05 mS∙cm^−1^ for choline chloride/glycerol (1:2) and 632 cP and 0.75 mS∙cm^−1^ for choline chloride/urea (1:2) [61]. These values describe the neat DES liquid phases rather than polymerized eutectogels, but they illustrate how viscous resistance can strongly constrain ion transport. Accordingly, these liquid-phase values should be regarded as descriptors of the starting solvent environment rather than as quantitative properties of the final polymerized gel. Ion association can further reduce the fraction of independently mobile charge carriers, while polymer–ion coordination and confinement within the network can impose additional mobility constraints. The dielectric environment also affects electrostatic screening and therefore ion association. Accordingly, the lower conductivities of some ionogel or eutectogel examples than water-rich hydrogel examples should be interpreted as formulation-dependent rather than as an intrinsic hierarchy among gel families.

Low volatility does not, however, guarantee long-term operational stability. Under cyclic deformation, redistribution of the liquid phase and changes in polymer-solvent interactions can alter ion mobility and electrical output. Hydrophilic ionic liquids and DESs can also exchange water with humid air, sweat, saline media, or other biological fluids, changing viscosity, ion concentration, swelling, and polymer-ion interactions; leaching or ion exchange may introduce further drift. The 1.4% mass loss after 30 days reported above [41] therefore demonstrates dry-storage solvent retention, but does not by itself establish stability under repeated deformation or physiological-fluid exposure. As a separate device-level example, the choline chloride/ethylene glycol-based eutectogel discussed in Section 3.2 retained its state and mass for at least one week at 40% relative humidity and maintained stable capacitance over 10,000 loading cycles [62]. These results demonstrate promising retention and cyclic durability under the tested conditions, while long-term performance under sweat or other relevant biological fluids remains application-specific.

### 2.3. Organogels and Organohydrogels

An organogel is a gel in which water is replaced by an organic solvent. If the ionogel represents excluding water altogether, the organogel is closer to a compromise that retains hydrogel-like properties while slowing down drying. In practice, most systems reported for wearable sensors are organohydrogels, in which water is mixed with the organic solvent rather than removed: the organic component prevents evaporation while the remaining water maintains ion dissociation.

The solvents commonly used are glycerol and ethylene glycol. The underlying mechanism is hydrogen bonding: glycerol hydrogen-bonds with water and holds it within the network [32]. A single glycerol molecule carries three hydroxyl groups and can therefore bond to several water molecules at once.

There are two preparation routes: the solvent-displacement route, in which a hydrogel is first formed in water and then immersed in an organic solvent so that the solvent is exchanged by diffusion, and the direct-synthesis route, in which polymerization is carried out in the organic-aqueous mixture from the outset [63,64]. The displacement route preserves a network structure optimized in water but diffusion takes a long time, whereas direct synthesis gives a uniform composition but must account for the effect of the organic solvent on polymerization. As an example of the displacement route, Tang et al. first polymerized a PVA/PAA hydrogel containing LiCl and then immersed it in a glycerol solution to convert it into an organogel [42]. The full sequence of the route in which the gel is polymerized first and the solvent is then exchanged is shown in Figure 2d.

The methods of introducing conductivity are the same as for hydrogels, but the outcome depends strongly on the solvent. Dissolving a salt is a common route; glycerol, however, is more viscous and has a lower dielectric permittivity than water. Lower dielectric permittivity reduces electrostatic screening between oppositely charged ions and can therefore favor stronger ion pairing, decreasing the fraction of freely mobile charge carriers. At the same time, higher viscosity can reduce the mobility of dissociated ions. Consequently, glycerol-rich systems can exhibit lower ionic conductivity than water-rich hydrogels under otherwise comparable conditions. Where higher total conductivity is required, an electronic pathway can be introduced: Guo et al. combined ionic transport from NaCl with an electronic pathway formed by PMMA@MXene conductive spheres, achieving 14.82 S∙m^−1^ [49]. At 25 °C, glycerol has a viscosity of approximately 934 cP and a relative dielectric permittivity of about 42.5 [65]. For comparison, ethylene glycol and DMSO are much less viscous, with reported viscosities of approximately 25.66 and 1.996 cP and dielectric constants of about 37 and 46.7, respectively [66]. These numerical values characterize the neat liquid solvents and should not be interpreted as the effective viscosity or dielectric properties of the final organogel or organohydrogel after network formation. Once the solvents are incorporated into a polymer network, polymer-solvent and polymer-ion interactions, network confinement, and the water-to-organic-solvent ratio can further alter ion mobility and the resulting electrical behavior. These values show that solvent substitution changes both viscous resistance to ion motion and electrostatic screening, although conductivity remains dependent on the full formulation, including salt concentration and polymer-ion interactions.

The main reason for using this platform—its resistance to drying—is seen most clearly in data comparing it side by side with a hydrogel. Liang et al. reported that at 24 °C and 50% relative humidity a hydrogel lost approximately 70% of its mass within 5 h, whereas the mass loss of the double-network organohydrogel plateaued at about 30% over 36 h under the same conditions [67], and Huang et al. confirmed retention above 90% after 7 days at 20 °C [64]. Drying is not eliminated, however: in the gel of Guo et al. the mass retained after 7 days at 20 °C and 50% relative humidity was 69.7%, against 39.8% for the glycerol-free, water-only control [49].

Replacing water with an organic solvent creates the advantages and the disadvantages alike. Conductivity falls first. The data of Ji et al. are the clearest: within the same system, raising the glycerol/water ratio from 0:1 to 1:2 reduced the conductivity from 0.454 to 0.124 S∙m^−1^ [48]. Adding more organic solvent means less drying but less conduction—the same structure as in Section 2.2, where the ionic-liquid content and the network composition set conductivity and strength together. This within-formulation decrease is consistent with the combined effects of higher viscous resistance to ion motion and altered dielectric screening as the glycerol fraction increases.

Similar caution applies to organogels and organohydrogels. Glycerol or other low-volatility solvents can reduce evaporation, but repeated deformation and exposure to sweat or biological fluids can still alter solvent and ion distributions, induce water uptake or solvent exchange, and change conductivity, adhesion, and mechanical response. For example, the glycerol/NaCl/tannic-acid organohydrogel nanofilm discussed in Section 4.1 retained 96.3% of its mass after 200 days at room temperature and maintained stable resistance over 2500 stretching cycles; after 20 min of running, skin-contact impedance recovered during rest, and the electrode maintained an average ECG signal-to-noise ratio above 32.2 dB from days 2 to 9 [68]. This example indicates that solvent-retaining formulations can support prolonged operation, but stability remains dependent on the complete network, interface, and device architecture rather than on solvent retention alone.

Solvent selection is also constrained by the conditions of biological contact. Glycerol is widely used because of its comparatively favorable compatibility profile, whereas ethylene glycol (EG) requires greater caution. EG is substantially more hazardous after oral exposure than after dermal exposure, for which absorption is comparatively limited; nevertheless, prolonged-contact formulations should minimize leakage and evaluate local irritation and exposure to the complete formulation [69]. Dimethyl sulfoxide (DMSO) presents a different consideration: it readily penetrates biological membranes and is widely used as a skin-penetration enhancer [70]. Therefore, DMSO-containing gels should be assessed not only for the solvent itself but also for the potential transdermal exposure to co-present residual monomers, ions, additives, or degradation products. For medical devices intended for repeated or prolonged biological contact, such assessment should be risk-based and consider the intended contact site and duration together with relevant biological endpoints under the ISO 10993-1 framework and applicable regulatory guidance [71]. Organic solvents are also prone to oxidation or decomposition, which can affect the conductivity of the resulting gel [32].

## 3. Sensing Principles of Conductive Gel-Based Sensors

Conductive gel-based sensors transduce mechanical deformation, charge migration, interfacial polarization, or chemical interactions induced by external stimuli into measurable electrical signals. The three-dimensional polymer network of the conductive gels can incorporate a liquid medium together with mobile ions or conductive fillers, enabling responses not only to changes in resistance and capacitance but also to temperature, humidity, and biochemical analytes [14,72,73,74,75]. These signal transduction characteristics are governed by the polymer composition [14,40], crosslinking density [76], solvent content [77], conductive components [14,78], and the interfacial structure between the gel and the electrodes [20,73]. Consequently, even a single gel system can exhibit electrical responses to mechanical, thermal, moisture-related, chemical, and biochemical inputs through different transduction mechanisms.

In this section, gel-based sensing systems are organized primarily according to the target stimulus or analyte. Mechanical sensing is further divided according to the transduction mode into (i) resistive transduction and (ii) capacitive and ionotronic transduction. The remaining subsections address (iii) thermal and moisture sensing and (iv) chemical and biochemical sensing.

### 3.1. Mechanical Sensing Based on Resistive Transduction

Mechanical sensing based on resistive transduction measures changes in electrical resistance resulting from tension, compression, or pressure applied to conductive gels, which modifies the internal charge transport pathways within the gels. Conductive gels can establish ion or electron transport pathways within flexible polymer networks, allowing the gel itself to serve simultaneously as a deformable sensing layer and a signal transport pathway [78,79]. Owing to this structural simplicity and straightforward signal acquisition, conductive gels can directly transduce mechanical deformation into measurable resistance changes. Consequently, resistive transduction is widely used in gel-based wearable sensors for monitoring strain, pressure, and human motion.

The resistive responses of conductive gels can be broadly classified into two types according to the dominant charge carriers: (i) ionic conduction, (ii) electronic conduction. Although these mechanisms rely on different charge carriers and transport pathways, both can convert mechanically induced changes in charge-transport pathways and conditions into measurable resistance changes [72,78].

**Figure 3:**
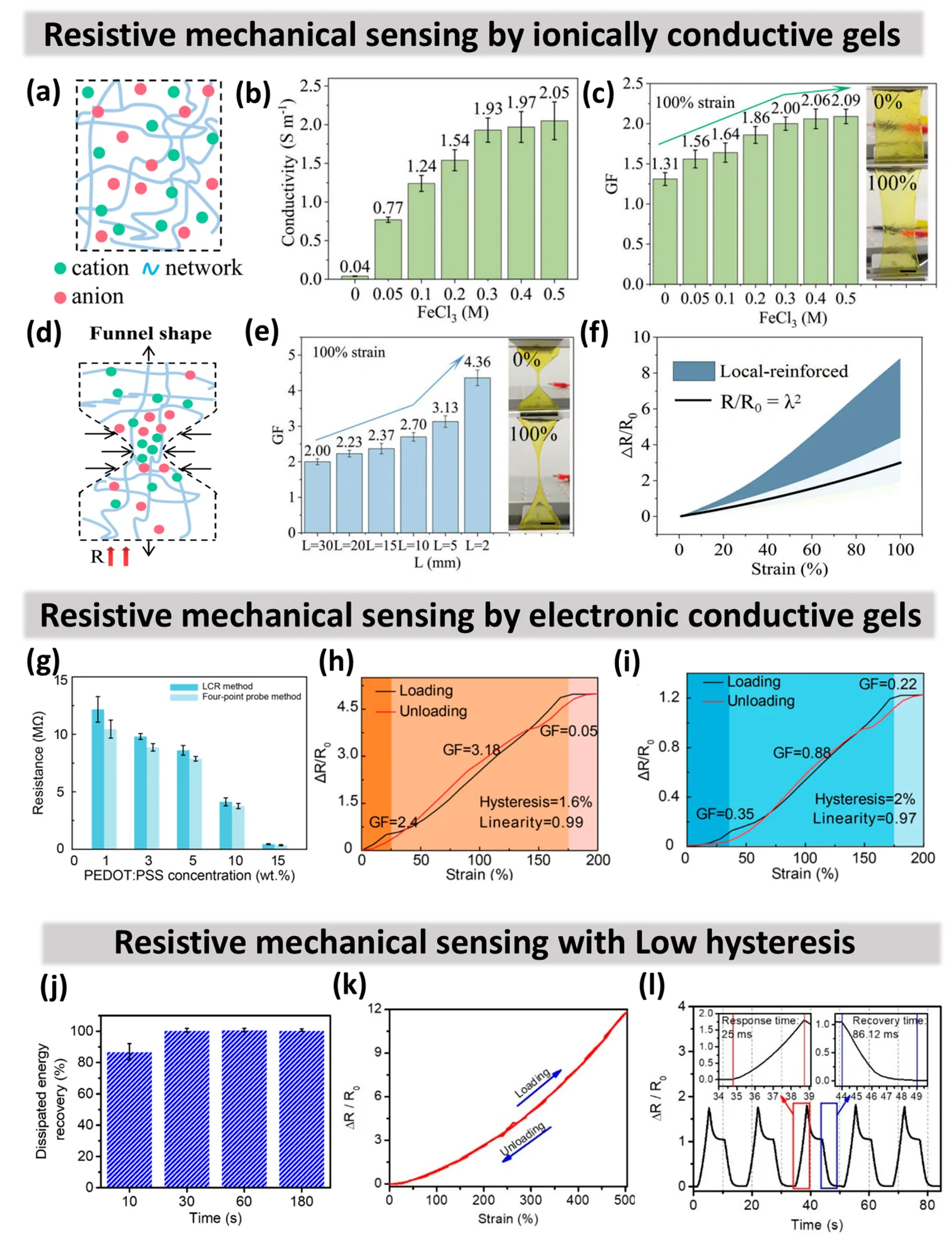
**Resistive mechanical sensing by ionically and electronically conductive gels and a strategy for low-hysteresis sensing.** (**a**–**f**) Regulation of the resistive response of ionically conductive poly(vinyl alcohol) (PVA)/glycerol/FeCl_3_ hydrogels through ionic composition and sensor geometry. (**a**) Schematic illustration of mobile cations and anions within the polymer network. (**b**) Ionic conductivity as a function of FeCl_3_ concentration. (**c**) Gauge factor (GF) at 100% strain as a function of FeCl_3_ concentration. (**d**) Funnel-shaped geometry for concentrating strain within the narrowed sensing region. (**e**) GF at 100% strain as a function of the width (*L*) of the narrowed region. (**f**) Relative resistance response of the locally reinforced funnel-shaped sensor compared with that predicted for uniform deformation. Adapted from Zhao et al. [72]. © 2023 The Authors. Published by Wiley-VCH GmbH under a Creative Commons Attribution (CC BY) license. (**g**–**i**) Regulation of the resistive response of electronically conductive PEDOT:PSS-PVA hydrogels through PEDOT:PSS content. (**g**) Electrical resistance at different PEDOT:PSS concentrations measured using an LCR meter and the four-point probe method. (**h**) Loading-unloading relative resistance response of the hydrogel containing 3 wt% PEDOT:PSS. (**i**) Loading-unloading relative resistance response of the hydrogel containing 15 wt% PEDOT:PSS. Adapted from Cao et al. [78]. © 2023 by the authors. Published by MDPI under a CC BY license. (**j**–**l**) Rapid mechanical recovery and low-hysteresis resistive sensing of a Ca^2+^/SiO_2_-nanoparticle-reinforced hybrid conductive hydrogel. (**j**) Recovery of dissipated energy after different relaxation times. (**k**) Nearly hysteresis-free relative resistance response during loading and unloading up to 500% strain. (**l**) Cyclic relative resistance response and the corresponding response and recovery times. Adapted from Xu et al. [84]. © 2021 by the authors. Published by MDPI under a CC BY 4.0 license.

In ionically conductive gels, charge transport is mediated by cations and anions dissociated from salts or ionic liquids [79,80], or by counterions associated with ionic functional groups immobilized on the polymer chains, which migrate through the liquid medium confined within the polymer network [81]. Accordingly, the resistive sensing behavior of ionically conductive gels can be tailored through both ionic composition and structural design. The concentration of mobile ions influences the baseline ionic conductivity and relative resistance response, while macroscopic sensor geometry and mechanical reinforcement may control the spatial distribution of strain during deformation. For example, Zhao et al. combined a poly(vinyl alcohol) (PVA)/glycerol/FeCl_3_ hydrogel with a funnel-shaped sensing region and porous thermoplastic polyurethane (TPU) reinforcement to localize strain (Figure 3a–f). Increasing the FeCl_3_ concentration raised the conductivity and gauge factor until the response approached a plateau at 0.3 M. Narrowing the sensing region from 30 to 2 mm increased the gauge factor at 100% strain from 2.00 to 4.36, while TPU reinforcement further increased it to 9.21 without compromising linearity over 0–100% strain [72]. This example shows that ionic content controls baseline charge transport, whereas geometry and reinforcement can amplify deformation sensitivity.

In electronically conductive gels, conductive components such as intrinsically conductive polymers, including PANI, PPy, and PEDOT:PSS; metallic nanomaterials, including gold (Au) or silver (Ag) nanoparticles and Ag nanowires (AgNWs); carbon-based materials such as CNTs and graphene; and MXenes form conductive networks within the gel that enable electron transport [14,77,78]. The connectivity of this network is influenced not only by the content of the conductive components [78] but also by their morphology [40], aggregation [82], and interfacial interactions with the polymer matrix [83], thereby affecting the electronic conductivity and resistive response of the gel. For example, Cao et al. incorporated PEDOT:PSS nanofibers into a PVA hydrogel and found that increasing the PEDOT:PSS content from 1 to 15 wt% reduced electrical resistance but also lowered sensitivity over most of the tested strain range (Figure 3g–i) [78]. These results demonstrate that the content of conductive polymers governs both the baseline electrical resistance and the deformation-induced change in network connectivity, highlighting the need to balance electrical conduction and resistive sensitivity.

The output of a resistive sensor is generally expressed as the relative resistance change with respect to the initial resistance (ΔR/R_0_), while the sensitivity of a strain sensor is commonly evaluated using the gauge factor (GF = [ΔR/R_0_]/ε). However, when conductive pathways are progressively disrupted or re-established across different strain ranges [77,78], a single sensor may exhibit different gauge factors depending on the deformation regime. Therefore, although high sensitivity is advantageous for distinguishing small deformations, practical sensor performance is determined not only by sensitivity but also by the working range, linearity, and repeatability [72,78].

In conductive gels, hysteresis during loading and unloading can arise from viscoelastic relaxation of polymer chains [85], interchain friction and slippage [86], dissociation and reformation of dynamic bonds [87], partial damage to brittle networks [52], and interfacial delamination between conductive components and the polymer matrix [88]. Low hysteresis indicates that the mechanical deformation and conductive network changes recover with only a small deviation in the electrical signal, resulting in similar resistance response paths during loading and unloading [88]. This is a key requirement for ensuring signal reproducibility during long-term monitoring and repeated measurements in wearable and attachable devices. Therefore, resistive gel sensors should be designed to achieve low hysteresis and stable cyclic responses along with high sensitivity. Xu et al. demonstrated that the rapid restoration of reversible crosslinks can suppress hysteresis in the resistive response of an ionically conductive hydrogel. The hydrogel comprised a PAAm, hydroxypropyl guar gum (HPG), and acryloyl-grafted chitosan quaternary ammonium salt (acryloyl-grafted CQAS) network, denoted as PHC, containing calcium ions (Ca^2+^) and silica nanoparticles (SiO_2_ NPs). Permanent covalent crosslinks maintained structural integrity, while hydrogen bonds among the polymers, coordination interactions between Ca^2+^ and acryloyl-grafted CQAS, and hydrogen-bonding interactions between SiO_2_ NPs and the polymers served as reversible interactions. These interactions partially dissociated during deformation to dissipate energy and rapidly reformed after unloading. The hydrogel therefore recovered 80% and 100% of its dissipated energy within 10 and 30 s, respectively (Figure 3j). Its loading and unloading relative resistance curves were nearly coincident over 0–500% strain, with response and recovery times of 25.00 and 86.12 ms, respectively (Figure 3k,l) [84]. These results demonstrate that combining a stable covalent network with rapidly reforming reversible interactions can promote rapid mechanical recovery and minimize hysteresis in the resistive response.

Resistive sensing is attractive for motion monitoring with straightforward readout, but its sensitivity and operating range must be considered together with the reversibility of deformation-induced changes in charge transport [78]. In electronically conductive gels, network rearrangement and slippage between conductive and polymer phases can contribute to hysteresis and time-dependent resistance changes [85,88]. In ionically conductive gels, changes in solvent content and ion mobility can shift baseline resistance and complicate the interpretation of deformation-induced signals [89]. Viscoelastic relaxation and network recovery can also affect the temporal electrical response in both types [84,85]. Accordingly, cyclic stability and waveform reproducibility should be evaluated at the strain amplitude and loading rate relevant to the target application [90]. Comparisons before and after cycling under comparable measurement conditions can address aspects such as sensitivity retention, changes in sensing hysteresis, and shifts in unloaded baseline resistance.

### 3.2. Mechanical Sensing Based on Capacitive and Ionotronic Transduction

In capacitive and ionotronic mechanical sensing, conductive gels combine electronic or ionic conductivity with a low modulus, high stretchability, and mechanical conformability, allowing them to serve as soft electrodes or ion-conducting layers in deformable capacitors [79,91]. These properties enable conductive gel-based sensors to be integrated with the skin or clothing for wearable and attachable healthcare devices that detect mechanical stimuli, including pressure, contact, tensile deformation, and body motion, through capacitance changes [73,91]. Depending on the origin of the measured capacitance, conductive gel-based sensors can operate through conventional capacitive sensing or ionotronic sensing based on an electrical double layer (EDL).

**Figure 4:**
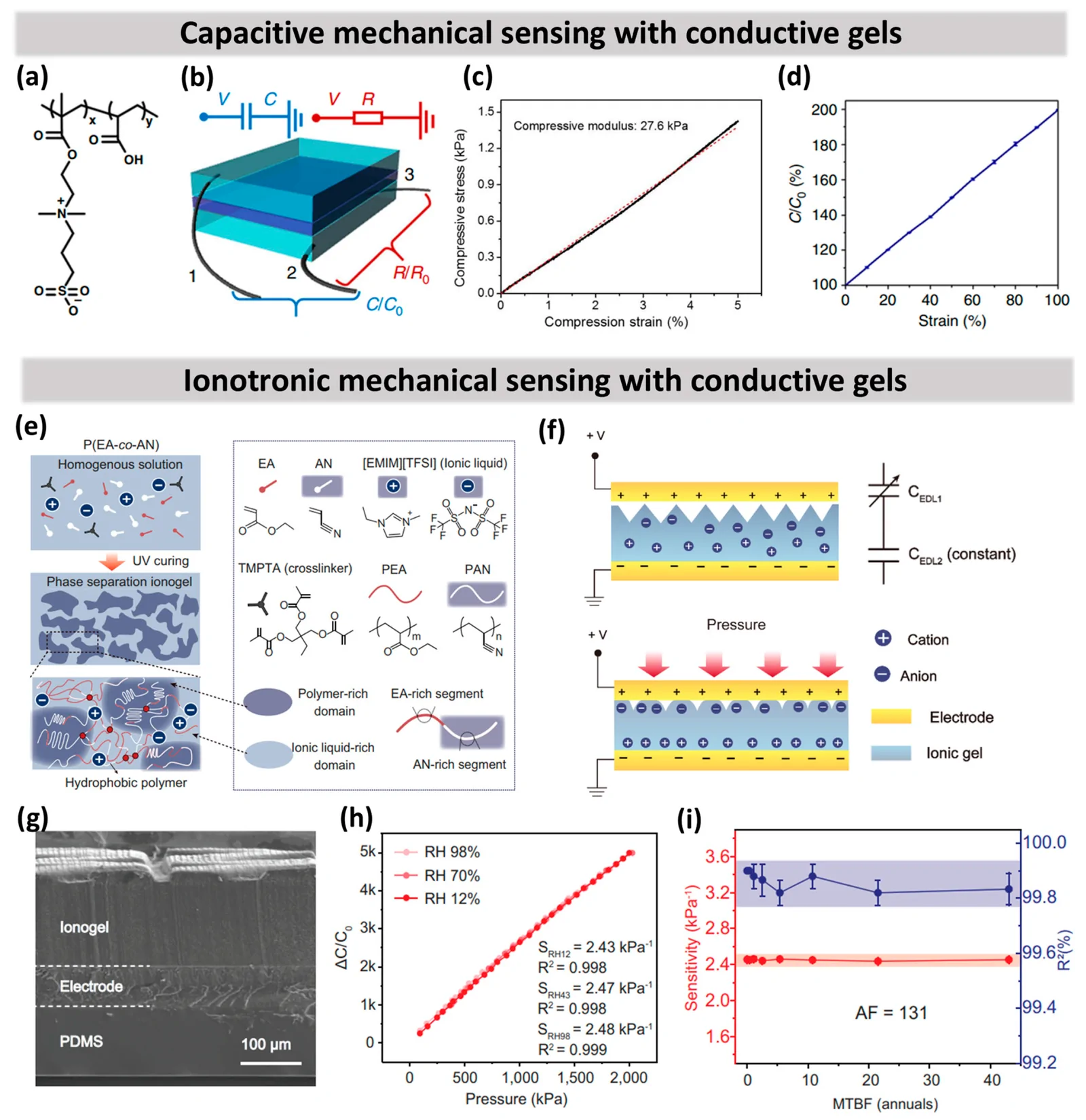
**Representative capacitive and ionotronic sensing configurations for mechanical sensing based on conductive gels.** (**a**–**d**) Capacitive strain sensing using a supramolecular poly(acrylic acid-*co*-3-dimethyl(methacryloyloxyethyl)ammonium propane sulfonate) (PAA-*co*-DMAPS) hydrogel. (**a**) Chemical structure of PAA-*co*-DMAPS. (**b**) Device architecture and equivalent circuits for capacitive and resistive readout. (**c**) Compressive stress–strain response. (**d**) Normalized capacitance as a function of tensile strain. Adapted from Lei and Wu [92]. © The Author(s) 2018. Published by Springer Nature under a CC BY 4.0 license. (**e**–**i**) Humidity-insensitive ionotronic pressure sensing using a phase-separated poly(ethyl acrylate-*co*-acrylonitrile) [P(EA-*co*-AN)] ionogel. (**e**) Ultraviolet-induced phase separation of the ionogel. (**f**) Electric-double-layer-based pressure-sensing mechanism. (**g**) Cross-sectional image of the multilayer interface after cyclic rubbing. (**h**) Capacitance-pressure responses at different relative humidity levels. (**i**) Sensitivity and linearity during accelerated aging. Adapted from Shi et al. [93]. © The Author(s) 2024. Published by Oxford University Press on behalf of China Science Publishing & Media Ltd under a CC BY 4.0 license.

A conventional capacitive sensor generally has a sandwich configuration in which a dielectric layer is positioned between two electrodes, and its capacitance can be expressed as *C* = *ε*_0_*ε_r_A*/*d*, where *ε*_0_ and *ε_r_* are the vacuum permittivity and relative permittivity of the dielectric, respectively, *A* is the overlapping electrode area, and *d* is the separation between the electrodes. Applied pressure or tensile deformation changes the overlapping area and electrode separation, producing a capacitance response. Conductive gels can function as compliant electrodes that retain electrical connectivity during repeated stretching, bending, and compression [79,80]. Accordingly, the design of conductive gel-based capacitive sensors should consider not only the ionic or electronic conductivity of the gel electrodes but also their stretchability, compressive compliance, elastic recovery, and structural and electrical stability under repeated deformation [77,80]. Lei and Wu introduced an ionically conductive hydrogel based on a dynamic supramolecular network that maintained reversible capacitive responses under repeated deformation and enabled capacitance recovery even after mechanical damage to the hydrogel electrodes. They prepared a supramolecular polyelectrolyte hydrogel, denoted as PAA-co-DMAPS, by copolymerizing acrylic acid and 3-dimethyl (methacryloyloxyethyl) ammonium propane sulfonate (DMAPS) (chemical structure shown in Figure 4a). The hydrogel was equilibrated in a 4 M NaCl solution, and a dielectric elastomer was sandwiched between two hydrogel electrode layers to construct a skin-mimetic capacitive sensor (Figure 4b). The dynamic network exhibited a compressive modulus of 27.6 kPa, elastic recovery after compression, high stretchability, and autonomous self-healing (Figure 4c). Consequently, the sensor maintained reversible capacitance responses during 100 tensile cycles between 0 and 100% strain (Figure 4d). After the hydrogel electrodes were cut and brought back into contact, the capacitance recovered within 20 min with approximately 0.9% drift [92].

In ionotronic mechanical sensing, an ion-conducting gel containing mobile ions is brought into contact with electronically conductive electrodes and functions as the ion-conducting component of the capacitor [20,73]. Upon application of a voltage, cations and anions migrate toward oppositely charged electrode surfaces and form EDLs at the gel–electrode interfaces. When an external pressure increases the real contact area between the gel and electrode, the effective interfacial area available for EDL formation also increases, resulting in a pressure-dependent change in interfacial capacitance [73]. The high areal capacitance generated by the EDL facilitates the detection of small interfacial changes and reduces the relative influence of parasitic capacitance and surrounding electrical interference [20].

In ion-conducting-gel-based ionotronic mechanical sensors, microprotrusions or wrinkles introduced onto the gel surface in contact with the electrode can amplify pressure-induced changes in the real gel–electrode contact area [94,95]. In addition, hierarchical [94] or graded structures [73] with different heights or deformation characteristics can promote the sequential deformation and contact of individual structural elements, thereby providing a means to tune the sensor sensitivity and sensing range. For example, Shi et al. developed an ionotronic pressure-sensor array that maintained a linear response over a wide pressure range while remaining insensitive to humidity variations by using a hydrophobic ionogel patterned with a pillar-like microstructure having gradients in both width and height. They prepared a phase-separated, bicontinuous poly(ethyl acrylate-co-acrylonitrile) [P(EA-co-AN)] ionogel by copolymerizing ethyl acrylate (EA) and acrylonitrile (AN) in the ionic liquid 1-ethyl-3-methylimidazolium bis(trifluoromethylsulfonyl)imide ([EMIM][TFSI]) (Figure 4e). The soft ionic-liquid-rich domain provided ionic conductivity and ductility, whereas the stiff polymer-rich domain imparted a high Young’s modulus and mechanical strength; the hydrophobicity of both phases contributed to stable mechanical and electrical properties under highly humid conditions. Sandwiching the ionogel between two flexible polyimide-copper (PI-Cu) electrodes formed nanoscale electric double layers (EDLs) at the electrode-ionogel interfaces (Figure 4f,g). Pressure-induced changes in interfacial contact area altered the EDL capacitance, while the graded pillar-like microstructure regulated contact-area evolution to produce a linear response. The high Young’s modulus of 107 MPa extended the linear sensing range to 0–2.0 MPa. Consequently, the sensor exhibited sensitivities of 2.43, 2.47, and 2.48 kPa^−1^ at relative humidity (RH) levels of 12%, 70%, and 98%, respectively, while maintaining *R*^2^ > 0.998 under all three conditions (Figure 4h,i) [93].

The outputs of capacitive and ionotronic mechanical sensors are generally expressed as the relative capacitance change (ΔC/C_0_), and their performance can be evaluated in terms of sensitivity, linear operating range, detection limit, response and recovery times, hysteresis, and cyclic stability [73,94]. In conventional capacitive sensing, reproducible changes in electrode separation and overlap can support repeatable strain measurements, provided that electrode conductivity and dielectric properties remain stable [92]. However, dielectric creep and interlayer slippage can introduce hysteresis and delay signal recovery [90]. In EDL-based ionotronic sensing, pressure-induced changes in the real contact area can generate large capacitance responses, while sensitivity and operating range depend on microstructure deformation and the progression toward full contact. Because EDL capacitance is frequency-dependent, the excitation frequency also needs to be specified alongside sensitivity and response-time measurements [73].

During prolonged operation, repeated deformation and solvent exchange with the surrounding environment can progressively alter sensor geometry, interfacial contact, and the distribution and mobility of ions, potentially changing both the baseline capacitance and the stimulus-dependent response [77,89]. The loss of water or other solvents can cause gel shrinkage, reduced conductivity, and changes in device geometry, whereas solvent uptake and swelling can alter electrode separation and interfacial contact [74]. Ion leakage and redistribution, creep of the polymer network, damage to microstructures under cyclic loading, and instability of the interfaces between device layers can also cause baseline shifts and signal drift [14,85,88,89].

**Figure 5:**
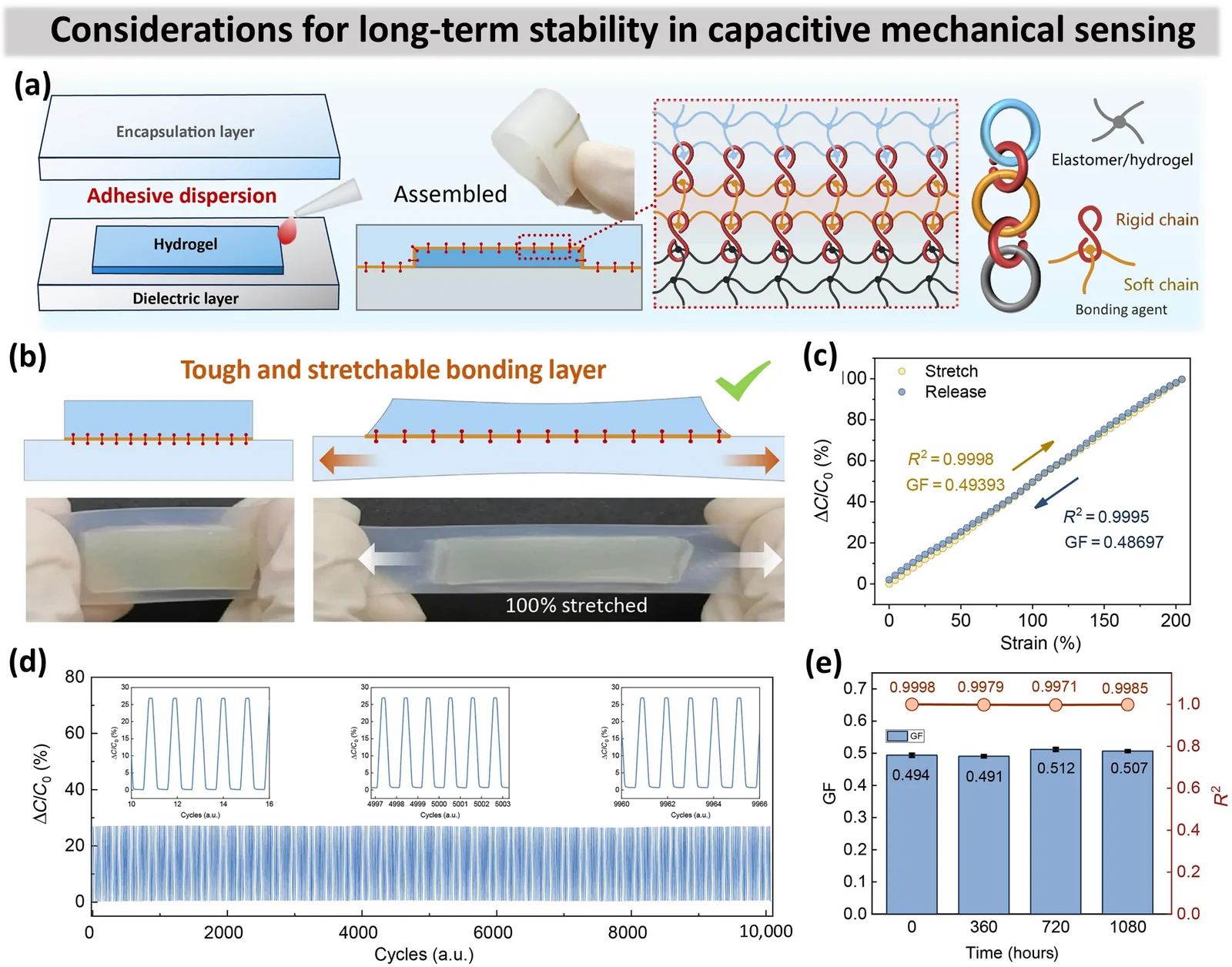
**Interfacial coordination and long-term stability in conductive gel-based capacitive mechanical sensing.** (**a**–**e**) Tough interfacial bonding, capacitive response, and operational stability of a multilayer hydrogel-elastomer sensor. (**a**) Assembly of the hydrogel, dielectric, and encapsulation layers using a rigid-flexible dual-network adhesive that connects the adjoining polymer networks through molecular-level hooking and interlocking. (**b**) Tough and stretchable bonding layer maintaining interfacial integrity under 100% tensile strain. (**c**) Relative capacitance change (Δ*C*/*C*_0_) during stretching and release up to 204% strain, showing closely matched linear responses. (**d**) Cyclic capacitance response during 10,000 loading–unloading cycles at 50% strain. (**e**) Gauge factor (GF) and linearity (*R*^2^) measured over 1080 h. Adapted from He et al. [90]. © 2026 The Authors, some rights reserved; exclusive licensee American Association for the Advancement of Science. Distributed under a Creative Commons Attribution License 4.0 (CC BY).

These effects are particularly relevant to wearable and attachable sensors, which can experience repeated body deformation and changes in environmental conditions during use [77,89]. Therefore, to ensure stable capacitive and ionotronic sensing over extended operation, sensor design should consider factors such as the use of conductive gels with high solvent retention [77], networks that suppress swelling and freezing [96], stable encapsulation strategies [97], and crosslinking structures with self-healing capability [78,87]. For example, He et al. fabricated a capacitive strain sensor by robustly integrating lithium chloride (LiCl)- and glycerol-modified sodium hyaluronate-PAAm (SH-PAAm) hydrogel electrodes, a 3M Very High Bond (VHB) polyacrylate dielectric layer, and Ecoflex/VHB encapsulation layers using a stretchable dual-network adhesive as shown in Figure 5a. The robust interfacial bonding suppressed interlayer slippage and enhanced the elastic recovery of the multilayer structure (Figure 5b), while the LiCl-glycerol composition and encapsulation maintained interfacial hydration and restricted water migration and evaporation. Consequently, the sensor exhibited a linear capacitive response over a strain range of 0–204%, maintained signal variations within ±2% after 10,000 loading–unloading cycles at 50% strain, and showed negligible performance changes after 1080 h under ambient conditions [90], as shown in Figure 5c–e.

Beyond robust interfacial bonding and encapsulation-based stabilization, dynamically reversible crosslinks can be employed to impart intrinsic self-healing capability to the gel. Liu et al. demonstrated a capacitive pressure sensor with stable capacitance responses under repeated loading by employing an ionically conductive eutectogel featuring a fully physically crosslinked network based on hydroxyapatite (HAp)-mediated coordination bonds and tannic acid (TA)-mediated hydrogen bonds. In this eutectogel, HAp formed strong coordination bonds with poly(N-acryloyl-2-glycine) (PACG) chains, while TA contributed hydrogen-bonding interactions, and these dynamically reversible physical interactions enabled reconstruction of the disrupted gel network. The optimized ATH_6_ eutectogel achieved a self-healing efficiency of 96.5% after 3 h at room temperature and recovered its original shape without fracture after repeated compression at 80% strain. The use of a choline chloride/ethylene glycol-based deep eutectic solvent also allowed the eutectogel to retain its original state and mass for at least one week at room temperature and 40% relative humidity. A sensor constructed by sandwiching a dielectric film between two ATH_6_ eutectogel electrodes converted pressure-induced variations in electrode separation into capacitance changes and maintained stable capacitance responses over 10,000 loading cycles at 70 Pa [62].

The performance of conductive gel-based mechanical sensors depends on the combined effects of the transduction mechanism, gel composition, structural design, and interfacial stability. Mechanism selection therefore needs to consider sensitivity together with hysteresis, linearity, response and recovery times, baseline drift, repeatability, working range, and mechanical durability. A high gauge factor or sensitivity alone does not establish superior sensing performance, because practical performance also depends on whether the output is reproducible, follows changes in the target signal, and remains stable during repeated deformation and prolonged use [90,98]. When comparing resistive, capacitive, and ionotronic sensors, the reported test conditions need to be considered together with the performance requirements of the target application [99]. For example, joint-motion monitoring requires an adequate strain range, low hysteresis, and reproducible cyclic responses [90], whereas arterial-pulse monitoring requires sufficient pressure resolution and response speed to preserve waveform features [100]. Under sustained loading, baseline drift and stability over the intended monitoring period become particularly important [98]. Meeting these signal-resolution and temporal requirements during prolonged monitoring also requires consideration of the energy consumed by the readout system. Resistive readout dissipates power through conduction during electrical excitation, while capacitive and ionotronic readouts involve charging and discharging of the sensing element. Power requirements therefore need to be evaluated for the complete measurement system, accounting for factors such as excitation conditions, sampling rate, duty cycle, and supporting circuitry [73,101]. Such evaluation supports the joint selection of the sensing mechanism and readout configuration to meet the performance requirements and intended operating duration of the wearable or attachable application.

### 3.3. Thermal and Moisture-Responsive Sensing

Thermal and moisture-responsive sensing in conductive gels originates from changes in charge transport and solvent state caused by variations in temperature or environmental water content. Because conductive gels confine mobile ions, electronically conductive components, and liquid media within three-dimensional polymer networks, thermal or moisture stimuli can modify their resistance, impedance, conductivity, or generated voltage [74,77,102,103]. These responses allow soft and conformal devices to detect body and ambient temperature, humidity variations during respiration, and moisture released from the skin.

Temperature sensing generally relies on the thermal dependence of ionic or electronic conduction. In ionically conductive gels, temperature alters ion dissociation and mobility, thereby changing ionic conductivity and electrical resistance [102]. In electronically conductive gels, the resistance response can arise from temperature-dependent charge transport through conductive polymers or interconnected filler networks [77]. Temperature-induced swelling, contraction, or phase transition of a thermoresponsive polymer network can further rearrange the solvent distribution and conductive pathways, thereby amplifying the electrical response [104].

**Figure 6:**
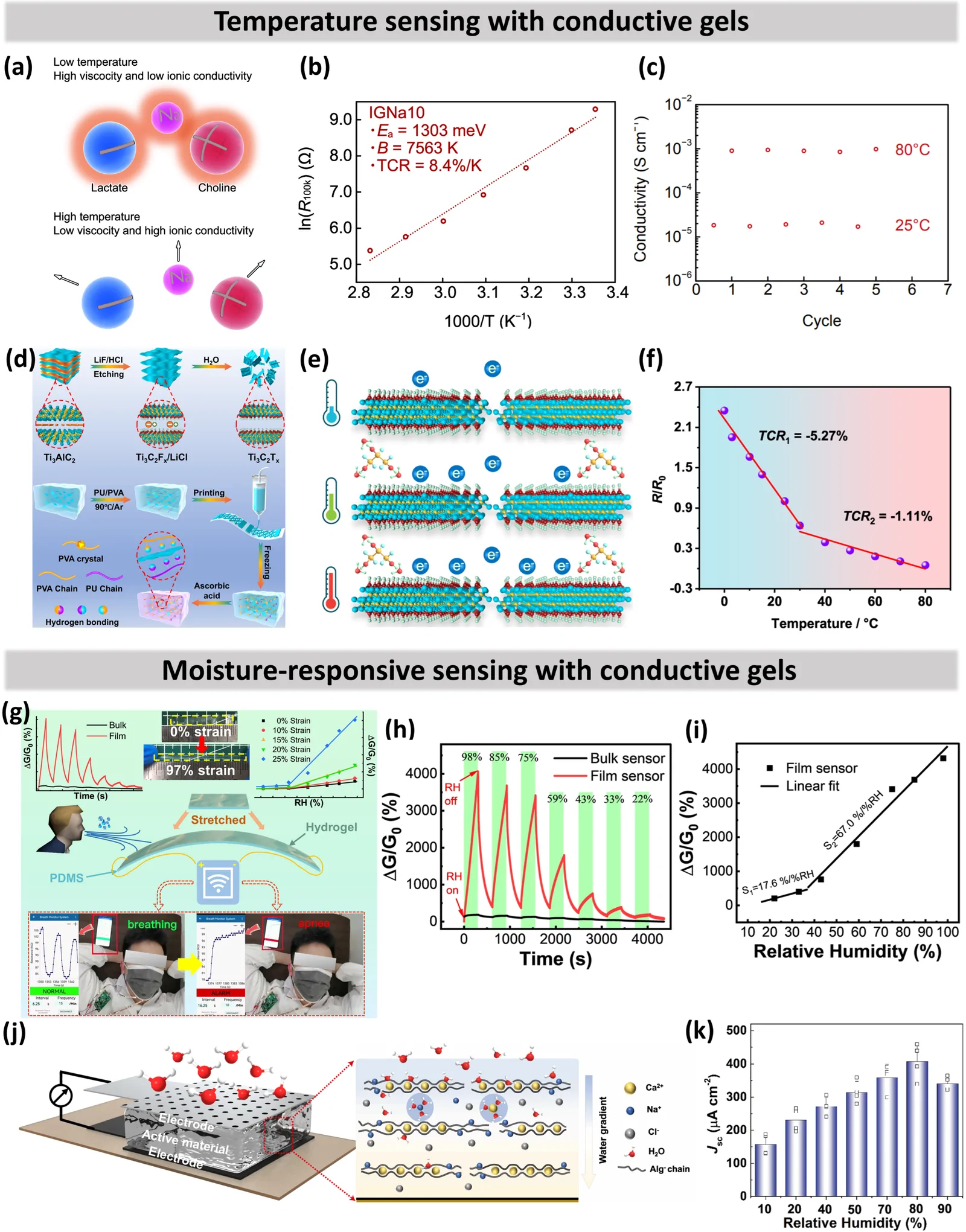
**Representative temperature- and moisture-responsive sensing systems based on conductive gels.** (**a**–**c**) Temperature sensing using a biodegradable PVA/sodium-doped choline lactate ionic gel (IGNa10). (**a**) Temperature-dependent ionic-conduction mechanism. (**b**) Arrhenius plot of resistance. (**c**) Cyclic conductivity at low and high temperatures. Adapted from Yamada and Honda [102]. © 2025 The Authors. Published by American Chemical Society under a CC BY 4.0 license. (**d**–**f**) Temperature sensing using a 3D-printed Ti_3_C_2_T_x_ MXene-bonded polyurethane/poly(vinyl alcohol) (PU/PVA) hydrogel. (**d**) Preparation and printing of the hydrogel. (**e**) Temperature-dependent electron-transport mechanism. (**f**) Normalized resistance (*R*/*R*_0_) as a function of temperature. Adapted from Liu et al. [77]. © The Author(s) 2022. Published by Springer Nature under a CC BY 4.0 license. (**g**–**i**) Humidity sensing using a stretchable polyacrylamide (PAAm)/carrageenan double-network hydrogel film. (**g**) Sensor concept and wireless respiration monitoring. (**h**) Responses of the bulk and film sensors to repeated relative humidity (RH) changes. (**i**) Relative conductance change as a function of RH. Adapted from Liang et al. [74]. © The Author(s) 2022. Published by Springer Nature under a CC BY 4.0 license. (**j**,**k**) Moisture-responsive electricity generation using a poly(vinyl alcohol)/sodium alginate (PVA/AlgNa) hydrogel. (**j**) Device structure and water-gradient formation. (**k**) Short-circuit current density as a function of RH. Adapted from Yang et al. [103]. © The Author(s) 2024. Published by Springer Nature under a CC BY 4.0 license.

Accordingly, the design of conductive gel-based temperature sensors should consider the identity and concentration of charge carriers [102], solvent composition [89], polymer–ion interactions [105], crosslinking structure [104], and thermal stability of the conductive phase [77,97]. Their performance should be evaluated in terms of sensitivity, linear operating range, response and recovery times, thermal hysteresis, and repeatability during heating and cooling cycles [97]. For example, Yamada and Honda enhanced the temperature response of a biodegradable choline lactate ionic gel by adding sodium ions and dispersing the optimized ionic liquid in PVA. Heating promoted ion dissociation and reduced viscosity, thereby decreasing impedance (Figure 6a–c). The resulting gel exhibited an absolute temperature coefficient of resistance (|TCR|) of 8.4% K^−1^ at 300 K and a linear Arrhenius relationship from 25 to 80 °C. Polymer encapsulation reduced moisture interference and enabled stable responses over five temperature cycles [102]. This example shows that carrier chemistry can improve sensitivity, but moisture control and cyclic stability remain necessary for practical temperature sensing.

In another case, Liu et al. demonstrated resistive temperature sensing based on temperature-dependent electronic transport using a direct-ink-written polyurethane (PU)/PVA hydrogel containing Ti_3_C_2_T_x_ MXene flakes and glycerol (Figure 6d–f). As the temperature increased, thermal excitation promoted electron transitions and tunneling transport within the surface-functionalized Ti_3_C_2_T_x_ network with a narrow bandgap, thereby increasing the charge-carrier concentration and electrical conductivity. The Ti_3_C_2_T_x_ flakes also enhanced the thermal conductivity and thermal diffusivity of the hydrogel, contributing to its rapid temperature response, while glycerol improved solvent retention and stabilized the PVA microcrystalline domains during heating, thereby extending the temperature-sensing range. Accordingly, the printed sensor exhibited TCR values of −5.27% °C^−1^ over 0–30 °C and −1.11% °C^−1^ over 30–80 °C [77]. Together, these studies illustrate distinct routes to temperature sensing. The ionic gel relies primarily on changes in ion dissociation and mobility with temperature, whereas the MXene composite relies on thermally activated electronic transport. In both cases, practical performance depends not only on a high TCR but also on calibration across the intended temperature range and maintenance of the solvent state and conductive pathways.

Moisture-responsive sensing is based on electrical changes produced by the uptake and release of water. Water absorption can alter the solvent content, swelling state, ionic concentration, and charge-transport environment of a conductive gel, leading to measurable variations in resistance, impedance, or conductivity [74]. In ionically conductive systems, absorbed water can promote ion dissociation and migration. For instance, Liang et al. fabricated a micrometer-thick PAAm/carrageenan double-network hydrogel film for humidity sensing based on water adsorption and desorption, as shown in Figure 6g. As the relative humidity (RH) increased, the hydrophilic hydroxyl and amide groups promoted water uptake by the hydrogel. The absorbed water increased salt dissolution and ionic mobility, while hydrogel swelling reduced the obstruction of ion transport by the polymer network, thereby increasing the conductance. When the RH decreased, water desorption returned the conductance toward its initial state. Because the thin-film geometry increased the specific surface area and shortened the water-diffusion distance, the hydrogel-film sensor with a thickness of 54.61 μm exhibited substantially amplified and reversible conductance changes compared with the bulk hydrogel sensor with a thickness of approximately 1 cm. These changes were observed during repeated RH cycling between 11% and higher levels ranging from 22% to 98% (Figure 6h). The sensor response was expressed as Δ*G*/*G*_0_, where *G*_0_ was the initial conductance at 11% RH. The 54.61-μm-thick hydrogel film exhibited an initial conductance of 2.75 × 10^−8^ S at 11% RH and a relative conductance response of approximately 4.3 × 10^3^% at 98% RH (Figure 6i). The stretchable hydrogel-film sensor was integrated into a Bluetooth-enabled mask for real-time monitoring of breathing patterns and respiratory interruption (bottom of Figure 6g) [74].

When moisture uptake occurs unevenly across the gel, the resulting ionic concentration gradient may also drive directional ion transport and generate a voltage without continuous external bias. For example, Yang et al. presented a moisture-electric generator using a PVA-sodium alginate (AlgNa)-based supramolecular hydrogel as the active layer. Atmospheric moisture entered through the porous aluminum top electrode but was blocked by the PI bottom substrate, thereby establishing a sustained water gradient across the thickness of the hydrogel. The absorbed water promoted the dissociation and directional migration of Ca^2+^, Na^+^, and Cl^−^ within the AlgCa/Na network, and the resulting asymmetric ion distribution generated a voltage and direct current without an external power supply (Figure 6j). As the relative humidity increased from 10% to 80%, the open-circuit voltage increased from approximately 0.8 to 1.3 V, while the short-circuit current density reached approximately 407 μA∙cm^−2^ (Figure 6k) [103]. Therefore, the moisture response can be governed by the hydrophilicity of the polymer, network porosity, crosslinking density, gel thickness, ionic composition, and water-diffusion kinetics. Sensor design should balance rapid water uptake with efficient desorption, while maintaining low hysteresis, sufficient solvent retention, and stable responses over repeated humidification and drying cycles.

Temperature and moisture responses are often coupled because both stimuli influence charge-carrier concentration and mobility, the physical state of the confined solvent, and the dimensions of the polymer network [89]. To mitigate the coupling between temperature and humidity responses, Isano et al. constructed a transparent and breathable thin-film sensor comprising two ionogel sensing channels with different water affinities. A hydrophobic poly(vinylidene fluoride-co-hexafluoropropylene) (P(VDF-HFP))-based ionogel with negligible water uptake was used as a predominantly temperature-sensitive channel, whereas a hydrophilic Pebax-based ionogel responded to both temperature and humidity. The temperature obtained from the hydrophobic ionogel channel was then used to compensate for the temperature-dependent impedance of the hydrophilic channel, enabling the simultaneous and independent determination of temperature and relative humidity. The sensor exhibited a temperature sensitivity of 15.4% °C^−1^ at 25 °C and a humidity sensitivity of 2.0% per percentage point of relative humidity [106]. This example represents how combining ionogels with different water affinities can enable independent temperature and humidity measurements despite their coupled effects on impedance. However, in coupled temperature–humidity sensing, high sensitivity to either stimulus alone does not by itself establish accurate signal separation [74,106]. Therefore, decoupling performance can be assessed using the errors in the individual temperature and humidity estimates across combined temperature–humidity conditions.

In wearable and attachable applications, conductive gel-based sensors should be evaluated under combined temperature, humidity, and deformation conditions representative of their actual operating environments, with residual cross-sensitivity and calibration stability assessed over the intended monitoring period [89,106]. In multimodal sensing, strategies such as employing distinct output parameters [107], spatially or functionally independent sensing elements [106], selectively attenuating interfering responses [89], and using data-driven models to classify or decouple overlapping responses [108] should be considered to distinguish the contributions of temperature, moisture, and mechanical deformation. For example, Georgopoulou et al. developed a three-dimensionally printable double-network granular organogel (DNGOG)-based e-skin that exhibited resistive responses to mechanical deformation, ambient temperature, and humidity. Tensile deformation increased the relative resistance, while decreasing temperature reduced ionic mobility and increased resistance. Humidity was evaluated separately in a controlled chamber, where increasing relative humidity decreased the resistance. Because thermal and mechanical stimuli generated overlapping resistance changes when applied simultaneously, the authors trained a long short-term memory neural network using resistance-time profiles obtained under pressure, heating, combined pressure and heating, and non-contact conditions. The model classified these stimulus conditions with an accuracy of 98%, demonstrating data-driven classification of overlapping thermal and mechanical responses from a single resistive sensing element. The humidity response was excluded from this classification because its drying-dependent response occurred over a substantially longer timescale [109]. This example represents how machine learning can extend the utility of multifunctional conductive gels from detecting an overall electrical response to identifying the applied stimulus conditions. However, the reported accuracy supports classification within the tested stimulus set, while quantitative separation of individual stimulus magnitudes and robustness under concurrent humidity variations require further evaluation.

More generally, machine-learning models for conductive gel-based sensors may encounter signal patterns during on-body use that differ from those represented in the training data [110]. Variations in skin temperature, perspiration, and movement may alter sensor responses, potentially affecting the features used for classification or estimation. Model robustness can therefore be assessed under combined environmental and physiological variations, for example, using test data from recording sessions not included in model training [111].

### 3.4. Chemical and Biochemical Sensing

Chemical and biochemical sensing in conductive gels is based on the conversion of analyte-induced changes in charge transport, interfacial electrochemistry, or polymer-network state into measurable electrical signals. The liquid-rich and porous network of a conductive gel provides pathways for the diffusion of target species while accommodating ionic or electronic conductors and chemically responsive components. Therefore, the gel can function simultaneously as an analyte-permeable matrix, a host for recognition elements, and an electrical transduction layer. Depending on the sensing mechanism, the resulting responses can be measured as changes in current, potential, impedance, resistance, or conductance [75,112].

**Figure 7:**
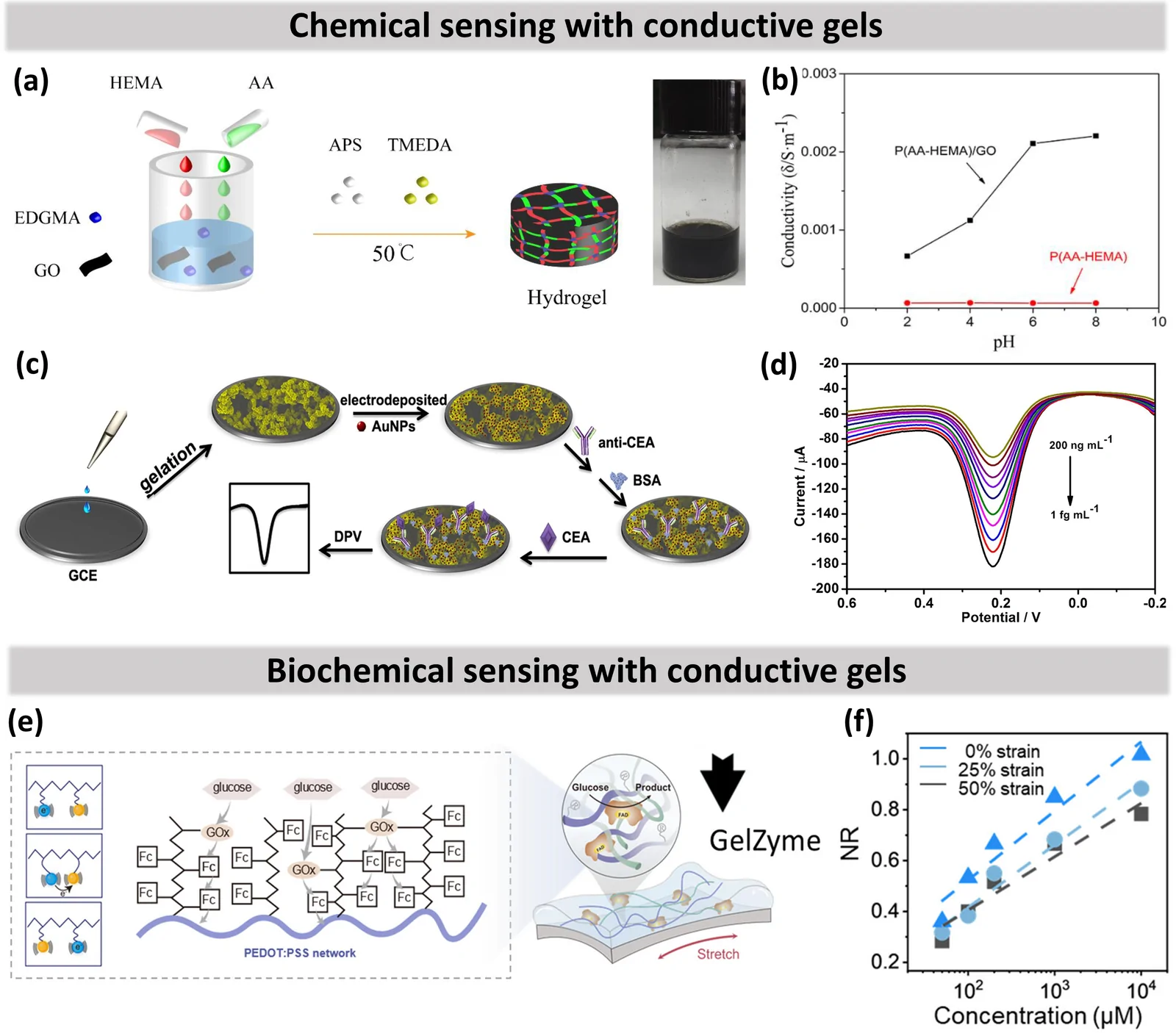
**Representative chemical and biochemical sensing systems based on conductive gels.** (**a**,**b**) pH-responsive electrical sensing using poly(acrylic acid-*co*-2-hydroxyethyl methacrylate)/graphene oxide [P(AA-HEMA)/GO] hydrogels. (**a**) Schematic preparation of the composite hydrogel. (**b**) Hydrogel conductivity as a function of pH. Adapted from Wang et al. [114]. © 2019 by the authors. Published by MDPI under a CC BY 4.0 license. (**c**,**d**) Electrochemical detection of carcinoembryonic antigen (CEA) using a gold-nanoparticle-decorated polypyrrole (PPy) hydrogel. (**c**) Fabrication and sensing procedure on a glassy carbon electrode (GCE). (**d**) Differential pulse voltammetry (DPV) responses at different CEA concentrations. Adapted from Rong et al. [112]. © 2015 Macmillan Publishers Limited. Published by Nature Publishing Group under a CC BY 4.0 license. (**e**,**f**) Stretchable glucose sensing using a mixed-conducting GelZyme. (**e**) GelZyme architecture integrating glucose oxidase (GOx), ferrocene (Fc) mediators, and PEDOT:PSS network. (**f**) Normalized current response (NR) as a function of glucose concentration at 0, 25, and 50% strain. Adapted from Cui et al. [126]. © 2026 The Author(s). Published by Wiley-VCH GmbH under a CC BY license.

Chemical sensing generally relies on the direct interaction of target species—including hydrogen ions, dissolved ions, gases, metal ions, or organic contaminants—with functional groups or responsive components incorporated into the gel [113]. These interactions can change the ionization state of the polymer, the concentration or mobility of mobile charge carriers, the swelling state of the network, or the electrical properties of the gel–electrode interface. Consequently, the chemical stimulus can be transduced into changes in ionic conductivity, resistance, or impedance. Wang et al. prepared a pH-responsive conductive hydrogel consisting of a poly(acrylic acid-co-2-hydroxyethyl methacrylate) [P(AA-HEMA)] network and graphene oxide (GO) (Figure 7a). As the pH increased from 2 to 8, progressive ionization of the carboxyl groups increased electrostatic repulsion within the polymer network, resulting in pronounced hydrogel swelling, particularly above pH 4. An increase in electrical conductivity was observed over the same pH range (Figure 7b). However, this behavior should not be interpreted simply as swelling-enhanced electron transport through the GO network. Swelling can alter the ionic environment and solvent content while also changing the spacing and connectivity of GO-containing conductive domains. Thus, the ionic contribution may change through polymer ionization and mobile-ion transport, whereas the electronic contribution depends on the connectivity and spacing of the GO-containing conductive pathway and does not necessarily increase with swelling. Therefore, the observed conductivity response is more appropriately interpreted as a system-specific result arising from the combined effects of polymer ionization, solvent uptake, ionic transport, and structural changes in the GO-containing conductive pathway. The pH-dependent volume change was also used to reversibly switch a circuit between off and on states at pH 2 and 8, respectively, demonstrating the conversion of a chemical stimulus into both electrical and dimensional responses [114].

Responsive polymers, ionic liquids, dyes, and functional nanomaterials may be introduced to impart affinity toward a particular chemical species or to amplify the resulting physicochemical change [76,112,113,114]. Accordingly, the magnitude and rate of the response can be governed by factors such as the chemical affinity of the sensing components [115], the hydrophilicity [116] and porosity [117] of the network, the gel thickness [118], the ionic composition [119], and the diffusion distance of the analyte [120]. For example, Rong et al. constructed an electrochemical biosensing platform by electrodepositing Au nanoparticles (AuNPs) onto a three-dimensional network of PPy hydrogel. As illustrated in Figure 7c, the interconnected porous hydrogel provided a large effective surface area and open channels for the transport of electrons, ions, and target molecules, while the AuNPs further increased the electrical conductivity, interfacial electron transfer, and amount of immobilized antibody. After anti-carcinoembryonic antigen (anti-CEA) was immobilized on the composite, its specific binding with carcinoembryonic antigen (CEA) formed a nonconductive protein layer that impeded interfacial electron transfer, resulting in a measurable decrease in the electrochemical current. The open network and continuous conductive framework shortened the diffusion pathway and enhanced signal transduction, enabling a linear detection range of 1 fg∙mL^−1^ to 200 ng∙mL^−1^ and a detection limit of 0.16 fg∙mL^−1^ (Figure 7d) [112].

Biochemical sensing relies on the selective recognition of target analytes by biological elements, such as enzymes, antibodies, aptamers, or other nucleic-acid-based receptors, incorporated into the conductive gel network or immobilized at the gel–electrode interface [121]. Depending on how molecular recognition generates the sensing response, these systems can be broadly discussed in terms of enzyme-mediated catalytic sensing and affinity-based sensing. In enzyme-functionalized systems, catalytic reactions involving metabolites such as glucose or lactate can alter local redox activity, ionic conductivity, charge-transfer behavior, or other electrochemical properties, producing responses such as changes in current, potential, or impedance [75,122]. In affinity-based systems, selective binding of a target molecule to a receptor, such as an antibody or aptamer, changes the charge distribution, ion transport, or interfacial charge-transfer characteristics without necessarily consuming the analyte. These changes can also be detected through electrical outputs such as current, potential, or impedance [123,124]. The hydrated and permeable gel network can support both mechanisms by facilitating the transport of analytes and reaction products while retaining the recognition elements near the electrical transducer [125].

Accordingly, conductive gels can serve as biochemical-sensing platforms by permitting analyte transport, immobilizing recognition elements, maintaining conformal contact with the sensing interface, and transferring analyte-induced ionic or electronic changes to external electrodes for electrical readout. For instance, Cui et al. introduced enzymatic hydrogel electrodes, termed GelZymes, that integrate the enzyme membrane and electrode into a monolithic three-dimensional structure. As illustrated in Figure 7e, a porous and stretchable PAA/PAAm network facilitated glucose diffusion and enabled the immobilization of glucose oxidase and ferrocene mediators, whereas a secondary poly(3,4-ethylenedioxythiophene):poly(styrene sulfonate) network provided electronic conduction. During sensing, glucose oxidation by glucose oxidase generated electrons that were transferred either directly or through the ferrocene mediators to the conductive polymer network, producing an amperometric glucose response. The tissue-like viscoelasticity and stretchability of the GelZymes further enabled glucose sensing under deformation, with the sensor maintaining a clear glucose concentration-dependent response even at 50% strain (Figure 7f). Using these properties, the authors constructed a tape-based continuous glucose monitor that could be readily attached to the skin for noninvasive sweat-glucose monitoring [126].

Conductive gels can integrate selective molecular recognition with mechanically compliant electrical transduction, supporting biochemical monitoring at deformable biological interfaces [126]. However, the accuracy of biochemical quantification can be affected by cross-sensitivity to non-target species and changes in the sampled fluid conditions. Temperature and pH can affect enzymatic activity, while coexisting electroactive species may contribute interfering signals [127]. Nonspecific adsorption can further alter the measured response independently of the target-analyte concentration [123]. Accordingly, calibration and interference testing in representative biological matrices can help assess quantification errors under relevant combinations of temperature, pH, and interferent levels [128].

Across the sensing modes discussed in Section 3, practical performance depends on factors such as the transduction mechanism, gel composition, device design, target stimulus or analyte, and intended operating conditions. Table 2 summarizes the dominant electrical signals, sensitivities, operating ranges, limitations, and suitable applications of representative conductive gel-based sensors, organized by sensing mode and transduction mechanism.

## 4. Device Interfaces of Conductive Gels

Although Section 3 organizes conductive gel-based sensors primarily according to the target stimulus or analyte, the underlying transduction mechanisms are not unique to gels and are also widely used in silicone- and other elastomer-based sensors [133,134]. The distinctive value of conductive gels becomes more evident at biological and device interfaces, where their liquid-filled polymer networks directly influence contact and transport [7,24,68]. Conductive gels can be engineered to combine low-modulus conformability with strong adhesion to skin or tissue [135]. In particular, hydrogel-based systems also provide high water content, ion-rich contact, and pathways for the transport of water, ions, and biochemical species [4,7,19]. Together, these features can reduce interfacial gaps, facilitate bioelectrical coupling and analyte exchange, and help maintain contact during deformation in ways that are less readily achieved with conventional dry elastomer interfaces [7,23].

**Figure 8:**
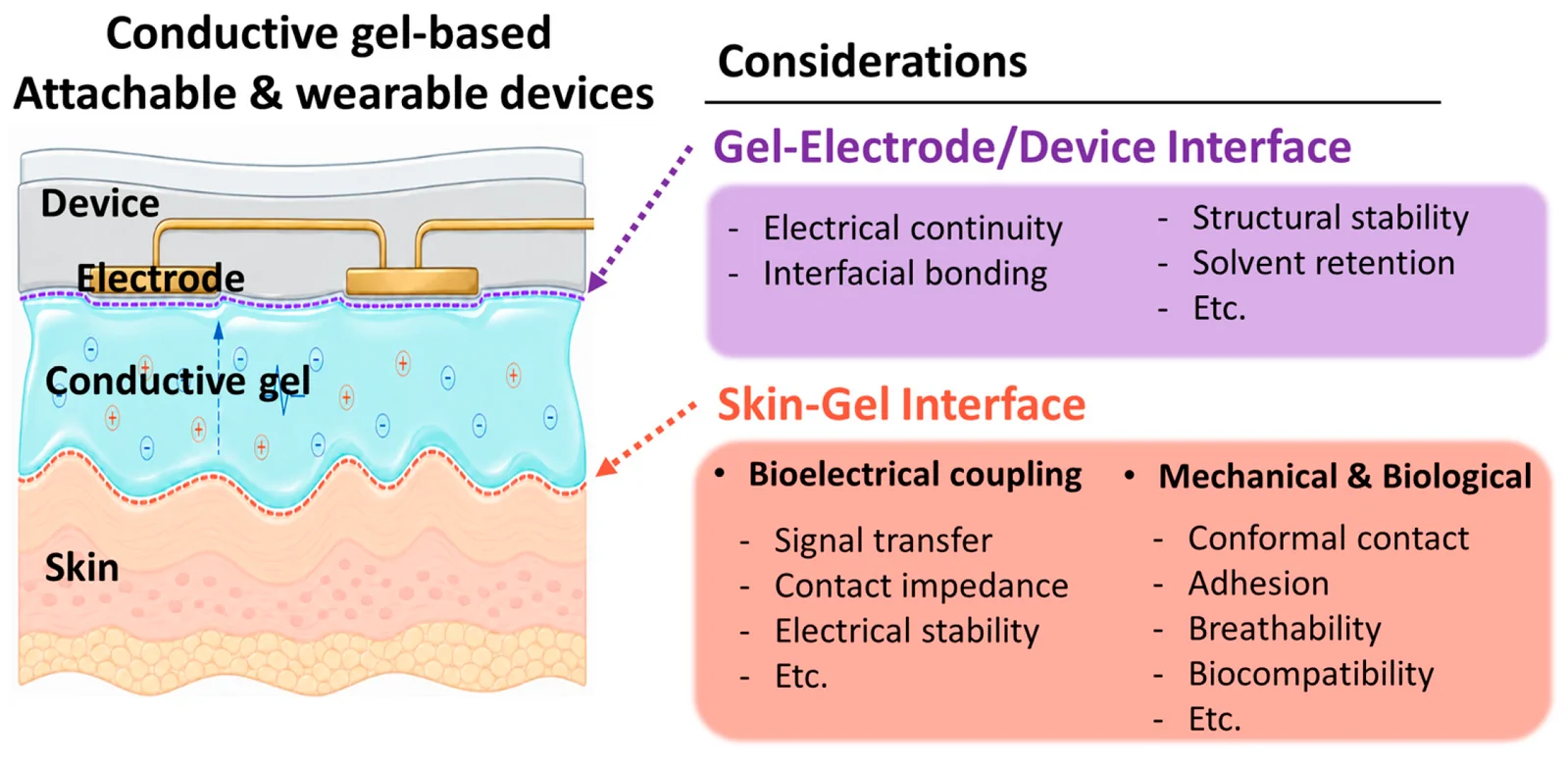
**Schematic illustration of the skin–gel and gel–electrode/device interfaces in conductive gel-based wearable and attachable healthcare devices and the key considerations associated with each interface.** The graphical illustration of the skin–gel–device interfaces was generated using ChatGPT (GPT-5.6, OpenAI), while the overall organization, labels, and annotations were developed by the authors.

In wearable and attachable healthcare devices, performance therefore depends not only on the conversion of external stimuli into electrical outputs but also on the transfer of electrical signals, mechanical loads, and materials across the skin–gel and gel–device interfaces, as illustrated in Figure 8. At the skin-contacting surface, these interfacial properties of conductive gels can support conformal contact with irregular and deformable skin; however, reliable signal acquisition requires stable electrical coupling and mechanical contact while minimizing motion artifacts and adverse biological responses, such as skin irritation, allergic reactions, and inflammation, during prolonged use [136,137]. Also, within the device, the interfaces between the gel and components such as electrodes, interconnects, dielectric layers, substrates, and encapsulation layers must maintain electrical continuity and structural integrity under repeated deformation, solvent migration, swelling, and environmental exposure [138,139]. Instability at these interfaces can cause contact loss, delamination, impedance variation, baseline drift, and eventual device failure even when the bulk gel retains adequate conductivity and mechanical properties [10,139]. Therefore, interface design should be considered together with gel composition and network structure to achieve reliable long-term operation.

Accordingly, this section discusses the device interfaces of conductive gels in terms of three interconnected interface types: (i) bioelectrical coupling at the skin–gel interface, which governs the transfer and acquisition of electrophysiological signals; (ii) mechanical and biological skin interfaces, which determine conformal contact, adhesion, resistance to motion, and compatibility with prolonged skin attachment; and (iii) gel–electrode and device interfaces, which control electrical communication, interlayer bonding, solvent retention, and structural stability within integrated devices.

### 4.1. Bioelectrical Coupling at the Skin–Gel Interface

Bioelectrical coupling at the skin–gel interface refers to the transfer of physiological electrical potentials from the body surface to a wearable device. Electrophysiological signals, including ECG, EMG, EEG, and EOG signals, originate from ionic activity in biological tissues and appear at the skin surface as weak, frequency-dependent potentials [4]. Because these signals are readily affected by interfacial losses and external noise, their reliable acquisition depends not only on the magnitude of the biological potential but also on the electrical boundary established between the skin and the electrode. When a relatively rigid or poorly conforming electrode is placed on the skin, microscopic gaps can reduce the effective contact area, increase interfacial impedance, and consequently attenuate the recorded signal or lower the signal-to-noise ratio (SNR) [140,141].

Conductive gels can serve as hydrated, deformable interlayers that couple the ionically conductive body to the external recording system while conforming to the irregular skin surface. For example, Li et al. fabricated an approximately 2-μm-thick biomimetic “super tattoo” comprising an electrospun silk-fibroin scaffold and an ionically conductive poly(sulfobetaine methacrylate)-deep eutectic solvent (PSBMA-DES) gel matrix. The composite exhibited strong adhesion and skin-like strain-stiffening behavior, and maintained conformal contact with the skin even during deformation. The substrate was used to prepare MXene-, PEDOT:PSS-, and Au-based tattoo electrodes. In particular, the Au-based electrodes recorded EMG signals during empty, regular, and effortful swallowing, which involve substantial neck-skin deformation, and exhibited lower motion artifacts than commercial Ag/AgCl electrodes [142].

Using a different approach, Wang et al. developed a thermoreversible, paintable gelatin-based biogel containing NaCl and sodium citrate in a water-glycerol solvent system. Upon heating, the biogel became fluidic, allowing it to flow between scalp hairs and follow the microscopic corrugations of the skin; it subsequently gelled in situ to form an adhesive viscoelastic interface conformal to the hairy scalp. The painted biogel immobilized flexible Ag/AgCl electrodes without additional mechanical fixtures. EEG alpha rhythms measured after 0, 24, 48, and 72 h of continuous electrode wear retained distinct periodic patterns with a peak frequency of 10 Hz, demonstrating the suitability of the interface for multiday EEG monitoring [143].

Even when conformal contact is established, reliable bioelectrical recording requires the electrical impedance along the skin–gel–electrode pathway to remain low and stable. This impedance includes contributions from the skin–gel contact, conduction through the gel, and the gel–electrode boundary, and its magnitude varies with measurement frequency because these components exhibit both resistive and capacitive behavior [140,144]. High or unstable impedance can weaken electrical coupling with the skin and increase the influence of electrical noise, thereby reducing the SNR.

**Figure 9:**
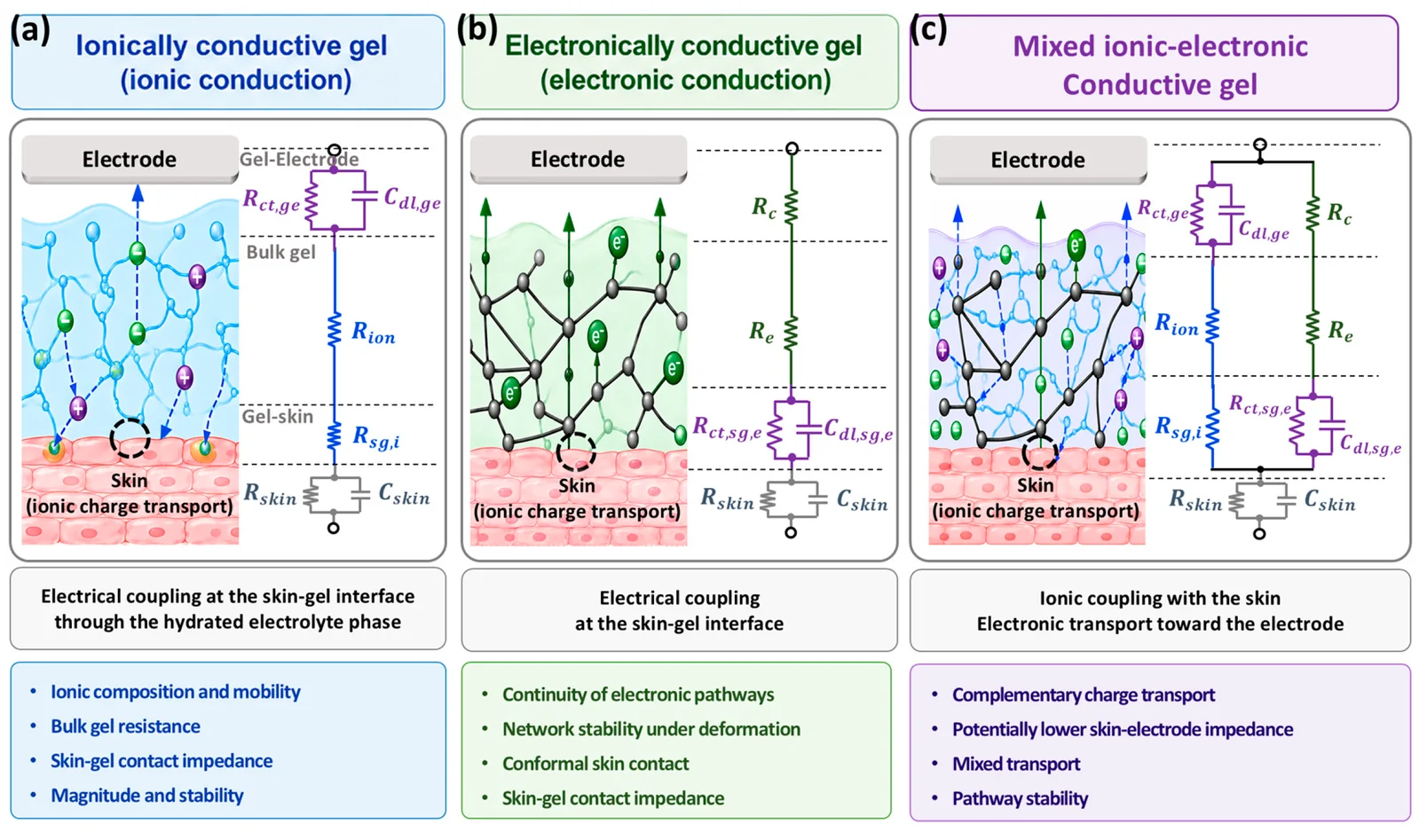
**Schematic comparison of charge-transport pathways, corresponding illustrative equivalent circuits, and key factors governing the magnitude and stability of impedance across the skin–gel–electrode assembly for (a) ionically conductive gels, (b) electronically conductive gels, and (c) mixed ionic-electronic conducting gels.** The illustrative equivalent circuits assume small-signal conditions and interfacial access to the depicted conducting phases, with bulk and contact resistances treated as ohmic. The equivalent circuit in panel (**c**) represents a simplified parallel-pathway approximation without explicit distributed ionic-electronic coupling. Subscripts ge and sg identify the gel–electrode and skin–gel interfaces, while i and e distinguish ionic and electronic pathways. At sg,e, the electronic network interfaces with the skin-side electrolyte. R_ion_ and R_e_ denote bulk ionic and electronic resistances, respectively. R_c_ represents electronic network-collector contact resistance, and R_sg,i_ represents ionic access/contact resistance. R_ct_ and C_dl_ denote Faradaic charge-transfer resistance and double-layer capacitance, respectively. R_skin_ and C_skin_ represent effective epidermal resistance and capacitance. Horizontal dashed lines delineate the modeled regions. The graphical illustrations were generated using ChatGPT (GPT-5.6, OpenAI), while the overall layout, labels, and annotations were developed by the authors.

As shown in Figure 9, the overall impedance and the corresponding strategies for reducing it vary with the conduction mechanism of the gel. In ionically conductive gels, mobile ions transport charge through the liquid phase confined within the polymer network [20]. At the skin–gel interface, bioelectric potentials reaching the skin surface are coupled to ionic conduction within the gel through electrolyte-mediated contact [7,145]. Accordingly, to establish and maintain reliable electrical coupling at the skin–gel interface, factors such as ionic composition and mobility [145], resistance within the gel [146], and the magnitude and stability of the skin–gel contact impedance [140] should be considered. For example, Zheng et al. introduced a skin-printable ionic hydrogel bioelectrode that reduced both intrinsic and skin-contact impedance by combining a low-impedance ionic composition with conformal in situ gelation. The hydrogel, denoted GGW-NH_4_Cl, comprised gelatin, glycerol, water, and ammonium chloride (NH_4_Cl). Its porous three-dimensional network provided ion-transport pathways, and the optimized hydrogel exhibited an ionic conductivity of 0.031 S∙m^−1^, compared with 0.019 S∙m^−1^ for a commercial conductive paste and 0.016 S∙m^−1^ for an adhesive gel. Applied in its fluid state, the hydrogel conformed to the skin microtopography before in situ gelation and established intimate interfacial contact. Consequently, its skin-contact impedance measured using a three-electrode method was 9.5 kΩ at 100 Hz, compared with 16.1 kΩ for the commercial gel. It also enabled EEG and EMG recording and retained a conductivity of approximately 0.03 S∙m^−1^ with a stable EMG SNR after five days [145]. These results indicate that both bulk ionic transport and conformal interfacial contact contribute to stable bioelectrical coupling.

In electronically conductive gels, conductive polymers or interconnected fillers provide electronic pathways from the skin-contacting region to the external electrode [4,147]. Because electrical coupling between the ionically conductive skin and the electronically conductive gel occurs at the skin–gel boundary, maintaining the continuity and deformation stability of the electronic pathways within the gel can help ensure reliable signal transmission. For example, Yi et al. demonstrated that incorporating AgNWs into an ultrathin PVA matrix to improve the continuity and deformation stability of electronic conduction pathways within the gel electrode could reduce skin–gel contact impedance. The AgNWs formed a percolated conductive network, while the toughness and resilience of the PVA matrix enabled the network to reconstruct after local disruption during deformation. Consequently, the relative resistance response remained nearly unchanged after 500 stretching-release cycles at 40% strain. The 14.7-μm-thick electrode conformed to microscopic skin undulations and exhibited skin–gel contact impedances of 50.4 and 28.6 kΩ at 10 and 100 Hz, respectively, which were 59% and 45% lower than those of a commercial gel electrode. Its contact impedance remained nearly constant after 8 h of wear. The electrode was further demonstrated for EMG, ECG, and EEG recording. During 3 h of continuous ECG monitoring, signal amplitudes were stable during sleep and PC work, with slight fluctuations during exercise, while waveforms remained clear across these activities [147].

As another approach, mixed ionic–electronic conducting gels have been introduced to support complementary charge-transport processes within a single material. Ionic transport through the hydrated phase can couple with the ionically conductive skin, whereas the electronically conductive network transfers charge toward the external electrode, potentially reducing the electrical resistance of the overall skin-electrode connection. For example, O’Neill et al. developed a supramolecular conductive hydrogel that exhibited homogeneous mixed ionic-electronic transport and low on-skin impedance. Hydrophilic self-doped PEDOT (S-PEDOT) was polymerized within a PAAm-based supramolecular polymer network crosslinked through high-affinity cucurbit[8]uril (CB[8]) host-guest complexes, enabling its homogeneous distribution throughout the hydrated network. By contrast, an analogous PEDOT:PSS-based supramolecular polymer network exhibited inhomogeneous aggregation and domain separation with limited interconnectivity between PEDOT-rich domains. The homogeneous S-PEDOT network formed interconnected electronic conduction pathways and exhibited a conductivity of 5.8 S∙cm^−1^, compared with 0.52 S∙cm^−1^ for the PEDOT:PSS-based network. When patterned to match the active area of commercial EMG/ECG electrodes and applied to the inner forearm, the S-PEDOT hydrogel exhibited an impedance of approximately 1 × 10^4^ Ω at frequencies below 50 Hz. The corresponding values were approximately 5 × 10^4^ Ω for the PEDOT:PSS hydrogel and 10^5^–10^6^ Ω for a commercial electrode. The authors suggested that the greater abundance of sulfonic acid groups in the S-PEDOT network was associated with a higher concentration of mobile ions than in the PEDOT:PSS network. These ions may have contributed to its low on-skin impedance, while the homogeneous S-PEDOT network provided continuous electronic charge-transport pathways through the gel. The S-PEDOT hydrogel electrodes also successfully recorded surface EMG and ECG signals, demonstrating their applicability to cutaneous bioelectrical monitoring [148].

For long-term use of conductive gel-based wearable and attachable healthcare devices, maintaining stable impedance over time is as important as achieving a low initial value [149]. Solvent loss can dehydrate and shrink the gel, decrease ionic mobility, and produce contact gaps, thereby increasing interfacial impedance and baseline drift [68,150]. Conversely, sweat or high ambient humidity can cause local water uptake, swelling, and changes in ionic concentration, gel geometry, and interfacial adhesion [151]. Repeated skin deformation and electrode displacement can independently change the contact area and pressure or disrupt conductive pathways, resulting in time-dependent impedance variations and motion artifacts [141,152,153].

Long-term electrical coupling therefore requires coordinated control of factors such as solvent and ion retention, evaporation, swelling and ion leakage, sweat and water-vapor transport, skin adhesion, and the stability of conductive pathways under repeated deformation. For example, Wang et al. addressed these requirements using an organohydrogel nanofilm with a thickness of 392 nm, comprising a polyurethane nanomesh, gelatin crosslinked with genipin, and a glycerol/NaCl/tannic acid electrolyte. The electrolyte limited solvent loss, the ultrathin hydrophilic architecture supported sweat transport and conformal adhesion, and the nanomesh and covalent network improved mechanical stability. The nanofilm retained 96.3% of its mass after 200 days at room temperature and maintained stable resistance over 2500 stretching cycles. After 20 min of running, skin contact impedance recovered during rest, and the electrode maintained an average ECG SNR above 32.2 dB from days 2 to 9 [68]. This example demonstrates that prolonged bioelectrical recording depends on coordinated material, interface, and mechanical design rather than solvent retention alone.

Accordingly, the evaluation of improvements at the skin–gel interface needs to extend beyond short-term measurements to establish whether these benefits are sustained during prolonged wear, taking into account factors such as solvent and ion retention, sweat transport, interfacial adhesion, and conductive pathway stability. Therefore, parameters such as bulk conductivity, skin–gel contact impedance, and SNR can be compared before and after repeated mechanical or environmental cycling to assess changes relative to their initial values. Recovery after a defined period can also be considered alongside indicators such as baseline drift, motion artifacts, and preservation of physiological waveform or spectral features [68,141]. Comparisons across studies also require consideration of factors such as measurement frequency, electrode area, skin preparation, and signal acquisition and processing conditions [154,155].

### 4.2. Mechanical and Biological Skin Interfaces

**Figure 10:**
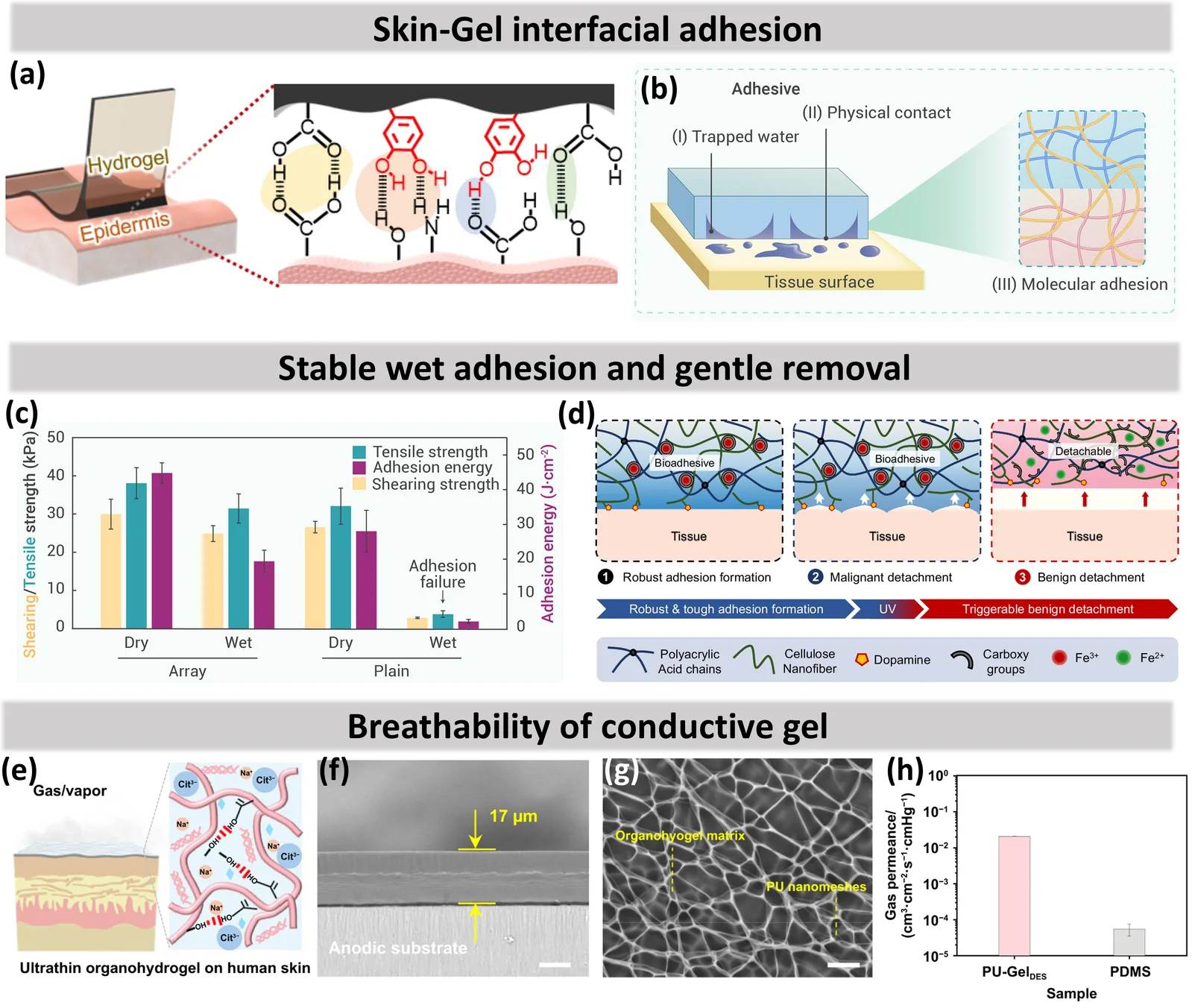
**Representative mechanical and biological considerations at skin–gel interfaces.** (**a**) Hydrogen-bond-mediated adhesion between catechol and carboxyl groups of a dopamine-functionalized pectin/poly(acrylic acid)/polydopamine-coated reduced graphene oxide (PT-DA/PAA/rGO-PDA) hydrogel and functional groups on the epidermal surface. Adapted from Kondaveeti et al. [158]. © The Author(s) 2024. Published by Springer Nature under a CC BY 4.0 license. (**b**,**c**) Skin adhesion of a multi-bioinspired optoelectronic skin (OE-skin) incorporating an octopus-inspired adhesive array. (**b**) Suction-cup-like hemispherical microstructures enabling adhesion at the skin–gel interface through interfacial water trapping, physical contact, and molecular adhesion. (**c**) Tensile and shear adhesion strengths and adhesion energy of array and plain patches on dry and wet skin. Adapted from Li et al. [159]. © The Author(s) 2025. Published by Elsevier Inc. on behalf of Youth Innovation Co., Ltd. under a CC BY 4.0 license. (**d**) Light-driven supramolecular-network reconfiguration enabling robust adhesion and ultraviolet-triggered benign detachment of a cellulose nanofiber-dopamine/poly(acrylic acid)@Fe^3+^ (CNF-DA/PAA@Fe^3+^) hydrogel. Adapted from Zhang et al. [172]. © The Author(s) 2024. Published by Springer Nature under a CC BY 4.0 license. (**e**–**h**) Breathability of an ultrathin PU nanomesh-reinforced organohydrogel containing a deep eutectic solvent (PU-GelDES). (**e**) Gas and vapor exchange through the organohydrogel on skin and its internal ionic-transport pathways. (**f**) Cross-sectional scanning electron microscopy (SEM) image of the ultrathin organohydrogel on an anodic substrate. (**g**) SEM image of the organohydrogel matrix reinforced with PU nanomeshes. (**h**) Gas permeance of PU-GelDES and polydimethylsiloxane (PDMS). Adapted from Yang et al. [173]. © The Author(s) 2025. Published by IOP Publishing Ltd on behalf of the IMMT under a CC BY 4.0 license.

Beyond the bioelectrical processes described above, practical skin coupling depends on whether the conductive gel can maintain mechanically stable and biocompatible contact throughout wear. Skin is soft, curved, microscopically irregular, and continuously deformed during daily activity. Therefore, a gel that is excessively stiff or exhibits insufficient mechanical recovery may lead to incomplete contact, interfacial sliding, or local detachment under repeated deformation [7,152]. Consequently, mechanical matching between the skin and the gels should be evaluated using parameters such as Young’s modulus, stretchability, elastic recovery, hysteresis, toughness, and fatigue resistance rather than modulus alone [10]. These mechanical properties influence how effectively the gel deforms with the skin, while stable interfacial coupling also requires conformal contact with the curved and microscopically irregular skin surface [141,156]. Gel thickness and surface geometry affect the extent of conformal contact, and adhesion helps preserve this contact during motion [68,157]. Such adhesion can be promoted through various approaches. Representative examples include hydrogen-bond-mediated adhesion at the skin–gel interface (Figure 10a) [158] and bioinspired surface structures, such as suction-cup-like microstructures (Figure 10b) [159]. Depending on the material composition and surface chemistry, other interfacial mechanisms, such as electrostatic interactions [160], van der Waals forces [68], π-π interactions [161,162], and covalent coupling between reactive groups [151,163], may also contribute to adhesion, either individually or in combination. Table 3 summarizes the reported skin–gel adhesion performance of representative conductive gels. Although the reported adhesion values are expressed in a common unit of kPa, differences in test configurations and testing conditions limit direct quantitative comparisons across studies. Adhesion also depends on factors such as peel angle, contact area, preload, contact time, loading rate, skin substrate and hydration state, and dry or wet testing conditions. Nevertheless, the comparison illustrates how different conductive gel formulations and interfacial designs can support skin adhesion, helping guide material selection and the design of adhesion strategies for the intended attachment conditions.

**Table 3:** Reported skin–gel adhesion performance of representative conductive gels.

Principal Adhesion Mechanism	Conductive Gel/System	Representative Adhesion Performance	Skin Substrate and Test Condition	Refs
Hydrogen bonding	Borate-crosslinked carboxymethyl cellulose-dopamine conjugate (CDB)/polyacrylamide (PAAm)/poly(3,4-ethylenedioxythiophene):poly(styrene sulfonate) (PEDOT:PSS) catechol hydrogel (CDB-PAAm-PEDOT:PSS)	65.1 ± 2.2 kPa	Porcine skin; adhesion-strength test	[164]
Hydrogen bonding	Poly(vinyl alcohol) (PVA)/borax/pectin/tannic acid (TA) ionotronic hydrogel	55 kPa	Porcine skin; normal adhesion-strength test	[165]
Hydrogen bonding	PVA/borax/PEDOT:PSS hydrogel	19.6 kPa	Pig skin; adhesion-stress test	[166]
Hydrogen bonding	Poly(2-acrylamido-2-methylpropane sulfonic acid) (PAMPS)/PEDOT:PSS/dimethyl sulfoxide (DMSO)/glycerol hydrogel electrode	~12 kPa	Human skin; tensile adhesion test	[167]
Electrostatic and dipolar interactions	Anion-zwitterion hydrogel formed from 2-acrylamido-2-methyl-1-propanesulfonic acid sodium salt (AMPSs), 3-(1-vinyl-3-imidazolio)propanesulfonate (VIPS), and acrylamide (AAm) [P(AMPSs/VIPS-co-AAm)]	26.29 kPa	Porcine skin; lap-shear test	[168]
Ionic/electrostatic interactions/hydrogen bonding	Poly[2-acrylamido-2-methylpropanesulfonic acid (AMPS)-co-acrylamide]/chitosan quaternary ammonium salt (HAAC) polysaccharide hydrogel conductor [P(AMPS/AAm)-HAAC]	69.33 kPa	Porcine skin; repeated 90° peel test	[169]
Electrostatic interactions	Poly(acrylic acid) (PAA)-chitosan (CHI)-aluminum ion (Al^3+^)-MXene hydrogel adhesive layer (PCAM) with a hydrophobic backing	388.1 kPa	Porcine skin; underwater adhesion-strength test	[160]
Conformability-enhanced van der Waals interactions	0.1 mg·cm^−2^ polyurethane (PU) nanomesh-reinforced, genipin-crosslinked gelatin organohydrogel containing glycerol, sodium chloride, and TA (PU_0.1_-GHGNT)	0.44 kPa (44 mN at 1 cm^2^)	Human skin; tack-separation test	[68]
π-π + covalent + hydrogen/coordination bonding	PEDOT/lignosulfonate (LS) nanoparticle-incorporated PAAm hydrogel (PEDOT/LS-PAAm)	20 kPa	Porcine skin; adhesion-strength test	[161]
π-π-assisted cohesion + hydrogen bonding	PEDOT-PAM-polydopamine (PDA) hydrogel	14.2 kPa	Porcine skin; shear-strength test	[162]
Covalent coupling between reactive groups	Ca^2+^-modified silk fibroin (SF)/poly(acrylic acid) grafted with *N*-hydroxysuccinimide ester (PAA-NHS) double-network hydrogel (BioSP)	40.02 kPa	Porcine skin; lap-shear tests; artificial-sweat conditions	[151]
Covalent coupling between reactive groups	MXene/PAA/alginate-3,4-dihydroxy-L-phenylalanine (DOPA)/dopaquinone/LiCl organohydrogel (SRBSS)	~1040 kPa	Wet porcine skin; lap-shear tests	[163]
Bioinspired adhesive surface structure	PAAm double-network (PAAm-DN) backing/poly(N-isopropylacrylamide) (PNIPAm) double-network (PNIPAm-DN) microsuction-cup array/gelatin-silver (Ag)-tannin adhesive patch	>30 kPa	Ex vivo pigskin; vertical tensile separation; reversible over five cycles	[170]
Bioinspired adhesive surface structure	CNT/PAAm-gelatin double-network hydrogel with a PNIPAm hemispherical microarray	~38 kPa (dry); ~31 kPa (wet)	Pig skin; tensile adhesion; dry and wet interfaces evaluated	[159]
Bioinspired adhesive mechanism	Mussel-inspired dopamine-functionalized pectin (PT-DA)/PAA/polydopamine-coated reduced graphene oxide (rGO-PDA) hydrogel with Fe^3+^ ionic crosslinking (PPGP)	85 kPa	Ex vivo porcine skin; normal pull-off, 1 × 1 cm^2^ contact area, 20 kPa preload, 10 s contact	[158]

These adhesion mechanisms can help preserve an effective skin–gel contact area and suppress interfacial sliding or local lifting during body motion, thereby reducing contact instability and motion artifacts [152,171]. However, adhesion should not simply be maximized; it should be sufficient to maintain stable attachment under both dry and sweat-exposed conditions and allow gentle, residue-free removal without imposing excessive stress on the skin. To achieve stable adhesion under wet skin conditions, Li et al. developed a multi-bioinspired optoelectronic skin (OE-skin) that integrated a CNT-hybridized PAAm-gelatin double-network conductive hydrogel substrate with a thermoresponsive poly(N-isopropylacrylamide) (PNIPAm) hemispherical adhesive array. The octopus-inspired array enabled wet adhesion by trapping interfacial water, generating localized negative pressure, and promoting interfacial chain interactions during near-infrared (NIR)-induced heating and cooling. The NIR-responsive system also enabled reversible switching between adhesive and non-adhesive states. As shown in Figure 10c, in adhesion tests on pig skin, the array-containing OE-skin exhibited tensile strengths of approximately 38 and 31 kPa and shear strengths of approximately 30 and 25 kPa under dry and wet conditions, respectively. The wet-skin performance corresponded to approximately 84% of the tensile strength and 63% of the adhesion energy measured under dry conditions. By contrast, an array-free plain patch adhered well to dry skin but failed to maintain adhesion on wet skin because of interfacial water [159].

Complementing this strategy for maintaining adhesion under wet and repeated mechanical conditions, Zhang et al. addressed the need for residue-free removal by enabling reversible regulation of interfacial adhesion (Figure 10d). They developed a cellulose-nanofiber-reinforced poly(acrylic acid) hydrogel containing dopamine-derived adhesive groups and Fe^3+^ coordination crosslinks (CNF-DA/PAA@Fe^3+^). Ultraviolet (UV) irradiation reduced Fe^3+^ to Fe^2+^ and induced the dissociation and reconfiguration of the coordination interactions, decreasing the interfacial toughness on skin by more than 92%, from 89.13 to 5.82 J∙m^−2^. Adhesive performance recovered after 5 min of air oxidation, and reversible adhesion-photodetachment switching was maintained over five ultraviolet-irradiation-air-oxidation cycles. On the skin, UV irradiation at an intensity of ≤40 mW∙cm^−2^ for 5 min enabled gentle, low-force removal, after which no visible residue or redness was observed [172]. Consequently, these studies demonstrate that the design of conductive skin–gel interfaces should consider not only initial adhesion strength but also complementary strategies for maintaining reliable adhesion under wet and repeated mechanical conditions and for enabling controlled removal with minimal observable effects on the skin.

For prolonged use of conductive gel-based wearable and attachable devices, intimate contact should not produce an excessively occlusive microenvironment. Accumulation of water vapor and sweat beneath the device can alter local skin hydration, destabilize adhesion, change the hydration and ionic state of the gel, and increase the risks of skin irritation and device failure [136,137]. To address these issues, strategies to improve the breathability of conductive gels have been explored, such as the use of ultrathin and porous architectures to facilitate air and water-vapor transport [156] and the incorporation of directional microstructures or liquid-transport pathways to promote sweat removal during physical activity [171]. However, the transport pathways that improve breathability can also facilitate evaporative water loss from water-rich gels under dry conditions, potentially compromising their mechanical compliance and electrical stability [174,175]. This trade-off between breathability and solvent retention depends on both structural permeability and the water-retaining capacity of the gel formulation. Combining a permeable architecture with a moisture-retaining solvent system can help address both requirements [173]. As an example of this combined approach, Yang et al. developed a gas-permeable organohydrogel epidermal electrode by coating an electrospun PU nanomesh with a gelatin-deep eutectic solvent (DES) organohydrogel (Figure 10e). The glycerol and choline chloride components of the DES promoted moisture retention, complementing the vapor transport enabled by the ultrathin architecture. The resulting electrode had an ultrathin thickness of approximately 17 μm (Figure 10f), and its ultrathin architecture reinforced by the porous nanomesh provided pathways for gas and water-vapor transport (Figure 10g). As shown in Figure 10h, it exhibited a high gas permeance (2.1 × 10^−2^ cm^3^∙cm^−2^∙s^−1^∙cmHg^−1^), substantially higher than that of a 20-μm-thick polydimethylsiloxane (PDMS) film (5.5 × 10^−5^ cm^3^∙cm^−2^∙s^−1^∙cmHg^−1^), and a high water vapor transmission rate (1130.5 ± 67.1 g∙m^−2^∙day^−1^), approaching that of the open-boundary control (1316.2 ± 51.4 g∙m^−2^∙day^−1^) while also exhibiting excellent adhesion performance (135.9 μJ∙cm^−2^). Despite this high permeability, the DES-containing organohydrogel retained 98.6% of its initial mass after seven days in air, thereby combining water-vapor transmission across the electrode with retention of its internal solvent. The electrode also maintained stable skin-contact impedance and enabled continuous wireless ECG recording for 24 h with an SNR of 31.2 ± 1.2 dB, demonstrating its suitability for prolonged epidermal monitoring [173].

Complementing this permeability-based approach, Yang et al. developed a Nepenthes-inspired hydrogel (NIH) hybrid for directional sweat removal from the skin–device interface during exercise. A poly(vinyl alcohol)/poly(acrylic acid) (PVA/PAAC) double-network interface patterned with Nepenthes-inspired microcolumns and microgrooves increased capillary forces in the transport direction and suppressed reverse flow. Water droplets crossed the patterned interface within 0.23 s, approximately 4.5 times faster than the 1.03 s required for the unpatterned hydrogel. Directional transport persisted under stretching, bending, and S-shaped deformation. The hydrogel also retained approximately 88% of its initial mass after 96 h, demonstrating resistance to drying under the tested conditions. When integrated with Au-modified PI electrodes, the NIH layer maintained intimate skin contact during continuous artificial-sweat injection, whereas commercial and bare PI/Au electrodes detached within 5 s. The NIH electrodes exhibited skin-contact impedances of 3.62 and 4.01 kΩ at 1 kHz under dry and wet conditions, respectively, and maintained surface EMG SNR above 19 dB under vibration and perspiration. The wireless system also recorded ECG during exercise with heart-rate deviations below 2.6% relative to a commercial system, supporting stable electrophysiological monitoring during physical activity [171].

Collectively, the transport functions demonstrated in these studies can help reduce moisture accumulation at the skin-device interface and facilitate heat dissipation from covered skin, thereby improving thermal comfort during wear. Accordingly, breathability and solvent retention should be assessed together, with changes in gel hydration related to the stability of skin contact and electrical performance over the intended period of use [175].

Because conductive gels used in wearable and attachable devices can remain in direct contact with the skin for extended periods, biocompatibility is required to minimize adverse skin responses and support safe, sustained use [136,137]. The biocompatibility of skin-contacting devices can be evaluated through cytocompatibility tests and assessments of skin-contact responses, such as erythema, swelling, inflammation, or allergic reactions [176]. Importantly, the biocompatibility of a conductive gel should be assessed using the complete formulation and over contact periods representative of its intended use, rather than inferred solely from the hydrated nature of the polymer network. Biological responses may be influenced not only by the polymer network itself but also by residual or incorporated components, such as residual monomers, initiators, crosslinkers, solvents, ionic species, and conductive fillers, which may present distinct cytotoxicity or irritation risks [177]. Table 4 summarizes representative studies evaluating the biocompatibility of conductive gels used in wearable and attachable healthcare devices. Because these studies differ in biological models, exposure conditions, assay endpoints, and observation periods, their outcomes do not support direct quantitative ranking of biocompatibility. Even cell-viability percentages normalized to study-specific controls remain dependent on the cell type, assay, and exposure protocol. In vitro cytocompatibility, skin-contact responses, and implantation findings should therefore be interpreted as distinct forms of evidence, with conclusions limited to the conditions and durations examined. Nevertheless, the reported biological responses may provide insight into the potential suitability of different conductive gel formulations for skin-contacting devices, helping guide material selection within the limits of the available evidence.

During repeated use of conductive gel-based wearable and attachable devices, successive attachment-removal cycles can progressively alter both the mechanical contact and biological condition of the skin–gel interface [172]. For reuse to be practical, this interface must be reliably re-established after each application without deterioration of conformal contact, gel integrity, adhesion, electrical performance, or skin tolerability. Therefore, to guide improvements in the stability of the skin–gel interface and minimize progressive performance degradation over repeated use, attachment-removal or deformation cycles should be evaluated by considering factors such as adhesion retention, gentle and residue-free detachment [172], changes in gel hydration or geometry, and preservation of electrical and signal-recording performance [149,178]. Specifically, adhesion retention should be assessed relative to the initial adhesion strength under comparable conditions, considering factors such as substrate type and test configuration [179].

**Table 4:** Representative studies evaluating the biocompatibility of conductive gels for wearable and attachable healthcare devices.

Conductive Gel and Application	Biological Model and Exposure	Biocompatibility Tests	Key Biocompatibility Findings	Refs
Tannin-grafted gelatin/PAAm hydrogel + silver-tannin nanoparticles **Application:** Adhesive wound patch	**Model:** Human umbilical vein endothelial cells (HUVECs) **Exposure:** Gel leachate, 3 d	Live/Dead staining; Cell Counting Kit-8 (CCK-8) assay	High cell viability; metabolic activity comparable to control	[170]
PVA-borax/PEDOT:PSS/glycerol hydrogel **Application:** Wearable strain sensor; ECG, EMG, and EOG electrodes	**Model:** Human pulmonary arterial endothelial cells (HPAECs); human skin **Exposure:** Direct cell contact, 24–48 h; skin contact, ~10 min	3-(4,5-Dimethylthiazol-2-yl)-2,5-diphenyltetrazolium bromide (MTT) assay; neutral-red assay; skin-irritation assessment; removal assessment	Cell viability > 95%; no visible skin irritation	[180]
PVA/phytic acid/gelatin hydrogel; water-glycerol solvent **Application:** Epidermal strain sensor; ECG electrode	**Model:** Sprague-Dawley rat skin; human volunteers **Exposure:** Rat dorsal skin, 24 h; human forearm, 24 h	Erythema/edema observation; hematoxylin-and-eosin (H&E) histology; human patch-removal assessment	No erythema or edema; no epidermal damage or inflammatory infiltration	[181]
PAAm/TA/NaCl/silver nanoparticle hydrogel; water/ethylene glycol solvent **Application:** ECG/EOG/EEG interface; auditory brainstem response; polysomnography	**Model:** L929 fibroblasts; mouse skin **Exposure:** Cells, 24 h; mouse skin, 24 h	Cell-survival assay; H&E histology	Cell survival, 94.27%; no apparent skin inflammation	[182]
Sodium alginate/PAAm/KCl/glycerol/PEDOT:PSS double-network hydrogel **Application:** Semidry EEG electrode	**Model:** 3T3 fibroblasts; adult Japanese White rabbits **Exposure:** Gel extracts, 1–14 d; rabbit skin, 1–7 d	CCK-8 assay; skin-irritation assessment; H&E histology	Cell viability >100%; no erythema, edema, exudation, or histological inflammation	[157]
Asymmetric PVA/poly(ethylene glycol) (PEG)/NaCl bilayer hydrogel **Application:** Magnetic resonance imaging (MRI)- and computed tomography (CT)-compatible EEG electrode	**Model:** L929 fibroblasts; HaCaT keratinocytes; human dermal fibroblasts; rat skin/subcutaneous tissue **Exposure:** Cells, 1–3 d; rat skin, 3–12 h; subcutaneous implantation, 3 d	CCK-8 assay; Live/Dead staining; hemolysis; H&E/Masson histology; major-organ histology	Cell viability >90%; normal morphology; no substantial tissue inflammation or organ abnormalities	[183]
Poly(N-acryloyl glycinamide) (PNAGA)/Hydroxypropyltrimethyl ammonium chloride chitosan (HACC)/KCl/glycerol hydrogel **Application:** Antibacterial, reusable semidry EEG electrode	**Model:** 3T3 fibroblasts; Sprague-Dawley rats **Exposure:** Gel extraction, 3 d; cell exposure, 6 h; rat skin, 1–7 d	Extract-based CCK-8 assay; skin-irritation assessment; H&E histology; granulation-tissue measurement	Cell proliferation >90%; no irritation, inflammatory infiltration, or epidermal thickening	[178]
Gelatin/choline chloride/glycerol/water eutectogel **Application:** Long-term ECG/EEG electrode	**Model:** L929 fibroblasts; rabbits; guinea pigs **Exposure:** Extracts, 24–72 h; rabbit skin, 72 h; guinea-pig applications, 1–14 d	CCK-8 assay; calcein acetoxymethyl/propidium iodide (Calcein-AM/PI) staining; repeated skin-irritation scoring; Magnusson-Kligman sensitization	Cell viability, 92.0–99.8%; irritation score < 0.33; sensitization Grade 0	[149]
PVA/PAAm/lithium chloride/polydopamine hydrogel ± lidocaine hydrochloride **Application:** Pain monitoring; photothermal lidocaine delivery	**Model:** RAW 264.7 macrophages; human skin fibroblasts; in vivo skin-contact sites **Exposure:** Cells, 24 h; skin assessment, 0–120 h	CCK-8 assay; actin/4,6-diamidino-2-phenylindole (DAPI) imaging; tubulin/DAPI imaging; erythema/edema scoring; H&E/toluidine-blue staining; eosinophil/mast-cell counts	Cell viability > 80%; erythema/edema scores, 0; inflammatory-cell counts comparable to control	[184]

### 4.3. Gel–Electrode and Device Interfaces

Whereas the preceding sections focused on electrical, mechanical, and biological interactions at the skin-facing surface, the signals and mechanical loads received by the gel must subsequently be transferred to the solid components of the device. At this device-facing side, stable operation requires both electrical continuity and mechanical integration among the conductive gel, electrode, substrate, interconnects, encapsulation layers, and readout electronics [24,138]. Even when the bulk gel retains sufficient conductivity and deformability, loss of contact at the gel–electrode interface can reduce the electrically active area and increase interfacial resistance or impedance, whereas separation from substrates or encapsulation layers can disrupt gel geometry, mechanical load transfer, or environmental protection. Either form of interfacial failure can eventually cause output drift or device failure [139].

**Figure 11:**
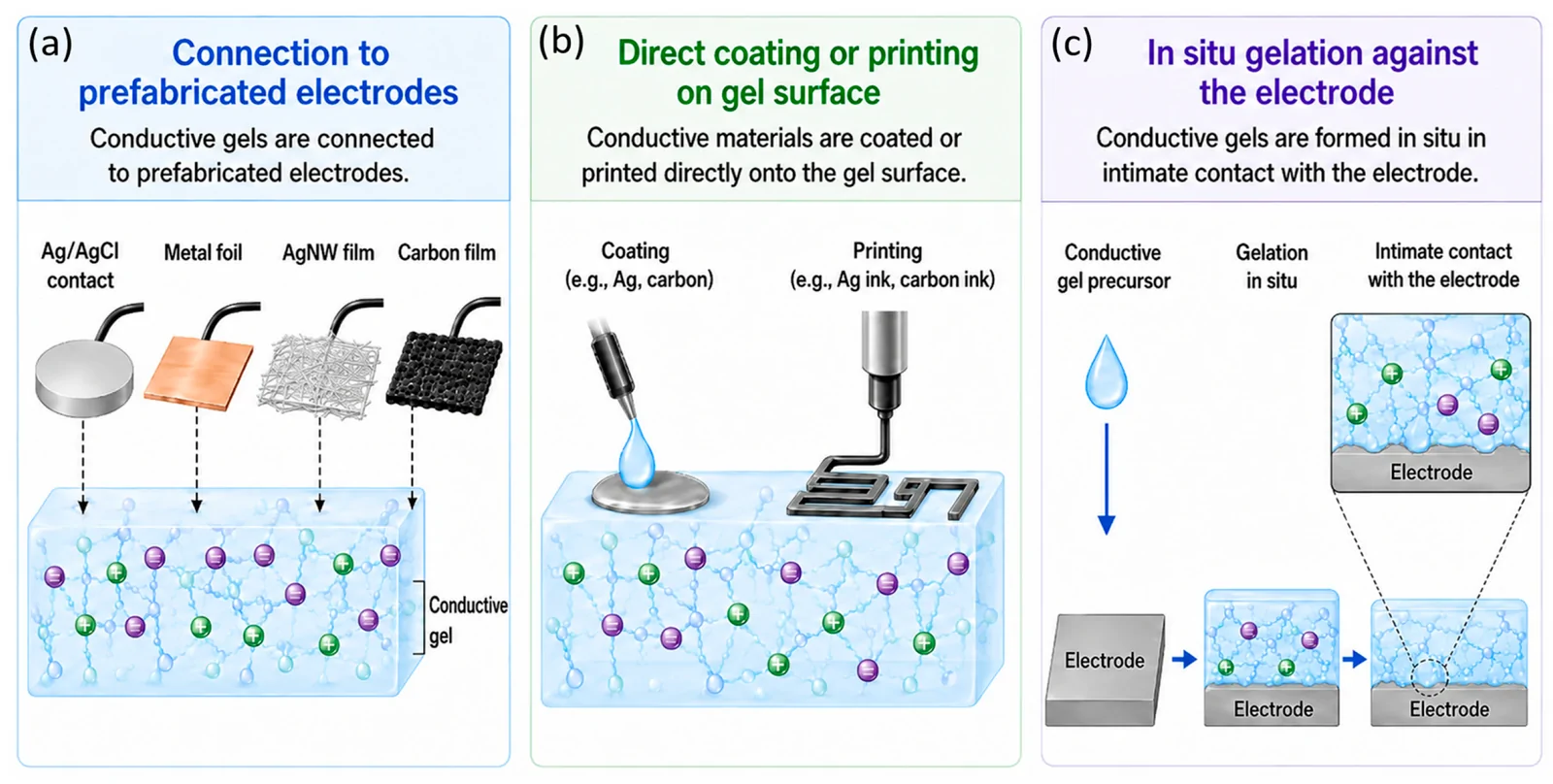
**Representative electrode configurations and joining methods for conductive gel–electrode interfaces.** (**a**) Connection of a conductive gel to prefabricated electrodes, including silver/silver chloride (Ag/AgCl) contacts, metal foils, silver nanowire (AgNW) films, and carbon films. (**b**) Direct coating or printing of conductive materials onto the gel surface. (**c**) In situ gelation of a conductive gel precursor against the electrode to form intimate interfacial contact. The graphical illustrations in the figure were generated using ChatGPT (GPT-5.6, OpenAI), while the overall layout, labels, and annotations were developed by the authors.

As illustrated in Figure 11, conductive gels can be connected to prefabricated electrodes, including silver/silver chloride (Ag/AgCl) contacts, metal foils, AgNW films, and carbon films [4,157]. Alternatively, to reduce the number of separately laminated interfaces, conductive materials can be coated or printed directly onto the gel surface [185], or the gel can be formed in situ against the electrode [176]. These electrode configurations and joining methods can jointly determine the mechanical stability and electrical impedance of the gel–electrode interface.

Maintaining this connection is difficult because a highly deformable and viscoelastic gel is commonly integrated with comparatively stiff electrodes, polymer substrates, connectors, and circuit modules. Table 5 summarizes the mechanical properties of representative conductive gels and device components. These values are grouped by component class to illustrate potential mechanical mismatch during device integration. Differences in testing conditions and material state, including the hydration and solvent content of gels, limit direct quantitative comparisons across studies. Reported fracture strains do not by themselves establish a reversible operating range or durability under cyclic loading. Despite these limitations, the broad differences in mechanical properties can help identify potential mismatch between gels and device components and guide interface design to accommodate deformation. Mechanical mismatch can cause strain localization in the gel near junctions with comparatively stiff electrodes or device supports, where the attached component constrains deformation. Consequently, the local strain can exceed the nominal device strain [186]. At a bonded gel–electrode interface, the associated stress concentration can promote interfacial failure through local debonding and progressive delamination during repeated deformation [187]. Maintaining the connection therefore requires control of local deformation and resistance to interfacial crack growth, in addition to sufficient bulk stretchability of the gel [187,188]. In addition to such mechanical mismatch, incompatibility in surface chemistry between the gel and the electrode or adjacent solid device layers can weaken interfacial bonding. For example, hydrophilic gel layers commonly form only weak physical interactions with hydrophobic elastomers such as PDMS and Ecoflex, and these interactions can deteriorate during repeated or prolonged deformation [25]. Solvent loss or moisture uptake can additionally shrink or swell the gel, thereby changing interfacial stress, contact area, and resistance even without visible failure [31].

**Figure 12:**
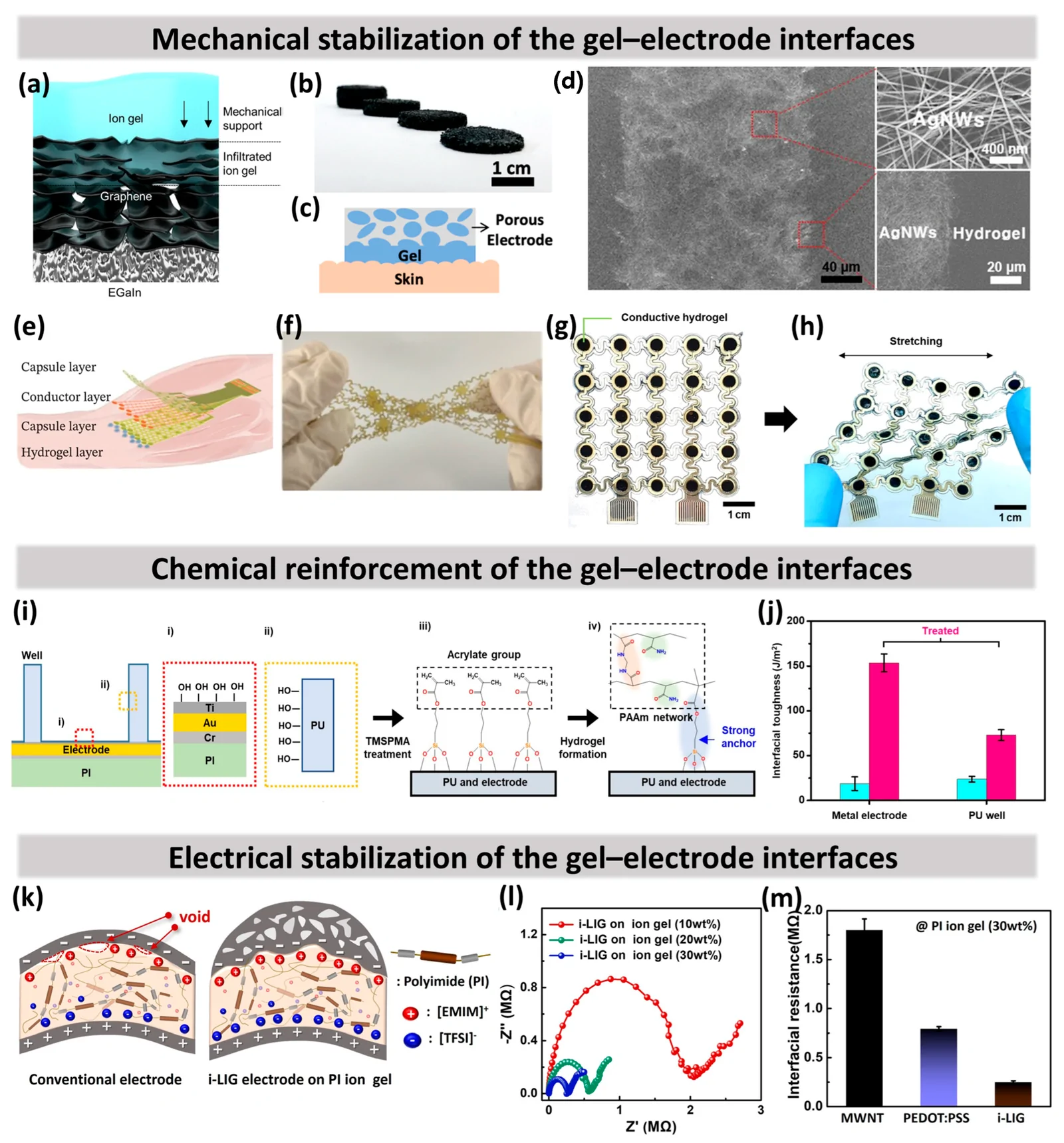
**Representative strategies for mechanical, chemical, and electrical stabilization of gel–electrode interfaces**. (**a**) Partial infiltration of an ionogel through a graphene layer on a eutectic gallium-indium (EGaIn) current collector, mechanically supporting the graphene/EGaIn electrode stack during deformation. Adapted from Kim et al. [192]. © The Author(s) 2024. Published by Springer Nature under a CC BY 4.0 license. (**b**,**c**) Porous sponge electrodes for mechanically compliant electrode-gel coupling. (**b**) Photograph of PEDOT PDMS sponge electrodes. (**c**) Conductive gel infiltration into the porous electrode, increasing the electrode-gel contact area while maintaining compliant contact with the skin. Adapted from Lo et al. [193]. © 2022 The Authors. Published by American Chemical Society under a CC BY 4.0 license. (**d**) Field-emission scanning electron microscopy images of AgNW micropatterns on glass before transfer and on a hydrogel after gelation-assisted transfer, showing preservation and integration of the nanowire network at the gel interface. Adapted from Ko et al. [191]. © The Author(s) 2017. Published by Springer Nature under a CC BY 4.0 license. (**e**,**f**) Mechanically compliant flexible EMG sensor integrating an in situ-gelled hydrogel with a mesh electrode. (**e**) Layered device architecture comprising a copper conductor encapsulated by PI layers and interfaced with the hydrogel. (**f**) Photograph of the fabricated flexible open-mesh electrode patch. Adapted from Zhang et al. [195]. © 2025 The Author(s). Published by UESTC and John Wiley & Sons Australia, Ltd. under a CC BY 4.0 license. (**g**,**h**) Open-mesh sensor array combining serpentine electrode traces with patterned conductive hydrogels in relaxed and stretched states, illustrating geometric accommodation of tensile deformation. (**i**) Chemical integration through surface oxidation, 3-(trimethoxysilyl)propyl methacrylate (TMSPMA) functionalization, and in situ PAAm gelation to anchor the hydrogel to the metal electrode and polyurethane well. (**j**) Interfacial toughness of untreated and chemically treated gel–device interfaces. Adapted from Shin et al. [24]. © 2024 The Authors. Published by the American Association for the Advancement of Science under a CC BY 4.0 license. (**k**–**m**) Electrical stabilization through the direct formation of ionic laser-induced graphene (i-LIG) electrodes on polyimide (PI) ionogels. (**k**) Comparison between a void-containing conventional electrode interface and the conformal i-LIG/PI-ionogel interface. (**l**) Electrochemical impedance spectroscopy Nyquist plots for i-LIG on PI ionogels containing 10–30 wt% ionic liquid. (**m**) Interfacial resistance of a 30 wt% PI ionogel coupled to multiwalled carbon nanotube (CNT), PEDOT:PSS, and i-LIG electrodes. Adapted from Kim et al. [209]. © The Author(s) 2024. Published by Springer Nature under a CC BY 4.0 license.

Accordingly, mechanical stabilization of the gel–electrode interface should address both the deformability of the electrode and the chemistry of its attachment to the gel. The deformability of the electrode can be introduced through the intrinsic properties of the conductive material, rearrangement of the conductive network, or geometric design of the electrode [189,190,191,192,193]. Liquid metals are intrinsically deformable because their fluid phase can change shape and redistribute within a stretchable channel or electrode cavity without brittle fracture, thereby maintaining a continuous conductive path [189]. Conductive polymers can be made more deformable through plasticization or blending with elastomers [190]. AgNWs or graphene flakes can be deposited on or partially embedded within an elastomeric substrate to form overlapping conductive networks that maintain electrical connectivity under deformation. In addition, when a gel precursor infiltrates the voids between the nanowires [191] or graphene flakes [192] and subsequently undergoes in situ polymerization or gelation [193], the electrode network becomes partially embedded within the resulting gel, as shown in Figure 12a–d. This configuration increases the effective gel–electrode interfacial area and creates mechanical interlocking, thereby improving resistance to interfacial sliding and delamination [137,194]. Furthermore, similar physical anchoring can be promoted on other electrodes by introducing micro- or nanoscale roughness, pores, or hierarchical surface structures that allow penetration of the gel precursor [191]. Structural designs provide another route to deformability. Geometrically engineered electrode structures, such as open-mesh networks [195], serpentine traces [24], and wavy traces [196,197], can accommodate extension through straightening, rotation, or out-of-plane bending, as shown in Figure 12e–h. Collectively, these material-, network-, and geometry-based designs accommodate the applied deformation through fluid redistribution, rearrangement of conductive contacts, or geometric changes, thereby reducing the local strain transferred to the gel–electrode interface. Consequently, compared with continuous, unpatterned rigid electrodes, they can help preserve interfacial contact and electrical continuity during deformation [138,190].

**Table 5:** Representative mechanical properties of conductive gels and device components.

Component Class	Representative Material/Component	Young’s/Elastic Modulus	Strain Capacity (Fracture Strain)	Refs
Conductive gel	Self-doped poly(3,4-ethylenedioxythiophene) (S-PEDOT) supramolecular polymer-network hydrogel	10.5 kPa	>1000%	[148]
Conductive gel	On-skin paintable gelatin-based biogel	73.4 ± 2.2 kPa	~400%	[143]
Conductive gel	Polyurethane (PU)-nanomesh-reinforced organohydrogel nanofilm (PU_0_._1_-GHGNT)	1.3 ± 0.1 MPa	166.3%	[68]
Conductive gel	Gelatin-tris(2-hydroxyethyl)methylammonium methylsulfate ([MTEOA][MeOSO_3_]) ionogel	7 kPa	530%	[198]
Conductive gel	Poly[benzyl acrylate-co-poly(ethylene glycol) methyl ether methacrylate] (P(BA-co-PEGMA))/1-ethyl-3-methylimidazolium dicyanamide ([EMIM][DCA]) highly conductive and stretchable nanostructured (CSN) ionogel, 70 wt% ionic liquid	12 kPa	1540%	[199]
Polymer substrate	Ecoflex 00-30, 1:1	0.22 ± 0.02 MPa	502 ± 51%	[200]
Polymer substrate	Sylgard 184 polydimethylsiloxane (PDMS), 10:1	3.9 ± 0.2 MPa	119 ± 9%	[200]
Polymer substrate	Kapton flexible printed circuit (FPC) polyimide film, 25.4–127 μm	2.80 GPa	72–82%	[201]
Polymer substrate	Mylar A polyethylene terephthalate (PET) film, 50–125 μm	3.6–4.1 GPa	120–150%	[202]
Thin metal film	Freestanding Au thin film	53–55 GPa	~1%	[203,204]
Thin metal film	Electroplated Cu thin film, 5.42 ± 0.33 μm	81.48 ± 2.59 GPa	15.0 ± 0.7%	[205]
Solder connector	Bulk tin-silver-copper solder, Sn-3.0Ag-0.5Cu (SAC305)	26.3 GPa	41.0%	[206]
Circuit substrate	G10/flame-retardant grade 4 (FR-4) glass-epoxy laminate, typical	~17.2 GPa	~1%	[207]
Circuit substrate	Single-crystalline Si, ⟨110⟩ direction	169.2 ± 3.5 GPa	~0.35–0.71%	[208]

Chemical interactions can further reinforce this mechanically interlocked interface. Hydroxyl, carboxyl, or amide groups in the gel may form hydrogen bonds with polar or oxidized electrode surfaces [210], catechol groups may form coordination interactions with metals or metal oxides [211], and aromatic groups may interact with carbon-based electrodes through π-π interactions [161]. For more durable attachment, plasma treatment or chemical oxidation can introduce reactive surface groups. Shin et al. monolithically integrated a PEDOT:PSS-PANI-PAAm conductive hydrogel with a Cr/Au/Ti electrode array through on-device in situ polymerization and chemical interfacial anchoring. The exposed Ti electrodes and surrounding PU wells were oxidized by O_2_ plasma and functionalized with a self-assembled monolayer of 3-(trimethoxysilyl)propyl methacrylate (TMSPMA). During ultraviolet-induced polymerization, the methacrylate groups of TMSPMA crosslinked with the PAAm network and covalently anchored the hydrogel to the electrodes and PU wells (Figure 12i). In 90° peel tests, this functionalization increased the hydrogel–electrode interfacial toughness from 17.3 to 153.6 J∙m^−2^ (Figure 12j). Subsequent TA treatment increased it to approximately 297 J∙m^−2^ through hydrogen-bond-mediated energy dissipation. The integrated array could be stretched to 100%. Although its impedance increased at 50% and 100% strain, it returned to the initial level after strain release in the reported stretching-cycle test [24]. These approaches reinforce load transfer and suppress interfacial sliding or delamination without necessarily eliminating the underlying mechanical mismatch.

Mechanical integrity alone, however, does not ensure electrical continuity. Therefore, the electrical quality of the gel–electrode interface should be considered separately from the bulk conductivity of the gel. The origin of the interfacial impedance depends on the charge-coupling mechanism. At an ionically conductive gel–electrode interface, mobile ions redistribute in response to electrode surface charge. The resulting EDL introduces a capacitive and frequency-dependent contribution to the interfacial impedance [209,212]. Charging and discharging of this layer provide non-Faradaic capacitive coupling without electron transfer across the interface. In the ideal capacitive limit, the impedance magnitude associated with EDL charging decreases with increasing frequency. Faradaic charge transfer can also occur through interfacial redox reactions, depending on factors such as electrode chemistry, electrolyte composition, and operating potential [212]. In contrast, at an electronically conductive gel–electrode interface, the interfacial contribution is primarily associated with electronic contact resistance when the conductive network within the gel directly contacts the current collector [213]. Mixed ionic–electronic conducting gels may exhibit a combination of these responses [146]. Although the ionic and electronic contributions have different physical origins, both depend on the electrically active contact between the gel and the electrode. The contact area defines the region available for electric-double-layer formation at ionic interfaces and for carrier transfer at electronic interfaces, while the electrode surface chemistry influences gel wetting and the local charge-coupling conditions [20,73]. Accordingly, the strategies described above for increasing and stabilizing the effective contact area and controlling the electrode surface chemistry can support electrical continuity as well as mechanical attachment.

Assessing how effectively these strategies maintain electrical continuity requires consideration of the frequency-dependent interfacial response, because resistive and capacitive components contribute differently to the measured impedance as a function of frequency [212]. Therefore, both the impedance magnitude and phase should be evaluated over the frequency range relevant to device operation. Electrochemical impedance spectroscopy (EIS) provides one method for such characterization. For example, Kim et al. fabricated a vertical ionotronic capacitor by directly converting the surfaces of a PI ionogel into porous ionic laser-induced graphene (i-LIG) electrodes, forming conformal and void-free gel–electrode interfaces (Figure 12k). These interfaces were evaluated by EIS from 0.1 Hz to 1 MHz. Increasing the ionic-liquid (IL) content from 10 to 30 wt% progressively reduced the Nyquist semicircle size, indicating lower interfacial resistance (Figure 12l). At 30 wt% IL, the i-LIG/PI-ionogel interface exhibited the lowest interfacial resistance of 0.3 MΩ compared with spray-coated multiwalled carbon nanotube (MWNT) and PEDOT:PSS electrodes (Figure 12m). The i-LIG interface also exhibited the highest electric-double-layer capacitance among the tested electrodes, reaching 8 μF∙cm^−2^. This high capacitance was attributed to the low interfacial resistance and large interfacial area produced by directly forming porous i-LIG electrodes on the ionogel [209].

Beyond such comparisons among electrode systems, EIS spectra can be further interpreted using physically justified equivalent-circuit models, as illustrated schematically in Figure 9. The circuits in Figure 9 are illustrative examples constructed using established resistive and capacitive representations of gel conduction and skin-electrode interfaces [140,146,212]. They assume small-signal conditions, ohmic bulk and contact resistances, and idealized capacitive behavior at electrochemical interfaces. The depicted conducting phases are assumed to be accessible at the relevant interfaces. For Figure 9c, mixed conduction is approximated by parallel ionic and electronic pathways without explicit distributed ionic-electronic coupling. These equivalent-circuit models can be used to estimate parameters associated with different components, such as ohmic resistance, charge-transfer resistance, and capacitive parameters. Circuit selection should account for factors such as the gel’s charge-transport pathways, electrode chemistry, and measurement configuration [212]. For example, Mech-Dorosz et al. used equivalent-circuit fitting to analyze hydrogel–electrode impedance over 0.1 Hz–1 MHz. The model included a parallel RC element for bulk gel resistance and capacitance, an interfacial charge-transfer resistance, and a constant phase element (CPE) for nonideal double-layer charging. A finite-length Warburg element and an additional CPE accounted for low-frequency mass transport, allowing bulk and interfacial contributions to be evaluated separately. After 24 h of electrolyte conditioning, increasing the crosslinker/monomer molar ratio from 1:200 to 1:100 reduced the fitted bulk resistance from 3.5–5.8 to 0.20–0.23 kΩ and the charge-transfer resistance from 85–105 to 0.9–16 kΩ, accompanied by lower diffusion impedance [144]. This result illustrates how equivalent-circuit analysis can distinguish the bulk and interfacial contributions to impedance reduction following changes in gel composition. When applying equivalent-circuit analysis to skin-contact systems, the model should also account for the skin response and relevant contact interfaces according to the measurement configuration [140]. A suitable model should reproduce both impedance magnitude and phase over the relevant frequency range using circuit elements with physically justified interpretations [212].

However, minimizing interfacial impedance is not a universal design objective. The required interfacial response depends on the device function. Low and stable impedance is desirable for signal-transmission interfaces, whereas controlled and reproducible changes in interfacial impedance or capacitance may serve as sensing signals in ionotronic and electrochemical sensors. For signal transmission requiring low impedance at low frequencies, Sekitani et al. developed a conductive gel electrode coupled to an ultraflexible organic amplifier to record and amplify weak epicardial ECG signals directly from the surface of a rat heart. The electrode combined a multiwalled CNT sheet with an aqueous polyrotaxane-based gel, and the large effective area of the CNT/gel interface produced an electric-double-layer capacitance exceeding 140 μF∙cm^−2^. Consequently, the composite maintained an areal admittance of approximately 100 mS∙cm^−2^ even in the low-frequency range, two to three orders of magnitude higher than those of metal/gel composite electrodes, while the integrated system amplified a 1.2 mV cardiac signal to 220 mV with a gain of approximately 200 [214]. By contrast, Bai et al. developed an ionotronic pressure sensor to detect pressure changes ranging from arterial pulses to small load variations beneath a car tire, using reproducible interfacial capacitance as the sensing parameter. Applied pressure progressively increased the contact area between a graded intrafillable architecture (GIA)-based PVA/H_3_PO_4_ ionic film and Au electrodes and the number of electric-double-layer capacitors formed in parallel, producing a pressure-dependent interfacial capacitance response over a pressure range up to 360 kPa. This capacitance response showed no signal drift or fluctuation during 5000 compression-release cycles at 300 kPa, demonstrating its reproducibility for ionotronic sensing [73].

**Figure 13:**
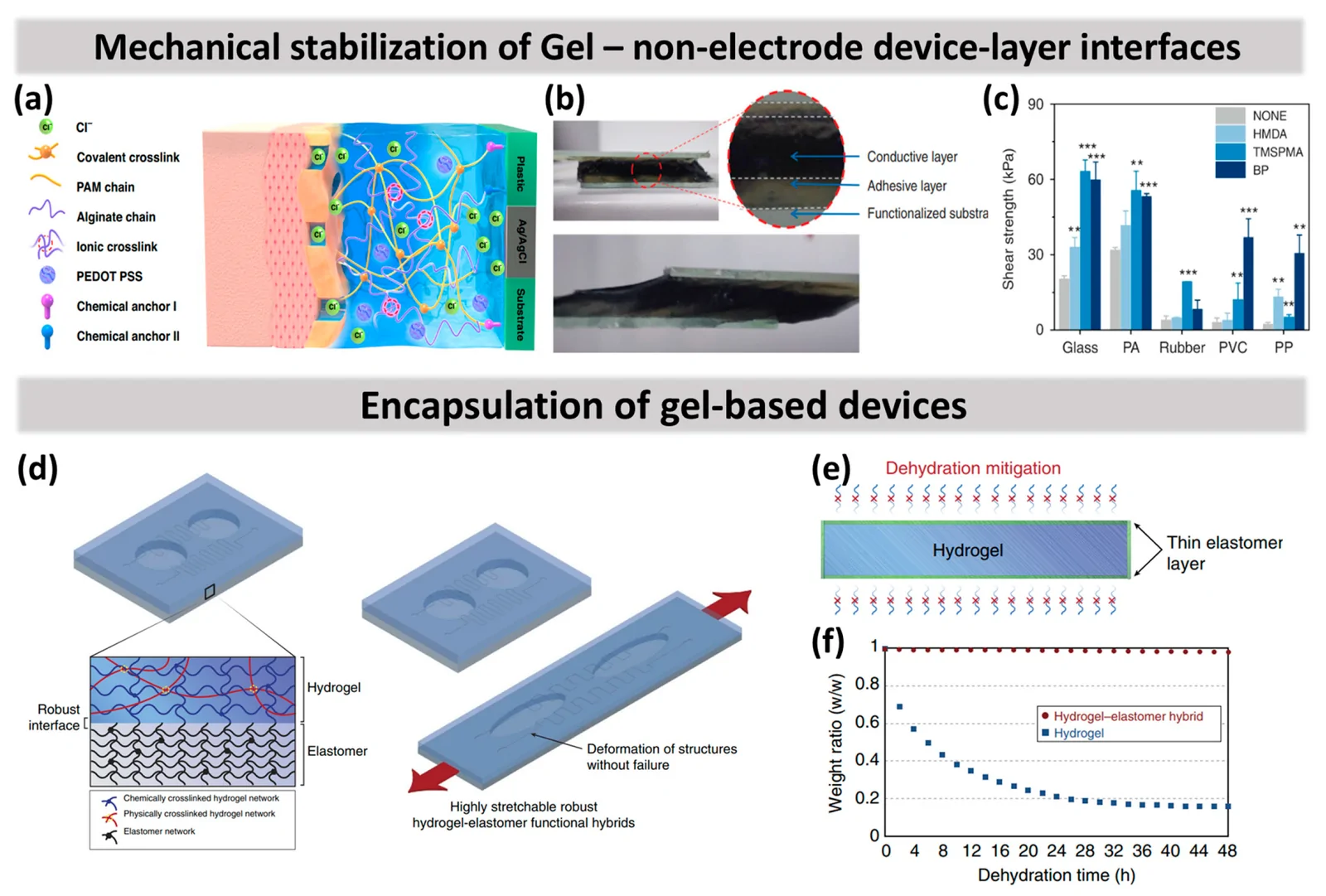
**Representative strategies for mechanical stabilization of gel interfaces with non-electrode device layers and for encapsulation of gel-based devices.** (**a**–**c**) Mechanical stabilization of a conductive-adhesive double-layer hydrogel electrode on solid supporting substrates through chemical anchors. (**a**) Molecular and interfacial design of the hydrogel bilayer, comprising covalently and ionically crosslinked networks and chemical anchoring of the adhesive layer to the substrate. (**b**) Photographs of the integrated conductive and adhesive hydrogel layers bonded to a functionalized substrate, with an enlarged view of the layer interfaces. (**c**) Shear strength of the adhesive hydrogel on glass, polyamide (PA), rubber, poly(vinyl chloride) (PVC), and polypropylene (PP) without surface treatment and after functionalization with 1,6-hexamethylenediamine (HMDA), 3-(trimethoxysilyl)propyl methacrylate (TMSPMA), or benzophenone (BP), ** and *** indicate statistical significance at *p* < 0.01 and *p* < 0.001, respectively. Adapted from Xue et al. [157]. © The Author(s) 2023. Published by Springer Nature under a CC BY 4.0 license. (d-f) Robust hydrogel-elastomer hybrids for device integration and encapsulation. (**d**) Schematic of a covalently anchored hydrogel-elastomer interface that preserves patterned microstructures and accommodates large deformation without interfacial failure. (**e**) Thin Ecoflex elastomer coating robustly bonded to the hydrogel to suppress water evaporation. (**f**) Weight retention of the Ecoflex-encapsulated hydrogel-elastomer hybrid and an uncoated hydrogel during dehydration under ambient conditions (24 °C and 50% relative humidity). Adapted from Yuk et al. [25]. © 2016 Nature Publishing Group, a division of Macmillan Publishers Limited. Published under a CC BY 4.0 license.

Interfaces between the gel and adjacent non-electrode device layers, such as polymer substrates, dielectric or triboelectric sensing layers, microfluidic layers, and structural supports, should be considered separately from the gel–electrode interface. Although some of these layers may participate directly in sensing, their gel-facing interfaces commonly help retain the position and geometry of the gel and transmit or isolate mechanical loads as required [157]. Maintaining these functions requires sufficiently stable integration to suppress relative sliding, separation, and delamination between the gel and the adjacent layer during device operation [215]. The chemical reinforcement strategies described above for strengthening gel–electrode interfaces can likewise be applied to gel interfaces with non-electrode device layers, although the functionalization chemistry should be matched to the adjoining substrate. For example, in a semidry double-layer hydrogel EEG electrode, as shown in Figure 13a,b, Xue et al. stabilized the interface between a substrate-adhesive hydrogel layer and various non-electrode solid supports. Before bonding, glass, polyamide (PA), rubber, poly(vinyl chloride) (PVC), and polypropylene (PP) substrates were activated by oxygen plasma and subsequently functionalized with 1,6-hexamethylenediamine (HMDA), TMSPMA, or benzophenone (BP), thereby forming chemical anchors between the adhesive hydrogel and the substrate surfaces. As shown in Figure 13c, lap-shear tests demonstrated that surface functionalization increased the gel-substrate lap-shear strength relative to untreated interfaces, although the extent of enhancement depended on the substrate material and functionalization chemistry. Moreover, for selected glass, PA, and PVC substrates, the shear strength did not decrease from day 1 to day 14 after curing, supporting the time stability of bonding between the hydrogel and the non-electrode supports [157]. The reported retention of shear strength from day 1 to day 14 supports stability under the tested conditions, but does not by itself establish long-term durability under combined hydration and cyclic loading. More generally, covalent anchoring through TMSPMA should be evaluated together with the fatigue resistance of the adjacent gel network. For example, Liu et al. reported that a model PAAm-alginate hydrogel bonded to TMSPMA-functionalized glass exhibited a single-cycle interfacial toughness of approximately 1500 J∙m^−2^, whereas its interfacial fatigue threshold was 68 J∙m^−2^ under aqueous testing [216]. Long-term assessment should therefore track adhesion retention, crack growth, and electrical performance under application-relevant environmental conditions and repeated deformation.

Beyond chemical anchoring, compliant or modulus-graded interlayers can reduce abrupt changes in effective stiffness [10,186], whereas localized or patterned bonding, mechanical anchoring, and spatial separation from rigid components can distribute deformation away from vulnerable junctions [138]. Thus, the mechanical stabilization of gel interfaces with non-electrode device layers requires coordinated control of interfacial bonding, stiffness transitions, and structural load-transfer pathways.

Encapsulation represents an additional device-side interface because it must provide mechanical protection while regulating solvent and moisture transport. Evaporation can dehydrate and shrink aqueous gels, whereas humidity, sweat, or immersion can cause water uptake, swelling, and leakage of ionic or conductive components; these changes can alter gel geometry, conductivity, and interfacial contact [31]. To mitigate these environmentally induced changes, elastomeric barriers with sufficiently low solvent permeability can restrict mass exchange and thereby improve solvent retention. For example, Hou et al. reported that a CaCl_2_-containing MXene composite hydrogel exhibited a water loss of 16.5% after 3 days at 25 °C and 45% relative humidity, whereas encapsulation with Ecoflex reduced the water loss to 1.4% [217].

Beyond limiting solvent loss, effective encapsulation also requires stable adhesion at the gel-encapsulant interface under mechanical deformation and hydration-induced changes in gel volume. Yuk et al. addressed this requirement by using benzophenone-mediated ultraviolet grafting to covalently bond a tough PAAm-alginate hydrogel to Ecoflex (Figure 13d). An approximately 100-μm-thick Ecoflex coating (Figure 13e) prevented noticeable hydrogel weight change over 48 h at 24 °C and 50% relative humidity (Figure 13f). The PAAm-alginate/Ecoflex interface exhibited an interfacial toughness of 1580 J∙m^−2^ in the as-prepared state and 1044 J∙m^−2^ after equilibrium swelling in deionized water for more than 24 h. Failure occurred cohesively within the hydrogel rather than through interfacial separation. A laminate bonded using the same strategy stretched to approximately seven times its original length without delamination. Because of its small thickness, low modulus of approximately 30 kPa, and high stretchability, the coating did not significantly alter the overall mechanical properties of the hydrogel [25]. These results illustrate that an encapsulation layer should function not only as a solvent barrier but also as a compliant and strongly bonded interface. However, these protective functions of encapsulation must be balanced against the mechanical and mass-transport requirements of the sensing mechanism. Dense or mechanically stiff encapsulation can reduce device stretchability and sensitivity, restrict water-vapor transport, or prevent the exposure required for chemical and biochemical sensing [218,219]. Accordingly, the encapsulant material, thickness, permeability, adhesion, and coverage should be selected according to the intended sensing mechanism.

Evaluation of gel–electrode and device interfaces should extend beyond measurements of the isolated gel. Assessments should be complemented by tests on the complete assembly because stable bulk-gel properties do not necessarily ensure reliable bonding or electrical continuity across heterogeneous device interfaces [220,221]. Therefore, coordinated consideration of interfacial and device-level factors, such as electrode materials, compliant interconnects, interfacial bonding, encapsulation, and moisture transport, is required to ensure reliable long-term operation of wearable and attachable devices.

## 5. Applications of Conductive Gels in Wearable and Attachable Healthcare Devices

This section examines how conductive gels are implemented in wearable and attachable healthcare devices. Depending on the device architecture, conductive gels can function as conformal bioelectrodes for electrophysiological recording, deformable sensing elements for strain and pressure, thermal- or moisture-responsive layers, or analyte-permeable matrices for chemical and biochemical detection [222]. Their practical performance, however, is determined at the level of the complete device because gel composition and network structure operate together with skin–gel coupling, gel–electrode integration, solvent and ion retention, and mechanical and environmental stability [223]. The principal performance requirements therefore vary with the target application. Electrophysiological monitoring requires low and stable interfacial impedance and suppression of motion artifacts [182,224]; motion monitoring requires an appropriate sensing range, low hysteresis, and repeatable responses under cyclic deformation [225]; and biochemical monitoring requires selective analyte recognition and transport together with calibration stability under changes in hydration, temperature, and mechanical deformation [226,227]. On this basis, representative applications are organized according to the physiological function or healthcare task addressed: (i) cardiovascular and respiratory monitoring, (ii) musculoskeletal motion and muscle-activity monitoring, (iii) neurological and ocular bioelectrical monitoring, and (iv) biochemical and cutaneous health monitoring.

### 5.1. Cardiovascular and Respiratory Monitoring

Cardiovascular and respiratory monitoring applications illustrate the different roles that conductive gels can perform within soft, body-conformal healthcare devices. For cardiovascular assessment, a gel may serve as an epidermal or tissue-contacting electrode that couples cardiac electrical activity to recording electronics or as a deformable sensing element that responds to arterial motion [228,229]. Respiratory devices can instead follow expansion of the chest and abdomen or detect pressure, temperature, and humidity changes associated with airflow near the nose or mouth [230]. These measurement routes provide complementary information on cardiac rhythm, vascular pulsation, and ventilation.

**Figure 14:**
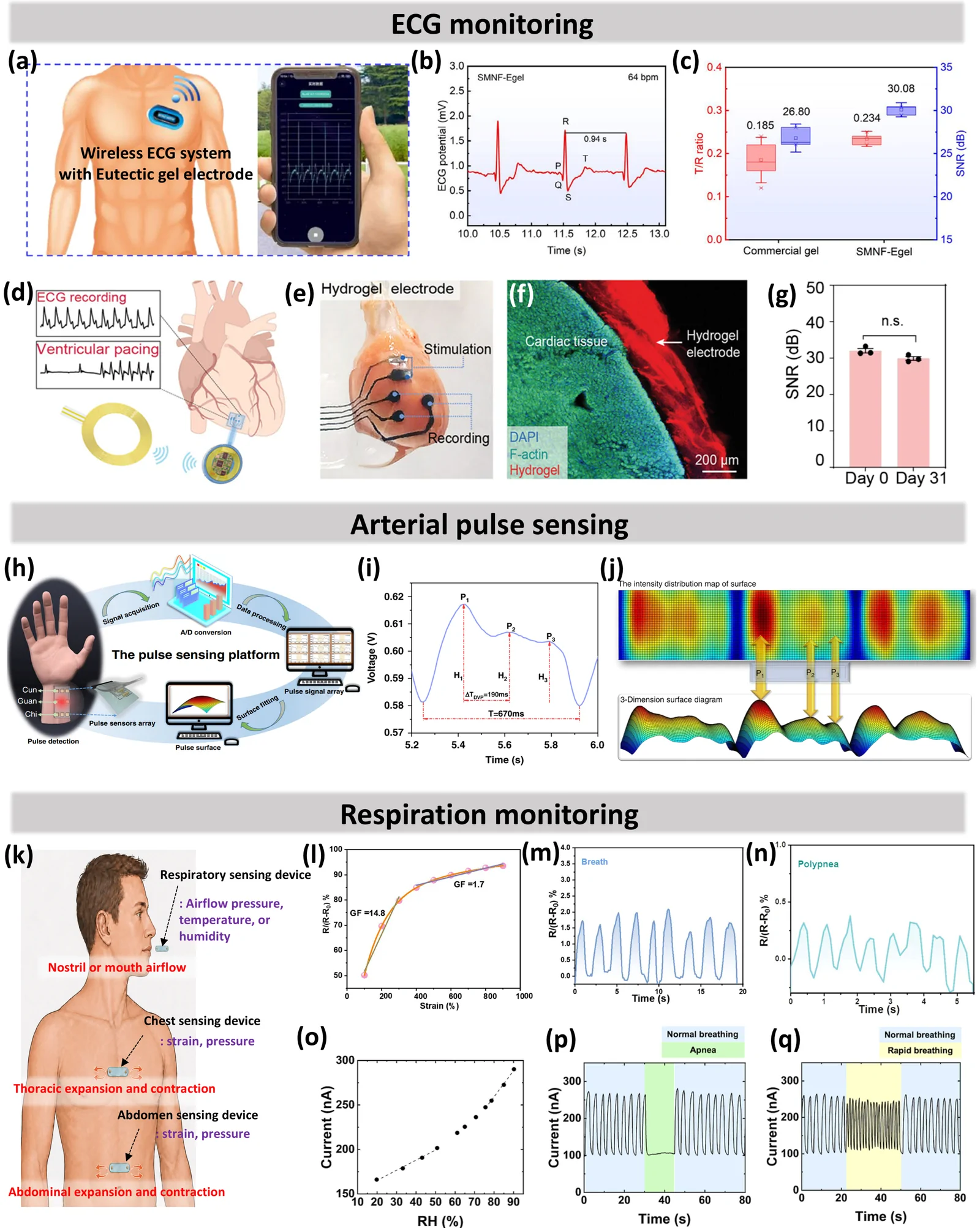
**Representative cardiovascular and respiratory applications of conductive gels for ECG monitoring, arterial pulse sensing, and respiration monitoring.** (**a**–**c**) Wearable electrocardiogram (ECG) monitoring using silk micro/nanofibril-reinforced eutectogel (SMNF-Egel) epidermal electrodes. (**a**) Wireless ECG system with chest-mounted SMNF-Egel electrodes and smartphone readout. (**b**) ECG traces showing the P wave, QRS complex, and T wave. (**c**) Comparison of the T/R ratio and signal-to-noise ratio (SNR) between commercial gel and SMNF-Egel electrodes. Adapted from Yang et al. [233]. © 2026 The Author(s). Published by Wiley-VCH GmbH under a CC BY 4.0 license. (**d**–**g**) Wireless implantable cardiac sensing and pacing using conductive hydrogel electrodes. (**d**) Schematic of a wireless sensing and pacing device (WSPD) configured for ECG recording and ventricular pacing of an ex vivo heart. (**e**) Hydrogel electrodes conformally attached to an ex vivo rat heart for electrical stimulation and recording. (**f**) Confocal image of the interface between the hydrogel electrode and cardiac tissue. (**g**) Comparison of the SNR values of ECG signals recorded on Day 0 and Day 31 after implantation. Adapted from Chang et al. [235]. © 2024 The Author(s). Published by Wiley-VCH GmbH under a CC BY 4.0 license. (**h**–**j**) Wearable multichannel arterial-pulse sensing using an ionogel-based pressure-sensor array. (**h**) Schematic of the wrist-worn pulse-sensing platform incorporating a 3 × 3 sensor array at the Cun, Guan, and Chi positions. (**i**) Representative radial-pulse waveform showing the early systolic peak (P1), inflection point (P2), and dicrotic peak (P3). (**j**) Time-resolved pulse-intensity maps and corresponding three-dimensional surface profiles across one pulse cycle, including P1, P2, and P3. Adapted from Wang et al. [236]. © The Author(s) 2022. Published by Springer Nature under a CC BY 4.0 license. (**k**) Schematic of two respiratory-monitoring approaches: detection of airflow-related pressure, temperature, or humidity changes near the nose or mouth and detection of thoracoabdominal strain or pressure generated by chest and abdominal motion. The graphical illustration of the human upper body was generated using ChatGPT (GPT-5.6, OpenAI). (**l**–**n**) Hydrogel strain sensing of respiration-induced abdominal deformation. (**l**) Relative resistance change as a function of tensile strain. (**m**,**n**) Relative resistance responses during (**m**) normal breathing and (**n**) polypnea. Adapted from Zhao et al. [239]. © 2025 by the authors. Published by MDPI under a CC BY 4.0 license. (**o**–**q**) Humidity-based respiratory monitoring using a mask-integrated ionic-liquid-gel device. (**o**) Sensor current as a function of relative humidity. (**p**,**q**) Current responses during normal breathing interrupted by (**p**) simulated apnea and (**q**) rapid breathing. Adapted from Qing et al. [240]. © 2026 American Chemical Society. Published under a CC BY 4.0 license.

ECG records electrical activity generated by cardiac muscle, and its waveform commonly contains the P wave, QRS complex, and T wave. In wearable devices, conductive gels are primarily employed as compliant epidermal electrodes that maintain stable electrical contact with the skin at ECG measurement sites, such as the chest, wrist, forearm, upper arm, and fingers, during body motion [231]. Their conformability and adhesion can reduce motion artifacts and support stable signal acquisition [232], while integration with compact electronics enables continuous and wireless recording. For example, Yang et al. developed a wearable ECG platform using adhesive conductive eutectogel electrodes comprising a silk micro/nanofibril-reinforced poly(acrylic acid)/choline chloride matrix and eutectic gallium-indium (EGaIn) microdroplets (Figure 14a). After integration with metal electrode buttons, the bioelectrodes exhibited a lap-shear adhesion strength of 77.5 kPa on pigskin and an interfacial impedance of 299 Ω at 100 Hz, compared with 1362 Ω for commercial gel electrodes. When placed on the chest and connected to a wireless ECG system with smartphone readout, the electrodes clearly resolved the P wave, QRS complex, and T wave (Figure 14b). Compared with commercial electrodes, they produced a higher T/R ratio of 0.234 versus 0.185 and SNR of 30.08 versus 26.80 dB, indicating a more prominent T-wave component relative to the R peak and improved signal fidelity (Figure 14c). Continuous recording during 1 h of computer work, walking, resting, and laboratory activity further demonstrated ECG monitoring under static and dynamic daily conditions. In a separate strain-sensing test, the same eutectogel maintained stable relative-resistance responses during 1000 stretch-release cycles at 100% strain, with slight baseline drift attributed to mechanical hysteresis [233]. This example demonstrates how the skin–gel interface and device-level integration contribute to reliable, noninvasive cardiac electrical monitoring.

Beyond epidermal monitoring, conductive gels can also serve as compliant electrode interfaces in implantable, tissue-attachable cardiac devices [234]. When placed directly on the heart surface, the gel can accommodate cardiac motion while providing electrical coupling for local cardiac signal recording and, in some device configurations, electrical stimulation such as pacing. For example, Chang et al. developed a fully implantable wireless cardiac pacing and sensing system using photopatterned conductive hydrogel electrodes comprising PEDOT:PSS, crosslinked zwitterionic sulfobetaine methacrylate (SBMA), and the ionic additive neostigmine methanesulphate (NM) (Figure 14d). With a conductivity of 160 S∙m^−1^, a Young’s modulus of 80 kPa, and stretchability up to 270%, the electrodes conformed to the moving cardiac surface (Figure 14e and the confocal image in Figure 14f). Attached to the right ventricular surface and connected through flexible Au interconnects to a subcutaneously implanted, wirelessly powered module, they enabled ECG recording and externally programmable cardiac stimulation. After 31 days of implantation in rats, the electrodes maintained ECG recordings with a consistent SNR (Figure 14g), while cardiac pacing increased the heart rate from 300 to 600 bpm [235]. This example represents how conductive gels can extend cardiac electrical monitoring from noninvasive skin-mounted systems to implantable, tissue-attachable devices that maintain direct sensing and stimulation interfaces with beating cardiac tissue. These applications demonstrate that conductive gels can support cardiac electrical monitoring in both noninvasive wearable devices and devices attached directly to cardiac tissue.

Whereas these electrode-based configurations monitor cardiac electrical activity, arterial pulse sensing follows a different signal pathway by detecting the small periodic deformation transmitted from a superficial artery to the skin [229]. A gel-based pressure sensor placed over the radial artery can convert this motion into resistance changes, enabling the analysis of pulse frequency, the timing between characteristic waveform features, and waveform morphology. Wang et al. developed a wearable multichannel radial-pulse monitoring system using a 3 × 3 ionogel-based resistive pressure-sensor array aligned with the Cun, Guan, and Chi positions on the wrist (Figure 14h). Pulse-induced deformation of the ionogel elements produced resistance changes that resolved the early systolic peak (P1), inflection point (P2), and dicrotic peak (P3) (Figure 14i). The nine-channel signals were reconstructed into three-dimensional maps of pulse strength, length, and width (Figure 14j), providing waveform-derived and spatial information beyond pulse-rate counting [236].

The cardiovascular applications discussed above illustrate how conductive gels can serve as compliant bioelectrodes for ECG recording and mechanical sensing elements for arterial pulse sensing. The electrical origin of ECG and the mechanical origin of arterial pulse signals impose different requirements on bandwidth, noise control, and gel-tissue coupling. For example, adult diagnostic surface ECG commonly uses approximately 0.05–150 Hz [237], whereas the pulse system described above focuses on waveform components below 20 Hz [236]. These differences require gel properties and interfacial conditions to be tailored to each sensing mode to maintain signal fidelity. For ECG, stable gel conduction and conformal contact help maintain low interfacial impedance and limit contact-related motion artifacts, while muscle and power line interference also require consideration in the recording system [68,155,237]. For arterial pulse sensing, sensitivity to small pressure changes, limited hysteresis, and adequate dynamic response help preserve waveform features, with controlled sensor alignment and attachment pressure helping to limit mechanical artifacts [229,236,238]. Accordingly, the reliable use of conductive gels in cardiovascular applications depends on maintaining signal quality and interfacial stability during motion and prolonged wear.

As illustrated in Figure 14k, respiration can be assessed through two distinct sensing approaches based on thoracoabdominal motion and airflow-related changes. A gel strain or pressure sensor attached to the chest or abdomen can follow cyclic expansion and contraction and convert these movements into electrical signals reflecting respiratory behavior. For instance, Zhao et al. used a self-adhesive PAAm-based conductive hydrogel containing MXene and montmorillonite as a resistive strain sensor. It exhibited a gauge factor of 14.8 over 0–300% strain and response and recovery times of 80 and 110 ms, respectively (Figure 14l). When attached to the abdomen of a volunteer, respiratory deformation produced cyclic relative-resistance changes (Figure 14m) and distinguishable waveforms for normal breathing, rapid breathing or polypnea (Figure 14n), and deep breathing, reflecting differences in breathing frequency and relative intensity [239]. This example demonstrates how body-surface deformation is used to monitor respiratory mechanics.

A second approach detects airflow near the respiratory tract. A conductive gel device positioned near the nose or mouth can respond to airflow-induced pressure and temperature changes or to differences in humidity between inhaled and exhaled air. For example, Qing et al. integrated an ionic-liquid gel composed of P(VDF-HFP) and [EMIM][TFSI] with screen-printed Ag/AgCl electrodes on the microfibrous polypropylene filter layer of a face mask. The mask-integrated sensor exhibited humidity sensitivities of 1.1 nA/RH% below 60% RH and 2.9 nA/RH% above 70% RH, with initial response and recovery times of 1.1 ± 0.1 and 3.6 ± 0.6 s, respectively (Figure 14o). Breathing-induced humidity changes increased the current during exhalation and decreased it during inhalation. The sensor continuously distinguished normal and rapid breathing at 18 and 40 breaths per min, respectively, while periodic current oscillations disappeared during voluntary simulated apnea (Figure 14p,q) [240]. Although these two approaches detect different physical manifestations of breathing, both can provide information on respiratory rate, relative amplitude, rhythm, and breathing interruptions. Together, these approaches illustrate two distinct routes for characterizing respiratory patterns through body-mounted deformation sensing and airflow-responsive sensing.

In these respiratory applications, conductive gels serve as deformable strain- or pressure-sensing elements and humidity-responsive layers that convert breathing-related changes into electrical signals [239,240]. Because these approaches respond to different physical stimuli, their reliability depends on controlling the sources of interference relevant to each sensing mode. For thoracoabdominal sensing, reproducible placement, stable mechanical coupling, and repeatable responses under cyclic deformation are important, together with separation of respiratory deformation from other body movements [239,241]. For humidity-based sensing, consistent positioning near the nose or mouth and evaluation of ambient temperature and humidity effects are important, while sensor response and recovery times affect the ability to resolve successive breaths [240]. Since the recorded signals represent local deformation or humidity changes, their use to estimate respiratory volume or airflow requires calibration and validation against a suitable reference, such as spirometry, under the intended measurement conditions [241]. Also, changes in gel hydration or sensor attachment can affect the sensing response over time, making evaluation of SNR, baseline drift, and sensitivity important for assessing signal quality and calibration stability during prolonged use.

Accordingly, performance requirements should be matched to the physiological signal pathway: cardiac electrical monitoring requires stable electrical contact at either the skin or cardiac-tissue interface and adequate signal quality during body or cardiac motion [242,243]; arterial-pulse monitoring requires faithful detection of weak pressure waveforms [229]; and respiratory monitoring requires reliable transduction of either thoracoabdominal deformation or airflow-related humidity, temperature, or pressure changes [74,244,245]. Conductive gels can support each route, but device architecture and validation metrics should be selected for the intended physiological signal rather than inferred from bulk gel properties alone.

### 5.2. Musculoskeletal Motion and Muscle Activity Monitoring

Musculoskeletal function can be monitored through two complementary signal pathways: mechanical deformation and loading associated with body movement, and the bioelectrical activity generated by skeletal muscle [246]. Conductive gels can serve as deformable strain- or pressure-sensing elements for the first pathway and as compliant epidermal electrodes for EMG in the second [247,248]. These roles enable the monitoring of joint motion, posture, plantar loading, gait, and muscle activity.

**Figure 15:**
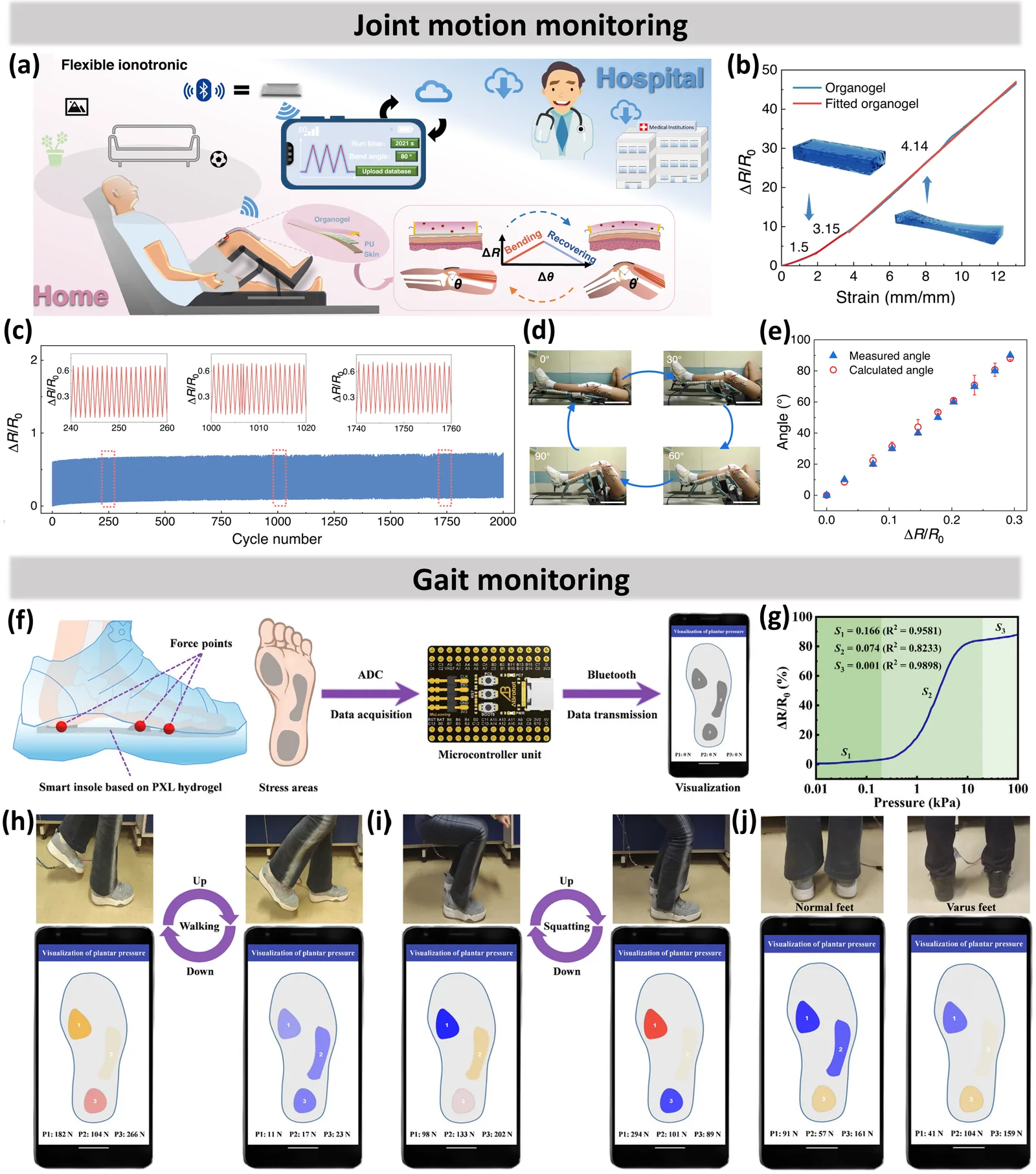
**Representative musculoskeletal applications of conductive gels for joint-motion and gait monitoring.** (**a**–**e**) Organogel-based flexible sensor integrated with an intelligent continuous passive motion (CPM) system for knee rehabilitation. (**a**) Schematic of the at-home CPM and telemedicine platform, combining a flexible sensor, wireless transmission, mobile display, and remote clinical access, together with the resistance-based sensing mechanism during joint bending and recovery. (**b**) Relative resistance change (ΔR/R_0_) of the organogel sensor as a function of tensile strain, showing three strain regimes with fitted gauge factors. (**c**) Relative-resistance response during 2000 successive stretching-releasing cycles at 60% strain, with enlarged views of representative cycle intervals. (**d**) Photographs of CPM-assisted knee flexion at 0°, 30°, 60°, and 90°. (**e**) Comparison of measured knee-flexion angles with values calculated from ΔR/R_0_. Adapted from Tang et al. [42]. © The Author(s) 2023. Published by Springer Nature under a CC BY 4.0 license. (**f**–**j**) Plantar-pressure monitoring using a three-point smart insole based on Li/Al layered double hydroxide (Li/Al-LDH)-reinforced PAAm/xanthan gum semi-interpenetrating-network (PXL) hydrogel sensors. (**f**) System architecture comprising the PXL sensor locations, analog-to-digital conversion, microcontroller-based data processing, Bluetooth transmission, and mobile visualization. (**g**) Relative resistance change over a pressure range of 0.01–100 kPa, showing three pressure-sensitivity regimes. (**h**) Real-time plantar-pressure distributions during foot lowering and lifting in walking. (**i**) Plantar-pressure distributions during squatting down and standing up. (**j**) Distinct regional pressure distributions for normal and varus foot types. Adapted from Hu et al. [252]. © 2025 The Author(s). Advanced Science published by Wiley-VCH GmbH under a CC BY 4.0 license.

Joint motion is commonly assessed by positioning a conductive gel-based strain sensor over or adjacent to a finger, wrist, elbow, knee, or neck joint. Flexion and extension deform the gel and alter its geometry or conductive pathways, resulting in changes in resistance, capacitance, or generated voltage depending on the device architecture [246]. With appropriate placement and calibration, these outputs can distinguish bending angles, movement velocities, and repeated motion cycles, while signals from multiple anatomical sites can represent coordinated movements or body postures [249]. For reliable monitoring, the gel should accommodate the deformation range of the target joint and maintain a repeatable output with limited hysteresis and drift during cyclic motion. For example, Tang et al. integrated a resistive PVA/PAA/LiCl organogel sensor with wireless electronics and continuous passive motion equipment for knee rehabilitation. The sensor showed a sensitive and continuous relative resistance response over 0–1250% strain, covering motions from small vibrations to large limb deformations (Figure 15a,b). After slight initial drift, the sensor maintained repeatable responses over 2000 cycles at 60% strain (Figure 15c). When mounted on the knee, its resistance changed approximately linearly with flexion angle from 0° to 90°, and the calculated angles closely matched the measured values (Figure 15d,e) [42]. These results demonstrate the effectiveness of conductive gel-based strain sensors in converting repeated joint deformation into stable, angle-dependent electrical responses for quantitative and continuous joint-motion monitoring.

Gait monitoring instead examines repeated foot-ground contact and plantar loading [250]. Pressure sensors containing conductive gels or triboelectric devices in which the gels function as compliant electrodes can be positioned on the sole or incorporated into an insole to detect standing, walking, running, and jumping [251]. A single sensing site can provide temporal information such as step events, while distributed sensors can map plantar-pressure patterns; these outputs can also be used to differentiate gait modes or abnormal gait patterns [251]. Because foot-mounted devices experience repeated compression and impact, mechanical recovery, response speed, and cyclic stability are particularly relevant to reliable gait monitoring. For instance, Hu et al. developed a smart insole with three sensing locations using pressure sensors based on a PAAm/xanthan gum hydrogel reinforced with Li/Al-layered double hydroxide (LDH) (Figure 15f–j). The sensors exhibited a sensitivity of 0.074 kPa^−1^ over 0.2–20 kPa and stable responses over 3600 loading cycles. Their distributed signals identified foot contact, mapped regional loads, and distinguished normal and varus foot types [252]. These results show that conductive gels can serve as effective pressure-sensing elements in wearable gait-monitoring systems by converting repeated foot-ground contact and regional plantar loading into stable electrical outputs for real-time analysis.

Conductive gels can support musculoskeletal motion monitoring by serving as deformable sensing elements that convert body deformation and mechanical loading into electrical signals [42,249,252]. Since movement, loading, and attachment conditions vary across applications, sensor placement, calibration, and dynamic response need to be matched to the intended task. For example, in joint-motion monitoring, calibration against reference joint angles and reproducible placement relative to the joint are important, while stable attachment helps limit errors caused by sensor slippage or changes in strain transfer [42]. In gait monitoring, calibration over the relevant pressure range and consistent positioning at defined plantar sites are important for comparing regional loading, while stable integration within the insole and adequate response and recovery times help preserve signals during successive foot contacts [252]. More generally, repeated loading and changes in temperature or gel hydration can alter sensitivity and baseline output, affecting the validity of calibration during prolonged use [253,254]. Therefore, tracking hysteresis, baseline drift, and SNR alongside the accuracy of inferred movement or loading parameters can help assess the practical reliability of gel-based musculoskeletal motion monitoring under the intended loading and wearing conditions.

**Figure 16:**
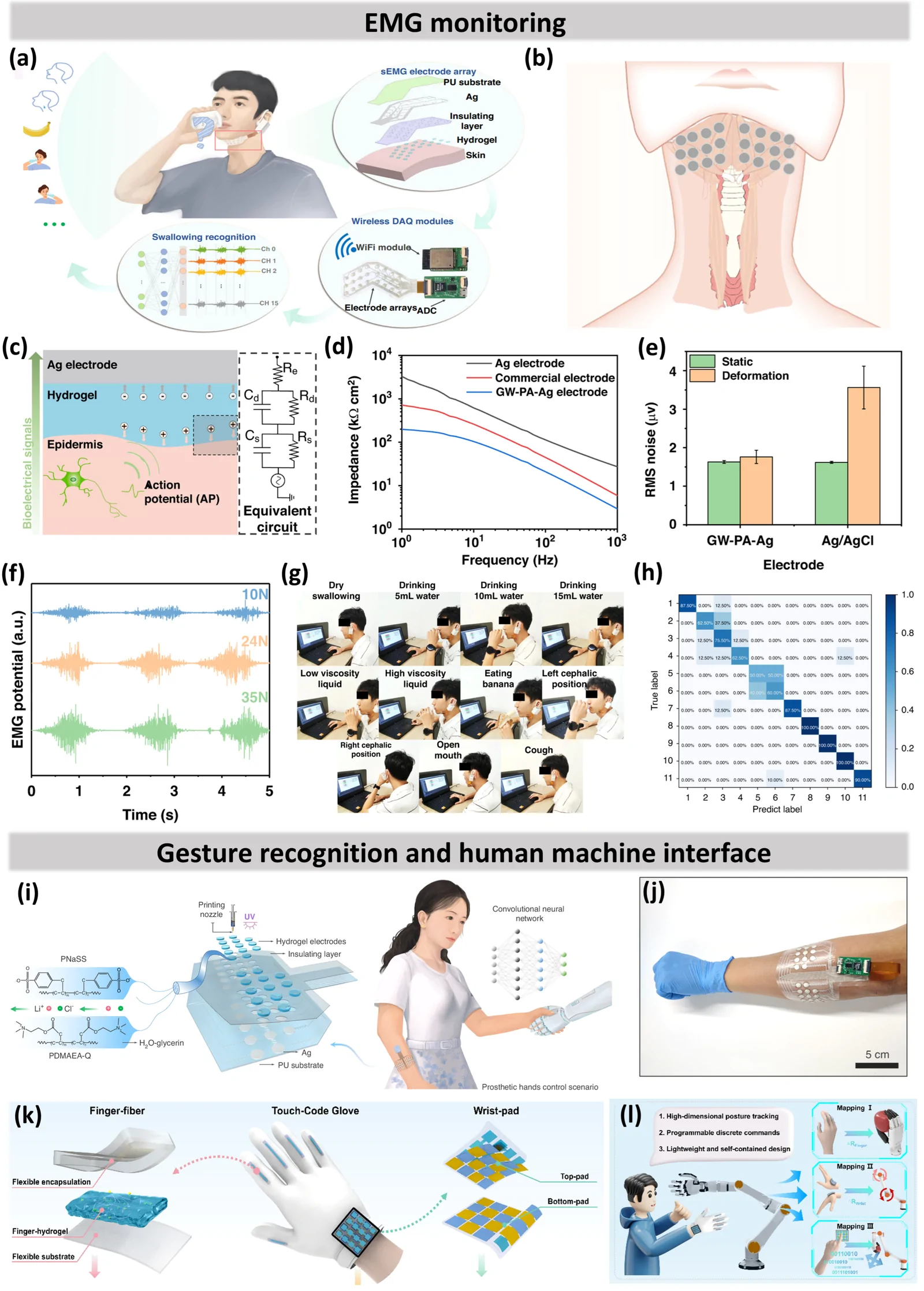
**Representative musculoskeletal applications of conductive gels for EMG monitoring, gesture recognition, and human-machine interfaces.** (**a**–**h**) Stretchable high-density surface electromyography (HD-sEMG) electrode array incorporating glycerol-water polyampholyte hydrogel-silver (GW-PA-Ag) contacts for swallowing monitoring and classification. (**a**) System integrating the electrode array, wireless data-acquisition modules, and swallowing-recognition model. (**b**) Placement of the V-shaped electrode array over the submandibular swallowing muscles. (**c**) Bioelectrical coupling at the skin-hydrogel–electrode interface and the corresponding equivalent circuit. (**d**) Electrode-skin impedance of GW-PA-Ag, commercial Ag/AgCl, and dry Ag electrodes over 1–1000 Hz. (**e**) Root-mean-square noise under static and skin-deformation conditions. (**f**) sEMG signals measured during submandibular exercises against loads of 10, 24, and 35 N. (**g**) Eleven swallowing-related activities used for classification. (**h**) Confusion matrix for classification of the eleven activities. Adapted from Zhang et al. [257]. © The Author(s) 2023. Published by Springer Nature under a CC BY 4.0 license. (**i**,**j**) Three-dimensional (3D)-printed polyion-complex glycerol (PIC-G) hydrogel HD-sEMG system for gesture recognition and prosthetic-hand control. (**i**) PIC-G hydrogel design, construction of the printed electrode array, convolutional neural network-based gesture recognition, and prosthetic-hand control concept. (**j**) Integrated HD-sEMG electrode array and readout circuit worn on the forearm. Adapted from Lai et al. [54]. © The Author(s) 2025. Published by Springer Nature under a CC BY 4.0 license. (**k**,**l**) Multimodal Touch-Code Glove integrating hydrogel strain sensors and a triboelectric touch interface for robotic control. (**k**) Device architecture comprising hydrogel-based Finger-fibers and a multimodal Wrist-pad. (**l**) Finger-to-hand, wrist-to-arm, and touch-to-path mapping strategies for controlling a dexterous hand and robotic arm. Adapted from Sun et al. [258]. © The Author(s) 2025. Published by OAE Publishing Inc. under a CC BY 4.0 license.

Unlike the mechanical measurements described above, EMG records the electrophysiological activity associated with muscle contraction and relaxation [248]. In wearable systems, conductive gels can form the skin-contacting layer of soft epidermal electrodes, conforming to deforming skin while coupling ionic signals from the body to electronic conductors [255]. Depending on the motor task and electrode arrangement, EMG amplitude and multichannel spatiotemporal patterns can distinguish muscle-activation levels and functional actions [256]. Reliable recording from curved or dynamically deforming skin requires low electrode-skin impedance, sufficient signal quality, conformal adhesion, and stability against dehydration during extended use. For example, Zhang et al. developed a stretchable high-density surface electromyography (HD-sEMG) patch for monitoring and classifying swallowing-related activity from the submandibular muscles (Figure 16a). A conductive glycerol-water polyampholyte (GW-PA) hydrogel was polymerized in situ on screen-printed Ag electrodes, forming ion-conducting GW-PA-Ag contacts that capacitively coupled ionic currents in the skin to electronic currents in the electrodes (Figure 16c). These contacts showed lower electrode-skin impedance than commercial Ag/AgCl gel and dry Ag electrodes over 1–1000 Hz (Figure 16d). During skin deformation, their root-mean-square (RMS) baseline noise remained approximately 1.7 μV, compared with 3.6 μV for commercial electrodes (Figure 16e). The V-shaped array contained 24 contacts, including 16 working and eight reference electrodes, and was placed beneath the jaw for wireless 16-channel recording (Figure 16b). During a swallowing-muscle exercise against spring resistances of 10, 24, and 35 N, peak surface EMG (sEMG) amplitude increased from 150 to 505 μV and SNR from 25 to 34 dB, distinguishing the three force levels (Figure 16f). A convolutional neural network classified eleven swallowing-related actions performed by five healthy volunteers with 80% average accuracy (Figure 16g,h) [257]. By maintaining signal quality on curved and dynamically deforming skin, conductive gels can provide a stable recording interface that enables multichannel EMG systems to quantify muscle activation intensity and classify functional actions.

Beyond signal acquisition and monitoring, musculoskeletal signals acquired through conductive gel interfaces can be decoded for gesture recognition and translated into commands for assistive or robotic systems. For an EMG-based example, Lai et al. fabricated a high-density sEMG electrode array incorporating 3D-printed polyion-complex glycerol (PIC-G) hydrogel contacts and positioned the 16-channel patch over target forearm muscles (Figure 16i,j) [54]. A convolutional neural network classified 26 hand gestures from the recorded sEMG signals with an average accuracy of 87.36%, and the decoded labels were used to command a prosthetic hand to reproduce the corresponding gestures.

Whereas conductive gel electrode-based EMG sensing can enable gesture-recognition systems by recording electrophysiological activity from muscle groups associated with the target movement, conductive gel-based strain sensing provides a complementary pathway by deriving control inputs directly from joint movements, including movements of the fingers, wrist, and other body joints. In a joint-motion-based implementation, as illustrated in Figure 16k,l, Sun et al. integrated ionically conductive sodium alginate/PAAm/NaCl double-network hydrogel strain sensors into the finger-fibers and wrist-pad of the Touch-Code Glove, together with a triboelectric tactile array. The hydrogel sensors linearly tracked finger bending and wrist flexion/extension over 0–90° through relative-resistance changes, enabling finger gestures to be mapped to corresponding motions of a dexterous robotic hand and wrist motion to the vertical movement of a robotic arm. Separately, the triboelectric array converted touch paths into programmable commands for real-time robotic-arm control [258].

Therefore, conductive gels can support diverse applications in wearable and attachable musculoskeletal monitoring, including deformable sensing of joint motion and plantar loading as well as skin-conformal bioelectrical coupling for EMG recording. When these mechanical and electrophysiological measurements remain accurate and stable during repeated loading and dynamic skin deformation, they can further serve as quantitative inputs for applications such as rehabilitation monitoring, gesture recognition, prosthetic control, and human–robot interaction [249,256].

### 5.3. Neurological and Ocular Bioelectrical Monitoring

Neurological and ocular monitoring extend the electrophysiological applications of conductive gels beyond cardiac and skeletal-muscle signals to bioelectrical activity acquired from the scalp and the skin surrounding the eyes [259,260]. EEG records electrical activity generated by the brain [261], whereas EOG measures changes in electrical potential around the eyes associated with gaze shifts, blinking, and eye closure [260]. In both cases, conductive gels can serve as compliant epidermal electrodes that provide bioelectrical coupling between the skin and electronic recording components while conforming to curved or dynamically deforming surfaces [262]. However, scalp hair and complex head contours complicate electrode contact for EEG, whereas repeated facial movement can challenge stable adhesion around the eyes [178]. Therefore, parameters such as skin-electrode impedance, SNR, waveform fidelity, resistance to motion artifacts, and stability during prolonged wear can serve as key criteria for evaluating these conductive gel-based devices [263].

**Figure 17:**
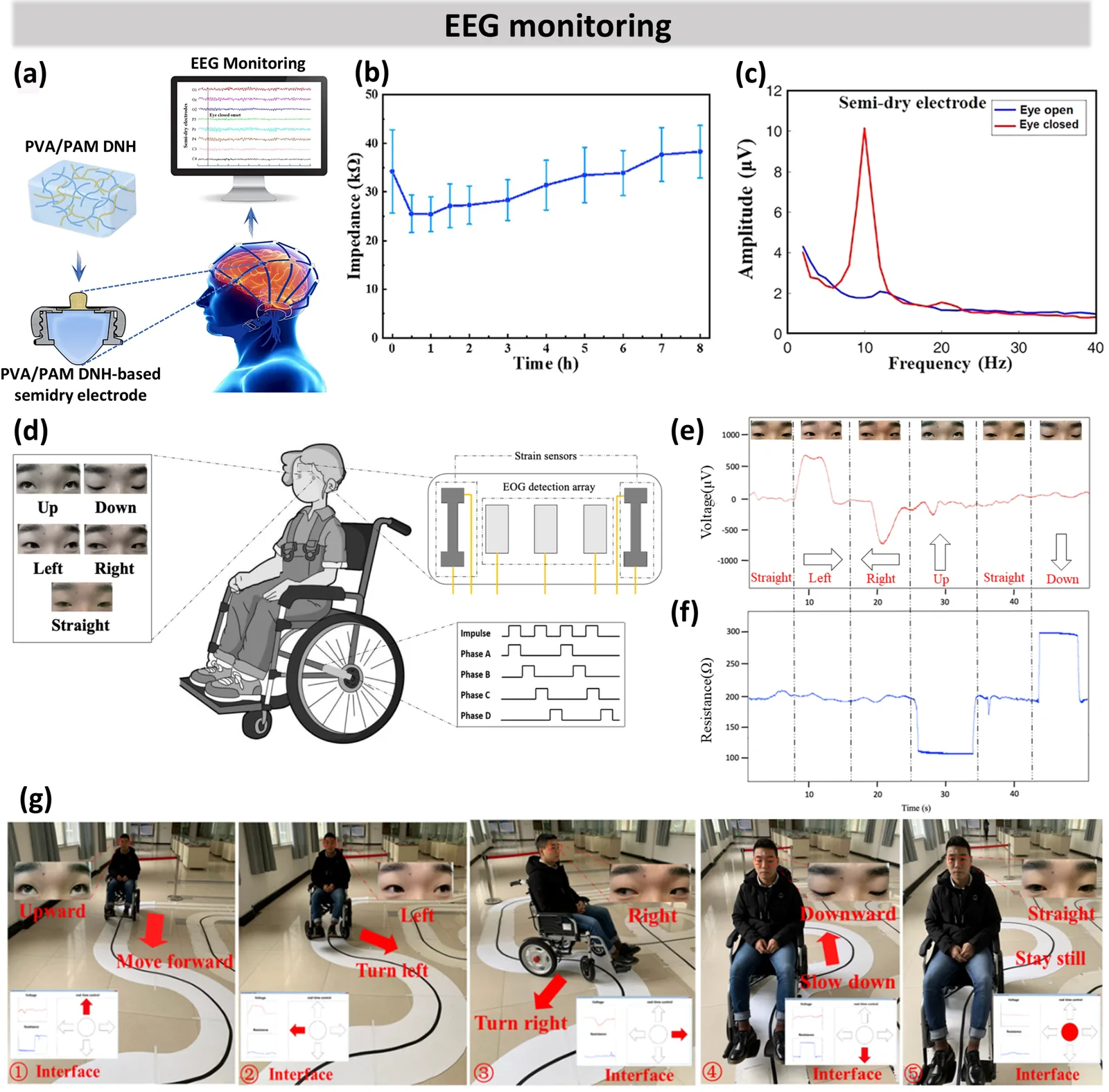
**Representative neurological and ocular bioelectrical applications of conductive gels for EEG monitoring and eye-movement-controlled assistive interfaces.** (**a**–**c**) Electroencephalography (EEG) monitoring using semidry electrodes incorporating a saline-loaded polyvinyl alcohol/polyacrylamide dual-network hydrogel (PVA/PAAm DNH). (**a**) PVA/PAAm DNH used as an electrolyte reservoir in a semidry scalp electrode for EEG acquisition. (**b**) Electrode-scalp impedance measured at 10 Hz over 8 h. (**c**) EEG frequency spectra under eyes-open and eyes-closed conditions, showing the alpha rhythm during eye closure. Adapted from Li et al. [264]. © 2023 The Authors. *SmartMat* published by Tianjin University and John Wiley & Sons Australia, Ltd. under a CC BY 4.0 license. (**d**–**g**) Eye-movement-controlled wheelchair based on a forehead-mounted biosensor comprising a conductive hydroxypropyl cellulose/polyvinyl alcohol (HPC/PVA) hydrogel and a flexible PDMS substrate. (**d**) Wheelchair-control architecture showing the electrooculogram (EOG) detection array, strain sensors, five eye-movement states, and pulse signals for driving the stepper motors. (**e**) EOG potential response during straight, leftward, rightward, upward, and downward eye movements. (**f**) Resistance changes associated with forehead deformation during the same eye-movement sequence. (**g**) On-site demonstration mapping upward, leftward, rightward, downward, and straight gaze states to forward motion, left and right turns, deceleration, and stopping, respectively. Adapted from Wang et al. [267]. © 2021 by the authors. Published by MDPI under a CC BY 4.0 license.

EEG provides information on brain activity that can be analyzed through features such as alpha rhythms, responses to open- and closed-eye conditions, and changes associated with wakefulness and sleep stages [263]. Conductive gel EEG electrodes can employ preformed gel contacts [261], semidry electrolyte reservoirs [178], or paintable formulations that undergo gelation in situ to improve contact with the forehead or hair-covered scalp [176]. Their practical performance should be established by determining whether characteristic EEG features remain distinguishable without extensive skin preparation and whether signal quality is retained during movement or prolonged recording. For example, Li et al. developed a semidry EEG electrode comprising a tapered, saline-swollen PVA/PAAm dual-network hydrogel (DNH) electrolyte reservoir beneath an Ag/AgCl-coated housing (Figure 17a). In ten participants, the electrodes were placed without skin pretreatment at eight hair-covered scalp sites (O1, O2, Oz, P3, P4, Pz, C3, and C4), with adjacent wet electrodes as references. The DNH supplied saline for ionic coupling while maintaining the stable nonpolarizable Ag/AgCl contact, yielding an offset potential comparable to the hydrogel-free configuration and lower potential drift. In a representative participant, the 10-Hz impedance of a semidry-electrode pair decreased from 34.3 ± 8.6 kΩ initially to 25.4 ± 3.6 kΩ after 1 h as saline accumulated, then gradually increased to 38.2 ± 5.6 kΩ after 8 h (Figure 17b). The electrodes resolved the 8–12 Hz alpha rhythm during eye closure and its suppression after eye opening (Figure 17c). Their signals also showed a mean temporal cross-correlation coefficient of 0.919 ± 0.054 with wet-electrode recordings. Thus, the integrated electrolyte reservoir supported EEG recording comparable to wet electrodes with stable scalp-contact impedance over 8 h [264]. These results can demonstrate whether the gel electrode addresses the specific interfacial challenges of scalp-based monitoring rather than merely recording EEG under a short, stationary condition.

Maintaining the quality of prolonged neurological recordings with conductive gels depends on gel hydration and stable skin contact [157]. For EEG, wet, dry, and semidry electrode configurations differ in electrolyte use and scalp contact. Conventional wet electrodes use applied conductive gel or paste to achieve low impedance, but require preparation and cleaning, and gel drying can destabilize contact. Dry electrodes simplify preparation by avoiding added electrolyte, but can exhibit high or unstable impedance and increased susceptibility to motion artifacts [157,264]. Semidry electrodes can combine low impedance with reduced gel handling by supplying small amounts of electrolyte, and some designs use hydrogels as compliant reservoirs [264]. For example, Wang et al. reported an SNR of 20.02 dB for semidry hydrogel electrodes during N170 recording, comparable to that of the tested wet electrodes [178]. Nevertheless, semidry designs may require electrolyte replenishment, while dehydration or inadequate adhesion in hydrogel designs can compromise prolonged contact [157,264]. Practical comparisons of conductive gel electrodes therefore need to consider electrode preparation time, placement reproducibility, adhesion or fixation stability, and changes in skin-electrode impedance and SNR during movement and over prolonged recording periods. Such evaluations can help determine whether effective bioelectrical coupling is maintained under the intended conditions of use [264].

EOG instead detects eye position and movement using electrodes placed around the eyes. Differences in the recorded voltage patterns can distinguish horizontal or vertical gaze shifts, blinking, and voluntary eye closure, supporting applications such as fatigue or alertness assessment, eye-movement monitoring, and assistive communication [265,266]. Reliable EOG acquisition requires conformal contact with thin and frequently moving forehead or periocular skin, together with repeatable outputs during successive eye movements. Because the voltage-gaze relationship depends on electrode position, quantitative EOG-based gaze estimation benefits from reproducible placement and calibration using defined eye positions [265]. Hybrid configurations may additionally combine EOG with strain sensing of forehead deformation to support multidirectional gaze recognition. For example, Wang et al. developed an eye-controlled wheelchair prototype using a forehead sensor with a central EOG array and two lateral hydroxypropyl cellulose/polyvinyl alcohol (HPC/PVA) conductive hydrogel strain sensors in PDMS (Figure 17d). The EOG array showed 39.6 kΩ skin-contact resistance at 100 Hz versus 43.2 kΩ for Ag/AgCl electrodes and opposite-polarity responses for leftward and rightward gaze. The strain sensors showed 7.8 mV∙N^−1^ force sensitivity and a 103 ms response time, distinguishing upward and downward gaze through opposite resistance changes from forehead compression and stretching (Figure 17e,f). A wavelet transform-support vector machine (WT-SVM) combined both signals to classify the five gaze states with 96.3% average accuracy, compared with 91.9% for the Ag/AgCl-based system. Mapping them to stopping, forward motion, deceleration, left-turning, and right-turning commands enabled nine of ten volunteers to navigate a test bend (Figure 17g) [267]. These results suggest that conductive gels can support stable EOG acquisition by maintaining suitable bioelectrical coupling with the deforming skin of the forehead or periocular region, while hybrid configurations that integrate EOG with complementary biosignals can enable more comprehensive healthcare systems.

Beyond recording physiological states, EEG and EOG signals can be processed into commands for brain–computer interfaces, gaze-based communication, or assistive-device control [268]. Evaluation at this stage should extend beyond electrode impedance and waveform quality to include system-level outcomes such as classification accuracy, the number of distinguishable commands, response latency, and successful completion of a communication or control task [268]. Conductive gels therefore contribute to neurological and ocular healthcare not only by maintaining stable bioelectrical coupling on challenging anatomical surfaces, but also by providing sufficiently reliable signals for monitoring, interpretation, and assistive interaction.

### 5.4. Biochemical and Cutaneous Health Monitoring

Whereas the preceding applications largely track electrophysiological or mechanical activity, biochemical and cutaneous monitoring addresses molecular and local physicochemical information available at the skin or wound surface [269]. Sweat contains metabolites and electrolytes, while skin temperature and humidity provide information on the local thermal and moisture state; wound exudate can additionally carry chemical or biological indicators associated with healing or infection [270,271]. Depending on the device architecture, conductive gels can function as analyte-permeable matrices, supports for immobilized recognition elements, ion- or electron-transporting layers, or temperature- and moisture-responsive sensing elements [269]. Their softness enables conformal attachment, but variations in sweat rate, temperature, humidity, gel swelling or dehydration, biofouling, and body movement can introduce signal drift and cross-sensitivity during extended monitoring [270].

**Figure 18:**
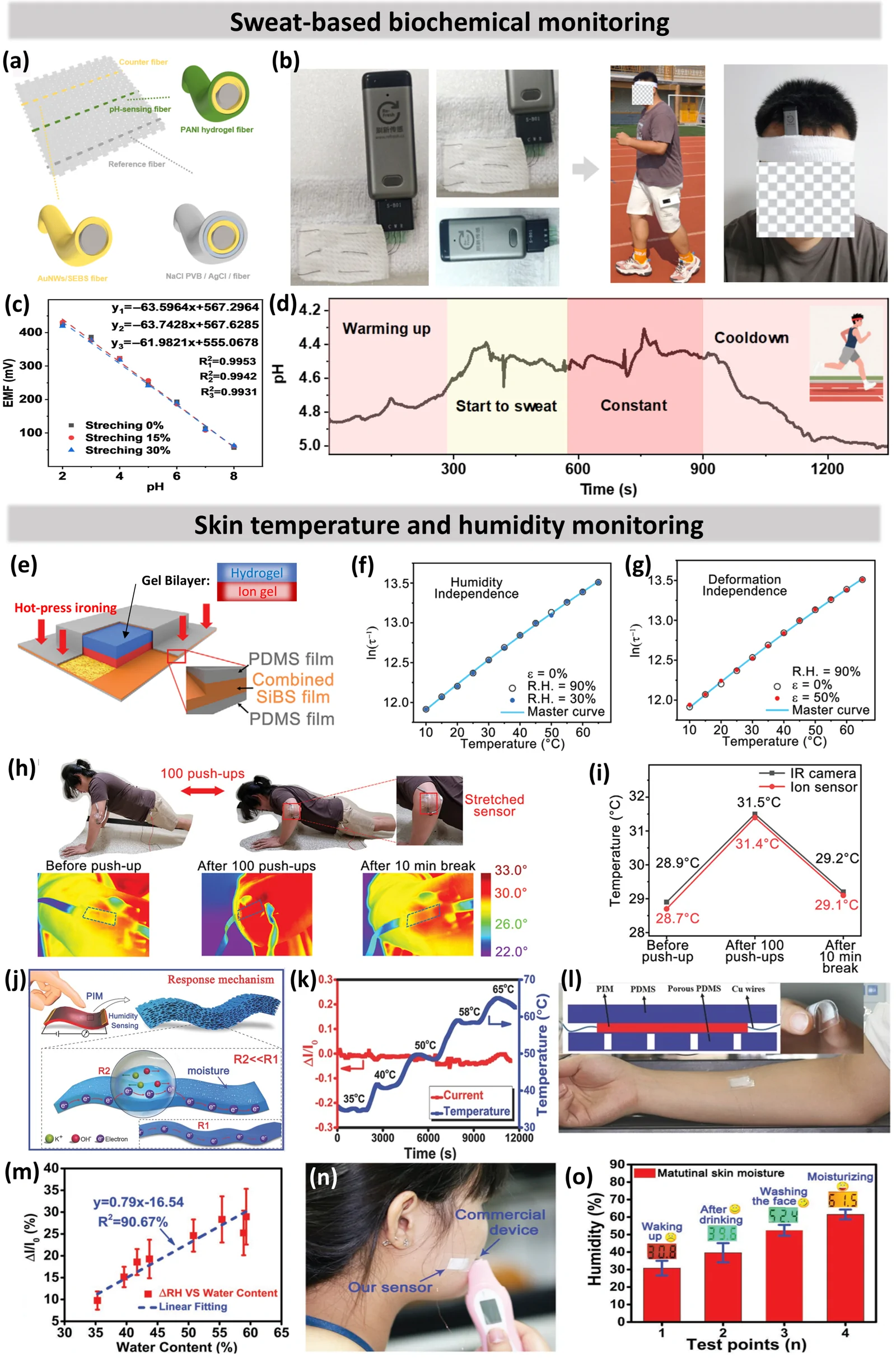
**Representative biochemical applications of conductive gels for sweat-pH, skin-temperature, and skin-humidity monitoring.** (**a**–**d**) Potentiometric sweat-pH monitoring using a polyaniline-phytic acid-poly(vinyl alcohol) (PANI-PA-PVA) hydrogel fiber sensor. (**a**) Textile-integrated sensing configuration comprising a PANI hydrogel pH-sensing fiber, an AuNW/SEBS counter fiber, and a NaCl/PVB/AgCl reference fiber. (**b**) Configuration of a wearable sweat pH-monitoring device, including a three-electrode hydrogel sensor and custom-developed mobile applications, with photographs showing its use during exercise. (**c**) Electromotive-force (EMF) calibration as a function of pH after stretching. (**d**) Real-time monitoring of sweat pH during warm-up, the onset of sweating, constant exercise, and cooldown. Adapted from Li et al. [130]. © 2025 by the authors. Licensee MDPI, Basel, Switzerland. Published under a CC BY 4.0 license. (**e**–**i**) Humidity- and deformation-independent epidermal temperature sensing using a water-saturated hydrogel/ionogel bilayer. (**e**) Hot-press-sealed device architecture integrating the gel bilayer with flexible electrodes and PDMS/SiBS encapsulation films. (**f**) Relationship between the inverse charge-relaxation time, ln(τ^−1^), and temperature under different relative humidities, demonstrating humidity-independent temperature sensing. (**g**) Temperature calibration in the unstretched and 50–stretched states, demonstrating deformation-independent sensing. (**h**) On-body temperature measurement with an elbow-mounted sensor during 100 push-ups, together with infrared thermal images obtained before exercise, immediately after exercise, and after a 10-min break. (**i**) Comparison of temperatures measured using the ionogel sensor and an infrared camera at the three exercise stages. Adapted from Kim et al. [89]. © 2022 The Authors. Advanced Science published by Wiley-VCH GmbH under a CC BY 4.0 license. (**j**–**o**) Flexible porous ionic membrane (PIM)-based humidity sensing for skin-hydration assessment. (**j**) Humidity-sensing mechanism in which moisture adsorption within the porous membrane produces low-resistance ionic conduction pathways. (**k**) Current response during stepwise temperature changes at constant humidity, demonstrating low temperature cross-sensitivity. (**l**) Skin-mounted smart-label configuration comprising the PIM sandwiched between PDMS and porous PDMS films. (**m**) Linear relationship between the relative current response and skin water content measured using a commercial reference device. (**n**) Facial-skin hydration measurement using the PIM sensor and a commercial device. (**o**) Changes in facial water content after waking, drinking water, washing the face, and applying moisturizer. Adapted from Li et al. [282]. © 2017 The Authors. Published by WILEY-VCH Verlag GmbH & Co. KGaA, Weinheim, under a CC BY 4.0 license.

Sweat-based biochemical monitoring can target metabolites such as glucose and lactate, acidity expressed as pH, and electrolytes such as Na^+^ and K^+^ [272,273]. In electrochemical configurations, enzymes, ion-selective recognition elements, or intrinsically analyte-responsive conductive polymers can be incorporated within or adjacent to a conductive gel, while the hydrated and porous network facilitates analyte transport to the sensing interface [274,275]. The resulting biochemical reaction, ion activity, or protonation state can be converted into current, potential, impedance, or another measurable electrical output. For example, Li et al. developed a real-time textile potentiometric sweat pH sensor by coating porous polyaniline-phytic acid-poly(vinyl alcohol) (PANI-PA-PVA) conductive hydrogel on an elastic Au-fiber working electrode paired with Ag/AgCl reference and Au counter fibers (Figure 18a,b). PANI protonation–deprotonation and redox changes produced an H^+^-dependent open-circuit potential against the Ag/AgCl reference, while the hydrated porous network supported proton transport. The sensor responded linearly over pH 2.0–8.0 with 65.4 mV∙pH^−1^ sensitivity (R^2^ = 0.9954) and remained stable under stretching and bending (Figure 18c). For on-body testing, the textile was worn on one volunteer’s forehead during a 5-min warm-up, 10 min vigorous running, and 5 min rest, with wireless smartphone readout. After sweating began, the potential decreased and stabilized upon sweat saturation, corresponding to pH 4.2–5.0, and measurements of three sweat samples agreed closely with a commercial pH meter (Figure 18d) [130].

Because conductive gels mediate analyte transport and electrochemical coupling, quantitative biochemical measurements depend on both the properties of the sampled fluid and the stability of the sensing interface [274,275]. Calibration against known analyte concentrations or activities therefore needs to reflect the intended sampling conditions and fluid composition, while accounting for temperature effects and the stability of the sensing and reference electrodes [276]. Comparisons with independent analyses of collected samples can help assess whether the calibration remains valid during wearable biochemical monitoring [277].

Skin temperature and skin-surface humidity provide complementary physical indicators of the cutaneous state and its local microenvironment [278,279]. Temperature-responsive conductive gels can convert local thermal changes into variations in resistance, ionic conductivity, voltage, or an impedance-derived parameter such as charge-relaxation frequency [278], whereas skin-surface humidity can be evaluated from moisture-induced changes in current, conductance, impedance, or capacitance [280]. When calibrated against a reference skin-moisture measurement, this humidity response may also provide an indirect estimate of skin water content [279]. Because temperature, humidity, and mechanical deformation can simultaneously affect ion transport and sensor geometry, practical devices should be assessed for response and recovery times, hysteresis, repeatability, temperature–humidity cross-sensitivity, strain-induced interference, and stability under changing ambient conditions [280,281].

For temperature-focused monitoring, Kim et al. developed a stretchable epidermal sensor in which a water-retaining hydrogel kept a hydrophobic ionogel sensing layer water-saturated, while a low-water-permeability bilayer of PDMS and polystyrene-block-poly(isobutylene)-block-polystyrene (SiBS) limited water exchange (Figure 18e). Temperature was calculated from the charge-relaxation frequency (τ^−1^) derived from bulk resistance and capacitance measured by EIS, rather than from either value alone. Maintaining the ionogel water content and using this geometry-insensitive intrinsic parameter minimized humidity and deformation interference. The average temperature error was 0.12 °C between 30% and 90% RH and 0.13 °C under 50% strain (Figure 18f,g). When worn on the elbow during 100 push-ups, its readings differed by less than 0.2 °C from an infrared camera before and after exercise (Figure 18h,i) [89]. In a complementary humidity-focused example, Li et al. fabricated a PVA/potassium hydroxide (KOH) porous ionic membrane (PIM) from a polymer gel electrolyte and measured humidity-induced current changes (Figure 18j). At 45% RH, heating from 35 to 65 °C caused only a small current change, indicating low temperature cross-sensitivity (Figure 18k). For on-skin sensing, the PIM was placed between a porous inner PDMS layer that delivered skin moisture without direct contact and a nonporous outer PDMS layer that limited ambient humidity interference (Figure 18l). Its sensitivity correlated linearly with skin water content measured by a commercial instrument (R^2^ = 0.9067), enabling current-based estimates of skin water content (Figure 18m). The label tracked facial skin-moisture changes associated with drinking water, face washing, and moisturizer application (Figure 18n,o) [282]. Together, these studies illustrate reciprocal interference-control strategies: maintaining a water-saturated ionogel for humidity-resistant temperature sensing and using a PIM with low temperature cross-sensitivity to estimate skin moisture from skin-surface humidity.

**Figure 19:**
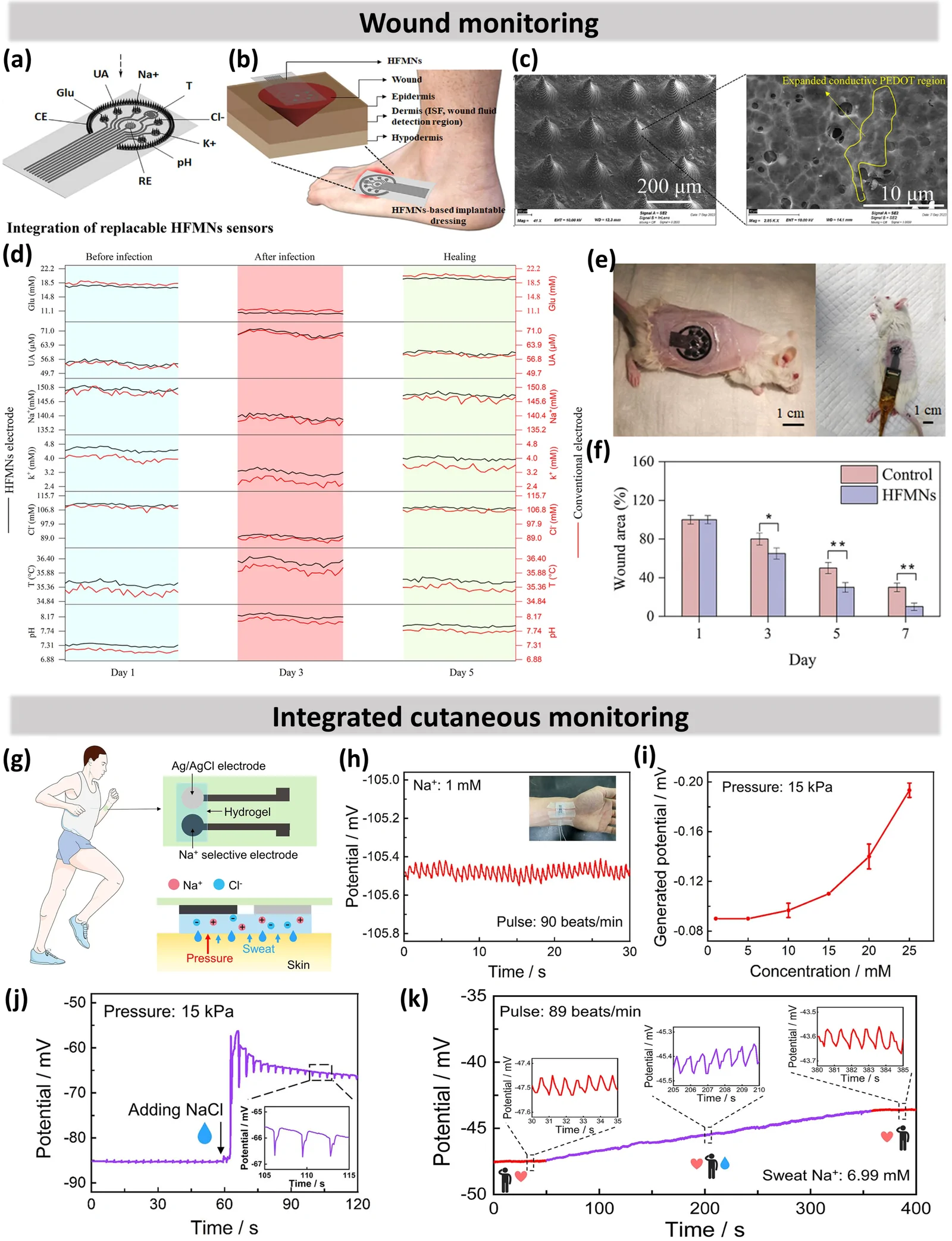
**Representative cutaneous health monitoring platforms of conductive gels for wound monitoring and integrated cutaneous monitoring.** (**a**–**f**) Hydrogel-forming microneedle (HFMN) array-based transdermal dressing for multiplexed wound assessment and therapy. (**a**) Polar array of replaceable HFMN sensors for glucose (Glu), uric acid (UA), Na^+^, K^+^, Cl^−^, pH, and temperature, together with counter and reference electrodes. (**b**) Dressing configuration in which the HFMNs penetrate the wound surface to access dermal wound interstitial fluid for continuous biomarker monitoring. (**c**) Scanning electron microscopy images of the conical HFMN array and the expanded porous PEDOT-rich conductive network produced by laser-scribed phase separation. (**d**) Comparative monitoring of seven wound biomarkers using HFMN electrodes sampling dermal wound interstitial fluid and conventional electrodes sampling surface exudate before infection, after infection, and during healing. (**e**) Application of the HFMN dressing and associated electrochemical measurement setup in a rat wound model. (**f**) Comparison of the remaining wound area in control and HFMN-treated groups over seven days. Data are presented as mean ± SD (n = 4 rats per group). * *p* < 0.05, ** *p* < 0.01, and *** *p* < 0.001. Statistical comparisons between groups were performed using two-way ANOVA followed by Tukey’s post hoc test. Adapted from Sharifuzzaman et al. [285]. © 2025 The Author(s). Small published by Wiley-VCH GmbH under a CC BY 4.0 license. (**g**–**k**) Dual-mode wearable sensor integrating sweat Na^+^ detection and pulse-pressure sensing within a single hydrogel-coupled ion-selective electrode. (**g**) Device structure and sensing concept, in which the hydrogel collects sweat for Na^+^ analysis while generating a piezoionic potential in response to pressure. (**h**) On-body wrist-pulse signal recorded using hydrogel containing 1 mM Na^+^. (**i**) Dependence of the pressure-generated potential on Na^+^ concentration under a constant applied pressure. (**j**) Simultaneous response to periodic pressure and an increase in NaCl concentration, distinguished by periodic potential fluctuations and a shift in the potential baseline, respectively. (**k**) On-body measurement demonstrating simultaneous extraction of pulse rate and sweat Na^+^ concentration from a single potential signal. Adapted from Ma et al. [286]. © The Author(s) 2024. Published by OAE Publishing Inc. under a CC BY 4.0 license.

Wound monitoring presents a distinct cutaneous application because the sensing interface must operate on an irregular, moist, and exudate-rich wound bed or, in transdermal configurations, access wound-related fluid beneath the surface [271]. Conductive gel dressings can provide conformal ionic or electronic coupling and may also serve as hydrated sampling matrices while incorporating sensing elements for wound pH, glucose, temperature, moisture, strain, or infection-related microorganisms and biomarkers [283,284]. In addition to analytical sensitivity, these devices require biocompatibility, stability in the hydrated wound environment, and an interface that provides either secure, non-damaging surface contact or controlled, minimally invasive access to subsurface fluid without substantially disturbing healing tissue. For example, Sharifuzzaman et al. developed a theranostic transdermal dressing based on PVA/chitosan/MXene hydrogel-forming microneedle (HFMN) arrays (Figure 19a–f). Laser-scribed phase separation (LSPS) converted a PEDOT/graphene oxide coating into an electrode platform that remained stable in water for glucose, uric acid, Na^+^, K^+^, Cl^−^, pH, and temperature sensing. The sensors showed target selectivity and stable calibration for at least five days in simulated wound fluid. As part of the in vitro biocompatibility assessment, the LSPS-treated HFMNs containing 5 wt% MXene exhibited a mean fibroblast viability of 81.04% after seven days in a WST-8 assay, with no statistically significant difference from the cell control. The system also showed in vitro antibacterial activity and, in infected wounds in rats, monitored temporal changes in pH, temperature, and glucose while promoting wound closure [285]. This study illustrates how a conductive gel microneedle interface can combine minimally invasive sampling, multiplexed sensing, and therapeutic function.

For practical use in wound monitoring, conductive hydrogel microneedles need sufficient strength for insertion and stable sensing after fluid uptake [287,288]. In the example above, prolonged swelling reduced sensitivity, while higher MXene loading reduced fibroblast viability [285]. Clinical use also requires evidence that microneedle-based monitoring is tolerable and does not impair wound healing. Therefore, human wound studies are needed to quantify pain and tissue damage during insertion, wear, and removal, monitor infection, and determine whether repeated application impairs healing [289,290,291]. Repeated application of new patches differs from reuse of the same array. If reuse is intended, reprocessing procedures need to preserve microbial safety, needle integrity, and sensing performance [290,292].

A further direction is integrated cutaneous monitoring, in which biochemical information from sweat or wound exudate is acquired together with temperature, skin-surface humidity, strain, pressure, or other physiological variables [272,293]. Such multimodal measurements can provide complementary biochemical and physical information within a single wearable platform. Signal specificity may be maintained using strategies such as separately addressed sensing elements [294], differentiated transduction mechanisms [295], calibration [274], data-based decoupling [281], or distinct features of a shared electrical output [296]. When multiple variables are encoded in the same output, simultaneous operation should demonstrate that each remains identifiable from features such as the signal baseline or temporal fluctuations, even when one stimulus influences the response magnitude associated with another. For example, Ma et al. developed a dual-mode wrist sensor by coupling a solid-state Na^+^-selective electrode to an ion-containing p(AM-co-PEGDA) hydrogel formed from acrylamide (AM) and poly(ethylene glycol) diacrylate (PEGDA) (Figure 19g–k). The porous hydrogel collected sweat for potentiometric Na^+^ sensing and generated a piezoionic potential under wrist-pulse pressure. Both were encoded in one open-circuit potential signal, with Na^+^ shifting the baseline and pulses producing periodic fluctuations. Bench testing showed that the baseline and periodicity could independently resolve Na^+^ concentration and loading frequency. Measurements on the wrist yielded 6.99 mM Na^+^ and 89 beats/min, consistent with separate measurements, and simultaneous monitoring was reproduced in two additional volunteers [286]. This example demonstrates how a single ionically conductive hydrogel interface can provide complementary sweat-electrolyte and cardiovascular information without requiring separate biochemical and pressure-sensing units. More broadly, such integrated designs highlight the potential of conductive gels as multifunctional interfaces for combined biochemical and physical monitoring, provided that the individual signals remain reliably distinguishable during simultaneous operation.

Together with the cardiovascular, respiratory, musculoskeletal, neurological, and ocular applications described above, these biochemical, cutaneous, and integrated examples demonstrate that conductive gels can function as compliant bioelectrodes, deformable sensing elements, matrices for analyte collection and transduction, and components of multimodal wearable or attachable systems. Therefore, the long-term reliability of conductive gels in these healthcare applications can be assessed by examining how changes in gel properties and interfacial contact during prolonged use affect signal quality and calibration stability under representative mechanical and environmental conditions. Across these roles, their practical value in healthcare ultimately depends on maintaining signal specificity, calibration validity, stable interfacial contact, and system-level reliability during prolonged attachment and realistic body movement [294,297]. Such long-term reliability also needs to be established under clinical conditions for devices intended to support diagnosis or treatment. Bench tests, animal studies, and preliminary volunteer demonstrations provide evidence of feasibility under the tested conditions, but do not by themselves establish sustained performance in patients [298]. To support real-world deployment, clinical evaluation in the intended patient population using suitable reference methods needs to be complemented by usability testing to identify training requirements and barriers to integration into routine care [299].

In addition to these performance considerations, short-term cytocompatibility assays and preliminary skin-contact or in vivo studies reported for several representative systems provide useful initial evidence concerning their biological compatibility [54,235,252,257,285,286]. However, these results do not necessarily predict long-term in vivo biocompatibility, because biological responses can be influenced by various factors associated with the conditions of use, such as the intended contact site and duration, repeated mechanical loading, and the release of residual components or degradation products during use [176,177]. Accordingly, evaluations for specific applications of the complete device over periods representative of its intended use, including long-term in vivo studies where appropriate, will be important for supporting the reliable and practical use of conductive gels in healthcare applications.

## 6. Conclusions and Future Perspectives

This review has examined how the composition and structure, sensing mechanisms, and biological and device interfaces of conductive gels collectively shape their functions and practical performance in wearable and attachable healthcare devices. The material platforms considered here, including hydrogels, ionogels, eutectogels, organogels, and organohydrogels, offer different combinations of properties such as electrical conductivity, deformability, solvent retention, environmental tolerance, and biocompatibility. Through mechanisms such as resistance modulation, capacitive or ionotronic coupling, temperature- or moisture-responsive electrical changes, and electrochemical transduction, these materials can serve in roles such as compliant bioelectrodes, deformable sensing elements, matrices for analyte transport and transduction, and multifunctional interfaces for multimodal monitoring or human–machine interaction.

No single gel platform, design strategy, or performance metric is universally optimal across these functions. Improving an individual property, such as conductivity, strength, adhesion, environmental resistance, or sensitivity, may alter other characteristics needed for conformal and prolonged operation. Moreover, favorable properties measured in isolated gel specimens do not necessarily ensure reliable sensing because practical performance also depends on factors such as coupling to the skin or other biological interfaces, gel–electrode and gel–device integration, encapsulation, fabrication reproducibility, signal acquisition, and calibration. Future progress is therefore likely to depend on application-specific co-design across the material, interface, device, and system levels, with particular attention to the long-term stability of the complete sensing pathway under the intended conditions of use.

### 6.1. Mechanical and Electrical Durability

Repeated deformation during body movement can progressively alter both the polymer network and the conductive pathways within a gel. Cyclic loading can produce fatigue damage and accumulated residual deformation, whereas mechanical hysteresis reflects energy dissipation and rate-dependent or irreversible network reorganization during loading and unloading. These effects can change gel geometry, internal stress distribution, and recovery behavior even in the absence of visible fracture. At the same time, deformation can alter ionic transport through changes in solvent and ion distribution, ion mobility, and gel dimensions or disrupt electronic transport through rearrangement, loss of contact, or fracture within filler networks or conductive polymer domains. Consequently, a gel may remain macroscopically intact while exhibiting changes such as drift in resistance or impedance, altered sensitivity, slower recovery, or reduced signal reproducibility [30,225].

Therefore, future material design may benefit from defining durability in relation to the mechanical demands of the intended sensing role rather than maximizing elongation at break alone. In terms of the conductive gel-based electronics, skin- or tissue-contacting devices require sufficient compliance and elastic recovery to maintain conformal contact during repeated motion; strain sensors require a working range matched to the target movement, together with low hysteresis and reproducible loading–unloading responses; and pressure sensors require rapid recovery and stable outputs under repeated compression. These requirements can be addressed through gel network designs that combine a structure-preserving framework with reversible, energy-dissipating interactions, as found in double or interpenetrating networks, combinations of permanent and dynamic crosslinks, and fiber- or particle-reinforced matrices [52,300]. However, these features need to be co-optimized because increasing crosslink density or reinforcement can improve strength and dimensional stability while reducing compliance, stretchability, or ion mobility, whereas slowly reforming energy-dissipating interactions can increase hysteresis and delay recovery [301]. Mechanical durability is therefore better assessed by the preservation of geometry, recovery behavior, and sensing performance under application-representative cyclic loading than by any single maximum value of stretchability, strength, or toughness.

In parallel, electrical durability can be considered in relation to the dominant charge-transport pathway. In ionically conductive gels, repeated deformation can alter solvent and ion distributions, gel dimensions, and ion mobility. Therefore, durability depends on maintaining a reproducible ionic state through coordinated control of solvent content, ion concentration, and network structure [302]. In electronically conductive gels, maintaining network connectivity is important. Therefore, homogeneous and continuously connected conductive polymer networks, uniform filler dispersion, strong interactions between conductive components and the polymer matrix, and mechanically resilient matrices that promote contact recovery after unloading can help limit irreversible disruption of conductive pathways [40,303]. Reversible coordination and hydrogen-bonding interactions may further reconstruct damaged polymer networks and restore electrical outputs, but self-healing is functionally meaningful only when both electrical conduction and the corresponding sensing response recover within a timescale relevant to the intended use [180]. Electrical durability is therefore better established by demonstrating stable or reproducibly recoverable sensing responses during application-representative cyclic deformation than by relying solely on initial conductivity or visible closure of damage.

### 6.2. Environmental Stability and Biosafety

The liquid phase contributes to gel softness and, in many systems, to charge transport, but it is also a major source of environmental instability. Aqueous gels can dehydrate and shrink in dry or warm environments and freeze at subzero temperatures, whereas humid or aqueous exposure, including perspiration, saline solution, or water, can cause swelling, changes in ion concentration, or the loss of mobile components. Gels based on less volatile liquid phases, including ionogels, eutectogels, and organogels, may reduce instability associated with water loss but can introduce other considerations related to ion mobility, solvent–polymer compatibility, liquid leakage, and biological safety [304,305]. These environmental changes can propagate into the sensing output by altering gel geometry, conductivity, and interfacial contact, thereby affecting aspects of performance such as baseline stability, sensitivity, and the validity of the original calibration.

Future formulation design should seek to regulate solvent activity, ion mobility, and network-liquid interactions according to the intended operating environment. In aqueous gels, strategies such as hygroscopic salts, humectants, strong polymer–water interactions, and partial solvent replacement can suppress evaporation or freezing. Mixed or alternative liquid phases used in systems such as organohydrogels, ionogels, and eutectogels may extend stability across a broader range of environments, while hydrophobic network design or multiple intermolecular interactions can limit excessive water uptake and swelling [306]. Selection among these approaches should be guided by their broader effects on properties such as conductivity, mechanical compliance, analyte transport, leakage, and skin compatibility. The objective is not simply to maximize solvent retention, but to maintain an appropriate and stable liquid composition and content throughout use.

Environmental management at the skin-facing side should balance gel solvent retention against requirements such as skin comfort, moisture exchange, and access to target analytes. Complete sealing can limit uncontrolled solvent and ion exchange but may restrict flexibility, breathability, or biochemical sampling. Strategies such as selective encapsulation, ultrathin or breathable covers, and directional moisture-transport pathways may help retain the internal liquid phase while preserving controlled exchange of water vapor, sweat, or target analytes at the skin interface [156]. In parallel, reversible or moisture-tolerant skin adhesion may be promoted through approaches such as bioinspired microstructures, dynamic interfacial bonds, or polar adhesive groups, helping maintain conformal contact under perspiration and body motion while allowing removal with minimal skin damage [307].

Long-term biosafety at the skin interface is also likely to depend on formulations with reduced residual or leachable components and biocompatibility appropriate to the intended duration and mode of contact. Materials such as natural polymers and bio-derived components, together with liquid phases selected for low volatility or a more favorable toxicological profile, may provide useful options [149]. However, none of these choices should be assumed to guarantee safety without evidence obtained from the complete formulation and finished device. Following U.S. Food and Drug Administration (FDA) guidance, relevant endpoints for wearable and attachable devices contacting intact skin include cytotoxicity, irritation, and sensitization. Wound-contacting devices, such as wound-healing dressings, additionally warrant consideration of systemic toxicity and material-mediated pyrogenicity, with the evaluation tailored to contact duration and exposure [308]. Even when contact is limited to intact skin, hydrogels and skin adhesives are excluded from FDA’s simplified intact-skin assessment approach because polymerization or degradation products can change over time in hydrogels, while skin adhesives can cause irritation [71]. These concerns warrant assessment of formulation-specific residues, leachables, and skin responses. Collectively, future environmental and skin-interface development should aim to preserve application-relevant outcomes such as material stability, skin compatibility, and signal quality under wearing conditions representative of the intended application.

For implantable devices, including systems attached to internal tissues, long-term evaluation can address local tissue responses such as inflammation and fibrous encapsulation, together with potential systemic toxicity from leachables or degradation products. Hemocompatibility is relevant when blood contact occurs. Chemical characterization and, where needed, implantation studies over relevant durations may help assess these risks while accounting for sterilization-related changes and material degradation [71,308].

### 6.3. Device-Level Reliability

At the device level, conductive gels must be integrated with components that are often more rigid or less deformable, including electrodes, substrates, interconnects, encapsulation layers, and readout modules. Mismatches in modulus, strain capacity, and hydration-induced dimensional changes can concentrate stress at their junctions, while differences in surface chemistry can weaken interfacial bonding or electrical coupling. These effects may lead to failure modes such as interfacial sliding, delamination, cracking, or loss of electrically active contact. Therefore, stable bulk properties of the gel are insufficient if mechanical loads and electrical signals cannot be transmitted reliably across the assembled device [309].

Future device interfaces may benefit from combining deformable conductors with bonding strategies selected for the gel chemistry and intended deformation mode. Deformable conductors such as liquid metals, conductive polymer composites, percolated nanowire or graphene networks, and structurally patterned electrodes can accommodate strain through mechanisms including fluid redistribution, network reconfiguration, and structural unfolding. In situ polymerization or gelation on porous or roughened electrodes can create mechanical interlocking, while surface functionalization can strengthen interfacial interactions or chemical bonding. Compliant interlayers, localized bonding, or mechanical anchoring may further redistribute deformation away from vulnerable junctions [310,311].

Encapsulation can similarly be designed as a functional interface rather than treated only as an external barrier. Future encapsulation strategies may combine low solvent permeability with sufficient compliance, stable adhesion to the gel, and selective exposure of regions needed for sensing. Thin or patterned encapsulation and strongly bonded gel-elastomer interfaces may accommodate swelling or shrinkage without substantially constraining device deformation [27]. These strategies may also protect device-side connections while preserving the skin-facing or analyte-facing function of the gel.

At the manufacturing level, future development could extend beyond optimizing individual formulations toward reproducible device production. Control of factors such as precursor composition and rheology, filler dispersion, crosslinking conditions, solvent content, device geometry, and interface formation could reduce batch-to-batch and device-to-device variation. Packaging and storage conditions may also need to preserve material and interfacial properties over the intended shelf life. Fabrication methods supporting scale-up potential, such as screen or inkjet printing, coating, molding, fiber or textile processing, and three-dimensional printing, may support patterned, arrayed, or anatomically customized systems, while process simplification, modular architectures, and separable or recyclable components may reduce manufacturing complexity [312,313,314]. Studies for scalable fabrication could prioritize fabrication yield and batch-to-batch variation in gel thickness, solvent content, electrical properties, and interfacial adhesion. Sustainability assessment could quantify material and solvent recovery, retained performance after repeated recycling, and processing energy, while examining whether the gel, electrodes, and encapsulation can be separated at the end of use. Practical reliability is likely to depend on reproducible fabrication and stable operation of complete devices rather than on favorable properties measured only in isolated gel specimens.

### 6.4. Standardization and Future Healthcare Platforms

As material and device designs become more complex, standardized validation that remains relevant to intended use is likely to become increasingly important for meaningful comparison and practical translation. A single universal performance threshold is unlikely to be appropriate because conductive gel applications such as electrophysiological electrodes, mechanical and biochemical sensors, wound-monitoring platforms, and human–machine interfaces operate under different conditions and serve different functions. Therefore, standardization may be most useful when it establishes a common reporting framework while allowing test conditions and acceptance criteria to remain application specific [315]. Relevant reference procedures include ASTM D412 for elastomer tensile testing and ASTM F2255 for tissue-adhesive lap-shear testing, with their applicability and any adaptations for hydrated gels and selected substrates explicitly documented [316,317]. Reporting could specify gel composition and solvent content, specimen and electrode geometry, preconditioning, loading amplitude and rate, cycle count, temperature, humidity, sweat composition and pH, and exposure duration. A common testing sequence could include baseline characterization, separate and combined mechanical and sweat exposure, and reassessment after a defined recovery period, using matched unexposed controls.

As a proposed minimum reporting dataset, studies could provide baseline calibration or impedance spectra, time-resolved outputs during exposure, post-exposure recovery, and individual results from independently fabricated devices, together with the numbers of devices and batches, variability, failures, and data-processing methods [276,298,318]. Application-specific endpoints could include impedance magnitude and phase and SNR for bioelectrodes [318], hysteresis for mechanical sensors [90], and calibration drift and selectivity for chemical or biochemical sensors [276]. Test duration and acceptance criteria would remain linked to intended use [298], while accelerated testing would require justification that it reproduces relevant degradation mechanisms [319].

To support practical translation, future on-body studies could extend beyond short measurements performed under stationary or tightly controlled conditions. Reproducibility could be examined across factors such as users and wearing sessions, with testing performed at the intended anatomical sites and under representative body motion, perspiration, and environmental conditions. Such studies would help determine whether calibration and signal interpretation remain valid during realistic use. Comparisons with suitable reference methods, including commercial or clinical instruments where relevant, could clarify whether the device output accurately represents the intended physiological or biochemical variable. For devices intended to support clinical decisions such as diagnosis, treatment selection, or disease management, clinical relevance is likely to depend on longitudinal studies involving relevant patient populations [318]. For applicable medical-device investigations, alignment with ISO 14155 could support prospective studies with prespecified performance and safety endpoints, justified sample sizes, and comparisons against appropriate clinical reference methods [320].

These validation efforts can support regulatory evaluation when coordinated with biological assessment within the ISO 10993-1 framework and aligned with applicable FDA guidance and device-specific requirements [71]. Identifying the applicable regulatory pathway can help clarify the safety and performance evidence needed to support medical-device market access. Under the FDA regulatory framework, these pathways may include 510(k) clearance based on substantial equivalence, De Novo classification for eligible novel low- or moderate-risk devices, and premarket approval (PMA) for high-risk devices. The appropriate pathway depends on intended use, risk classification, and predicate availability rather than wearable or implantable form alone [321,322,323]. Early consideration of these requirements may help guide the development and practical translation of future healthcare platforms.

Future healthcare platforms are expected to integrate conductive gel sensing interfaces with technologies such as flexible circuitry, wireless data acquisition and transmission, adaptive calibration, and intelligent data analytics. Data-driven methods, including machine learning, may support functions such as drift compensation, artifact suppression, multimodal signal decoupling and fusion, and the interpretation of physiological signals for health monitoring, decision support, or feedback control [324,325]. A specific research priority is to validate multimodal signal decoupling and adaptive calibration using data from users, recording sessions, and environmental conditions excluded from the training dataset [326,327]. Comparisons with individual sensing channels and conventional calibration methods could help determine whether improvements in accuracy and robustness under changing physiological and environmental conditions justify the added computational complexity [328]. Although these computational approaches can mitigate certain sources of drift and interference, they are unlikely to recover information lost or distorted through material degradation, unstable biointerfaces, inadequate selectivity, or calibration models that do not represent the underlying sensing mechanism. Therefore, their greatest value may lie in refining an already reliable sensing system rather than compensating for fundamental limitations in its material, interface, or sensing design.

Beyond data acquisition and analysis, integration with technologies such as low-power electronics, power-management circuitry, and energy harvesting or storage components may extend operating duration and reduce dependence on external power sources. For energy harvesting in particular, a specific priority is to quantify the net energy available after conversion and storage losses under realistic active and resting conditions and to determine whether it can support the required duty cycles for sensing, computation, and wireless transmission in the complete device [329,330]. Closed-loop platforms could also couple sensing outputs to real-time feedback or therapeutic actuation, including electrical stimulation and controlled drug delivery [225,331]. Together, these developments could broaden the role of conductive gels beyond stand-alone sensing toward functional components of more responsive healthcare platforms.

To realize these broader capabilities, the immediate research priority is to establish sustained electrical and mechanical performance under combined conditions of sweat exposure and repeated deformation, with particular attention to skin–gel and gel–device interfacial reliability. A second priority is to achieve reproducible fabrication across batches and to establish standardized testing of complete devices, thereby enabling reported improvements to be independently reproduced and compared. For medical applications, clinical validation in the intended patient populations, guided from the outset by application-specific safety and regulatory requirements, is essential to establish practical healthcare value. Accordingly, translating conductive gels from laboratory demonstrations into dependable wearable and attachable healthcare technologies is likely to depend on application-specific system integration that balances considerations such as long-term reliability, manufacturability, user safety, and system-level performance, rather than on maximizing any single material or sensing metric in isolation.

## Figures and Tables

**Table 2:** Representative conductive gel-based transduction systems grouped by sensing mode.

Sensing Mode and Transduction	Dominant Electrical Signal	Sensitivity	Operating Range	Limitation	Suitable Applications	Refs
Resistive mechanical sensing	Relative resistance change, ΔR/R_0_	(a) GF = 9.21 (at 100% strain) (b) GF = 3.18 (25–175% strain) (c) GF = 2.33 (0–350% strain) (d) GF = 0.22 (at 100% strain)	(a) 0–100% strain (b) 1–200% strain (piecewise) (c) 0–2100% strain (piecewise) (d) 0–200% strain (cyclic sensing)	Sensitivity-stretchability trade-off; nonlinear response; temperature/moisture cross-sensitivity.	Joint motion, respiration and pulse monitoring; multimodal e-skin.	(a) [72] (b) [78] (c) [84] (d) [109]
Conventional capacitive mechanical sensing	Relative capacitance change, ΔC/C_0_	(a) (Strain) GF ≈ 1 (derived from C = C_0_λ) (b) (Strain) GF = 0.487 (c) (Pressure) 0.42 kPa^−1^	(a) 0–100% strain (b) 0–204% strain (c) 0–200 Pa pressure (calibration)	Strain-rate dependence; dehydration-related changes in gel conductivity.	Joint motion tracking, robotic control and airflow-pressure monitoring.	(a) [92] (b) [90] (c) [62]
EDL-based ionotronic mechanical sensing	Interfacial capacitance change, ΔC/C_0_	(a) 3302.9 kPa^−1^ (below 10 kPa) (b) 2.47 kPa^−1^ (at 70% RH) (c) 810 kPa^−1^ (0–40 kPa)	(a) 0–360 kPa (piecewise; 1 kHz) (b) 0–2000 kPa (linear) (c) 0–440 kPa (piecewise; 1 kHz)	Temperature-dependent ionic response; preload-dependent pressure resolution.	Pulse monitoring, tactile mapping and joint-pressure measurement.	(a) [73] (b) [93] (c) [129]
Thermal sensing through resistive transduction	Temperature-dependent resistance	(a) |TCR| = 5.27% °C^−1^ (0–30 °C) (b) |TCR| = 8.4% °C^−1^ (at 300 K) (c) |TCR| = 1.67% °C^−1^	(a) 0–80 °C (piecewise) (b) 25–80 °C (c) ~20–60 °C (calibration)	Moisture-induced signal drift; encapsulation can slow thermal response.	Skin and surface temperature monitoring; thermal feedback.	(a) [77] (b) [102] (c) [62]
Moisture-responsive sensing/ C onductometric transduction	Relative conductance change, ΔG/G_0_	17.6/67.0% per % RH (piecewise)	11–98% RH	Nonlinear response over the full humidity range.	Wireless respiration monitoring.	[74]
Moisture-responsive sensing/ I mpedimetric transduction	Humidity-dependent impedance	2.0% per % RH (20 °C)	30–90% RH (400 Hz)	Temperature cross-sensitivity requires compensation.	Temperature- compensated humidity sensing.	[106]
Moisture-responsive/I onic-gradient transduction (g enerator)	Open-circuit voltage; short-circuit current density	-	10–90% RH (characterization)	Output decreases near moisture saturation and varies with temperature.	Moisture-based energy harvesting.	[103]
Chemical sensing/ C onductometric pH response	Electrical conductivity	~2.6 × 10^−4^ S∙m^−1^∙pH^−1^ * (average)	pH 2–8 (nonlinear response)	Swelling- and composition-dependent conductivity.	pH-responsive switches and logic gates.	[114]
Chemical sensing/ Potentiometric pH transduction	Electrode potential	65.4 mV∙pH^−1^ (calibration)	pH 2–8	Limited validation under variable wear conditions.	Sweat pH monitoring.	[130]
Chemical sensing/ Electrochemical O_2_ transduction	Relative current change, ΔI/I_0_	0.2% ppm^−1^ (2–20% O_2_)	0–100% O_2_ (overall; linear fit at 2–20%)	Temperature-, humidity- and strain-dependent response.	Exhaled-breath oxygen monitoring.	[67]
Biochemical sensing/ Affinity-based voltammetry	DPV peak current	8.929 μA∙decade^−1^ (CEA)	1 fg∙mL^−1^- 200 ng∙mL^−1^	Assay pH and incubation requirements.	CEA assays in human serum.	[112]
Biochemical sensing/ Enzymatic amperometry	Normalized current response, NR	~0.27 decade^−1^ * (0% strain)	50 μM-10 mM (glucose)	Strain-dependent sensitivity; sweat sampling and sweat-blood correlation constraints.	Sweat-glucose monitoring.	[126]
Biochemical sensing/ Affinity-based impedimetry	Change in charge-transfer resistance, ΔR_ct_	5.2 kΩ∙decade^−1^ (AβO; plasma)	0.1 pM-10 nM (AβO)	Storage-related hydrogel collapse and signal drift.	Aβ oligomer detection	[131]
Biochemical sensing/ Enzymatic conductometry	Conductance change	597 μS∙μM^−1^ (OTA)	Up to 60 nM (OTA; linear)	Diffusion through the polymer membrane slows the response.	OTA screening	[132]

* Estimated from the graphs reported in the cited references. Sensitivity and operating ranges refer to the cited examples under their reported test conditions. Direct comparison is limited by differences in target stimuli or analytes, signal definitions, normalization, and measurement protocols. The limitations and suitable applications summarize general considerations for the sensing modes discussed in this review. Their relevance depends on factors such as gel composition, device design, interfacial stability, cross-sensitivity, and the intended operating conditions and duration of use. Abbreviations: GF: gauge factor; TCR: temperature coefficient of resistance; EDL: electric double layer; RH: relative humidity; R: resistance; C: capacitance; G: conductance; I: current; NR: normalized current response; R_ct_: charge-transfer resistance; DPV: differential pulse voltammetry; CEA: carcinoembryonic antigen; AβO: amyloid-beta oligomers; OTA: ochratoxin A.

## Data Availability

No new data were created or analyzed in this study. Data sharing is not applicable to this article.
